# Association between serum uric acid levels and myasthenia gravis: A meta-analysis

**DOI:** 10.1097/MD.0000000000045364

**Published:** 2025-10-17

**Authors:** Lang Liu, Tong Yang, Xingli Sun, Xi Zhang, Jiangqin Ou

**Affiliations:** a Guizhou University of Traditional Chinese Medicine, Guiyang, P.R. China; b The First Affiliated Hospital of Guizhou University of Traditional Chinese Medicine, Guiyang, P.R. China.

**Keywords:** case-control, meta-analysis, myasthenia gravis, risk, uric acid

## Abstract

**Background:**

Serum uric acid (UA) levels may be involved in the development of myasthenia gravis (MG) by inhibiting oxidative stress, but the relationship remains unclear. This meta-analysis aimed to assess the association between serum UA levels and MG patients.

**Methods:**

According to the established protocol, researchers searched 9 databases for studies on UA levels in MG patients, assessed using the Newcastle–Ottawa Scale. Heterogeneity was evaluated with the *I*^2^ statistic and chi-square test. Publication bias was analyzed using funnel plots and Egger test.

**Results:**

This meta-analysis included 9 case-control studies from China, with 2112 participants (955 MG patients, 1157 healthy controls). All studies had Newcastle–Ottawa Scale quality scores of 7 or above. Results showed significantly lower serum UA levels in MG patients compared to controls (*I*^2^ = 58%, mean difference [MD]: −43.86, 95% confidence interval [CI]: [‐54.98, −32.74], *P* < .00001). Age differences were identified as a source of heterogeneity, confirmed by subgroup analysis. Subgroup analyses showed that in age-comparable groups, MG and healthy controls had lower heterogeneity in UA levels (*I*^2^ = 18%, MD: −36.57, 95% CI: [‐44.62, −28.50], *P* < .00001), and in age-disparate groups (*I*^2^ = 0%, MD: −73.78, 95% CI: [‐94.13, −53.44], *P* < .00001). Gender analyses showed UA levels in men (*I*^2^ = 69%, MD: −60.29, 95% CI: [‐81.75, −38.83], *P* < .00001) and women (*I*^2^ = 1%, MD: −29.80, 95% CI: [‐38.03, −21.57], *P* < .00001).

**Conclusion:**

Lower serum levels of UA are associated with an increased risk of MG, although further large-scale, well-controlled studies are needed to confirm the potential clinical relevance.

## 1. Introduction

Myasthenia gravis (MG) is a chronic autoimmune neuromuscular disorder characterized by fatigable muscle weakness, which typically worsens with activity and improves with rest. The most common initial symptoms are ocular, such as diplopia and ptosis, and the majority of patients progress to generalized muscle weakness within 24 months of disease onset.^[1,2]^ The incidence of MG ranges from 4.1 to 30 cases per million person-years, with a prevalence of 150 to 200 cases per million.^[3]^ The pathogenesis of MG is complex and multifactorial, involving genetic predisposition, immune dysregulation, and environmental triggers. MG significantly impairs patients’ quality of life, affecting their daily activities and work performance.

The diagnosis of MG typically relies on a combination of clinical evaluation, serological testing for specific autoantibodies, such as acetylcholine receptor (AChR) and muscle-specific kinase antibodies, as well as electrophysiological tests like repetitive nerve stimulation and single-fiber electromyography.^[4]^ While these diagnostic approaches are useful, they may have limitations, including variability in sensitivity and specificity. The fluctuating nature of MG and the heterogeneity of clinical features further complicate early diagnosis and disease monitoring. Despite advances in treatment, including immunosuppressive therapy, acetylcholinesterase inhibitors, plasma exchange, and thymectomy, many patients experience disease recurrence or side effects from long-term medications.^[5]^ To date, the pathogenesis and associated risk factors of MG remain incompletely understood and require further investigation. Beyond the well-established role of autoantibodies in MG pathogenesis, other systemic contributors to disease onset and progression, such as oxidative stress, have drawn increasing attention in recent years.

Uric acid (UA), the end product of purine metabolism, has been proposed to play a role in various neurological and autoimmune conditions due to its antioxidant properties. Lower serum UA levels are thought to exacerbate oxidative stress, potentially amplifying immune dysregulation and contributing to neuronal damage.^[6]^ Considering that MG is an autoimmune neuromuscular disease, previous studies have suggested that oxidative stress may be involved in the pathogenesis of this condition, while UA as an antioxidant may play a role in the progression of MG. Studies have reported an association between UA levels and various diseases, including multiple system atrophy,^[7]^ multiple sclerosis,^[8]^ and Parkinson disease.^[9]^ However, the association between serum uric acid levels and myasthenia gravis has yet to be systematically investigated.

Although some studies have attempted to explore the relationship between UA levels and MG, the specific role of UA in the onset and progression of MG remains inconclusive. This meta-analysis aims to systematically evaluate the association between serum UA levels and MG, addressing a critical gap in the current literature. By investigating the potential correlation between UA and MG, we aim to elucidate the risk of MG development and its underlying pathogenic mechanisms. A deeper understanding of this relationship could provide valuable insights into the molecular mechanisms and risk factors underlying MG, facilitate the identification of potential biomarkers for early screening, prevention, and diagnosis, and offer new perspectives and insights for the development of novel therapeutic strategies targeting MG.

## 2. Materials and methods

### 2.1. Search strategy

A systematic literature search was conducted on literature published up to July 28th, 2024. Concerning the association of serum UA levels with the risk of MG using PubMed, Web of Science, Embase, Cochrane Library, Wanfang Database, VIP Database, China Biology Medicine Disc and China National Knowledge Infrastructure. No language restriction was set. Titles and abstracts were searched using the terms (“myasthenia gravis,” “uric acid,” “hypouricemia,” “urate,” the search string for the PubMed database is: (“myasthenia gravis”[Title/Abstract]) AND (“uric acid”[Title/Abstract] OR “hypouricemia”[Title/Abstract] OR “urate”[Title/Abstract]). Two independent investigators (LangLiu and TongYang) evaluated the studies that potentially met the inclusion criteria.

### 2.2. Study inclusion and exclusion criteria

All included studies must meet the following criteria: The diagnostic standards for MG primarily reference the Drachman criteria^[10]^ or the Chinese diagnostic criteria^[11]^; The study design is confined to case-control studies, cohort studies, or cross-sectional studies; The control group consists of healthy subjects; Focus upon assessment of the relationship between serum UA level and MG; Serum UA levels were evaluated with appropriate methods.

The following publications were excluded from the analysis for the reasons stated: Studies concentrating on non-serum UA, such as cerebrospinal fluid UA; Studies that failed to report precise values of serum UA levels.

### 2.3. Data extraction and article quality analysis

Two investigators independently evaluated the eligibility of potentially relevant studies and extracted key information. Any discrepancies in the extracted data were discussed with a third researcher until a consensus was reached through careful deliberation. The extracted data included the authors’ names, publication year, language of publication, region, the mean serum UA levels for cases and controls, standard deviation, sample size, sex, age, and other pertinent information. the included studies was evaluated using the Newcastle–Ottawa Scale (NOS) to assess the overall quality of the literature and the credibility of the conclusions. Studies with an overall NOS score of ≥7 were considered high quality.^[12]^

### 2.4. Statistical analysis

We used mean difference (MD) and 95% confidence interval (CI) to evaluated the relationship between serum UA level (μmol/L) and the risk of MG. Heterogeneity analysis was performed using the chi-square test and the *I*^2^ statistic, with a *P*-value <.05 for the chi-square test indicating statistically significant heterogeneity, and an *I*^2^ value ≥ 50% indicating the presence of heterogeneity.^[13]^ When no significant heterogeneity was present, MD were pooled using the fixed effects model, when significant heterogeneity was detected, the random effects model was employed. Subgroup analyses and sensitivity analysis were used for exploring the source of heterogeneity. This meta-analysis was performed using Review Manager (RevMan version 5.4, The Cochrane Collaboration). Publication bias was assessed using Egger test in Stata 18.0 (Stata Corp LP, College Station, TX).

## 3. Results

### 3.1. Search results and characteristics, quality of the included studies

**Figure 1.**
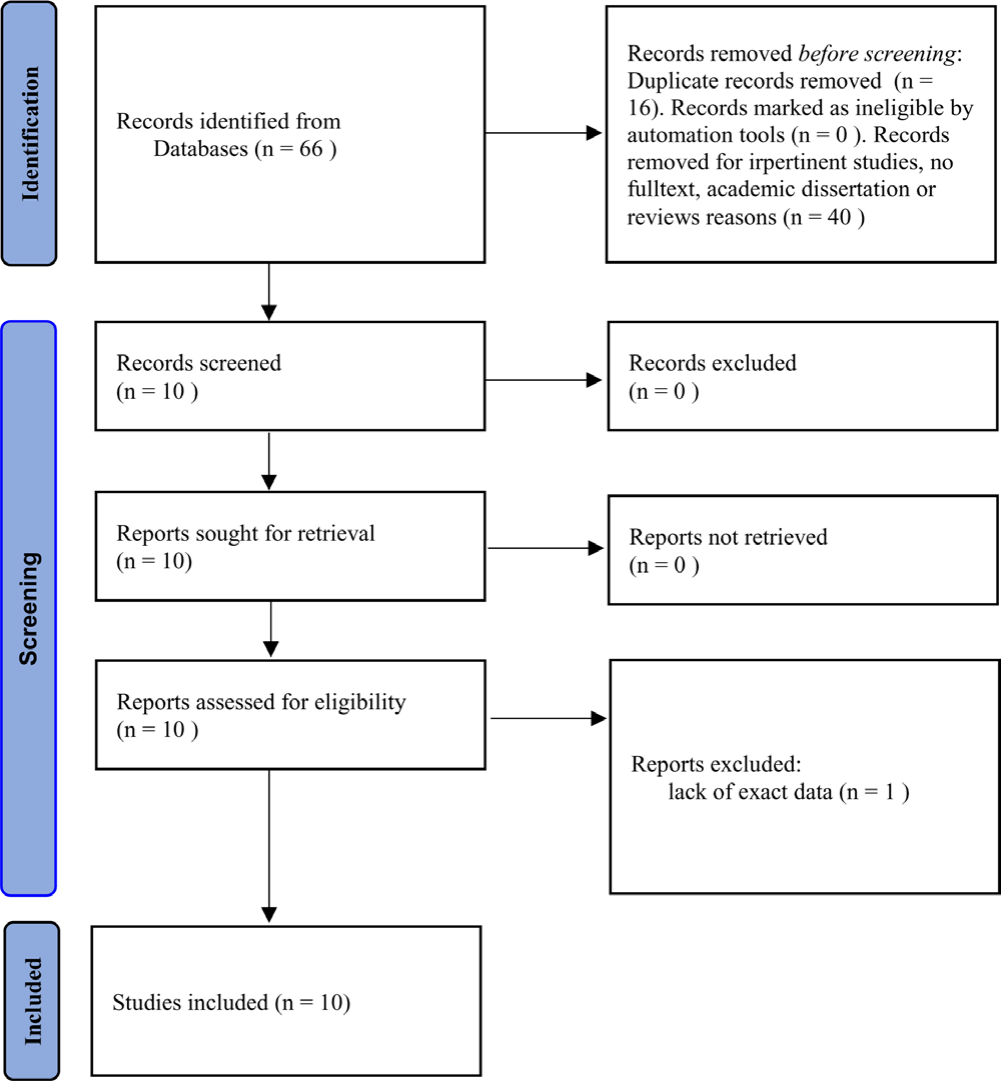
Flow diagram of the study selection process in the meta-analysis.

Based on the previously established search strategy and criteria, all potential studies were included. After excluding duplicate publications and studies lacking precise UA data, a total of 9 studies were ultimately identified for meta-analysis,^[14–22]^ 4 articles are published in Chinese,^[14,16,17,22]^ and the rest are in English. The full texts of the 9 included studies can be found in Data S1, Supplemental Digital Content, https://links.lww.com/MD/Q443. The study selection process is illustrated in the flow chart (Fig. 1). All studies were case-control studies concentrated on Chinese populations. A summary of the data extracted from these studies is compiled in Table 1.

**Table 1:** The characteristics of the included studies.

Study, year	Category	N	Male	Female	Age (yr)	UA level (μmol/L)	NOS score	Region
Huang et al^[14]^	MG	20	9	11	31.25 ± 20.17	268 ± 72	8	China
	Control	20	8	12	32.6 ± 19.54	310 ± 69		
Jiang et al^[15]^	MG	132	62	70	2.92 (median)	238.16 ± 66.54	8	China
	Control	140	67	73	3.42 (median)	265.27 ± 73.87		
Li et al^[16]^	MG	31	12	19	18.35 ± 13.03	262.75 ± 70.84	8	China
	Control	31	12	19	16.33 ± 12.57	298.49 ± 76.57		
Liu et al^[17]^	MG	221	79	142	48.55 ± 16.79	298.75 ± 83.87	8	China
	Control	198	70	128	48.35 ± 16.65	328.63 ± 62.48		
Peng et al^[18]^	MG	42	22	20	28.8 (mean)	237.2 ± 76.3	7	China
	Control	89	43	46	41 (mean)	312.1 ± 92.8		
Peng et al^[19]^	MG	77	40	37	32.91 ± 20.20	266.03 ± 93.09	7	China
	Control	133	73	60	46.99 ± 16.47	338.87 ± 107.1		
Yang et al^[20]^	MG	166	72	94	42.59 ± 15.66	283.1 ± 90.35	8	China
	Control	214	99	115	43.68 ± 14.15	333.83 ± 87.68		
Yang et al^[21]^	MG	135	57	78	41.65 ± 15.80	283 ± 90	8	China
	Control	156	69	87	42.90 ± 14.30	335 ± 84		
Zou et al^[22]^	MG	131	68	63	45.00 ± 19.00	270 ± 70	8	China
	Control	176	96	80	46.00 ± 15.00	301 ± 60		

MG = myasthenia gravis, N = total sample size, NOS = Newcastle–Ottawa Scale, UA = uric acid.

### 3.2. Quality of the included studies

The quality assessment of the studies included in the final meta-analysis showed that the NOS scores ranged from 7 to 8, indicating that the total quality of the methodologies was generally good (Table 1).

### 3.3. Meta-analysis results

**Figure 2.**
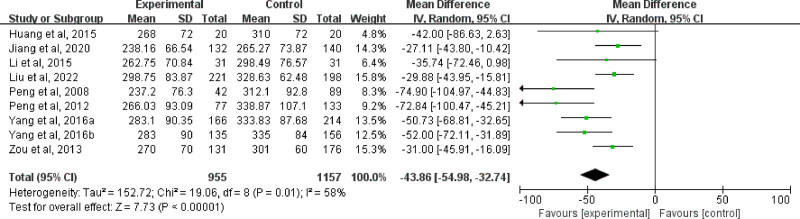
Total UA levels between MG patients and healthy controls. MG = myasthenia gravis, UA = uric acid.

The meta-analysis results of overall UA levels indicated a significant heterogeneity among the studies (*I*^2^ = 58%; *P* = .01). Therefore, a random effects model was applied, the pooled MD is −43.86 (95% CI: [‐54.98, −32.74], *P* < .00001), showing that UA levels in MG patients were significantly lower than those in healthy controls (Fig. 2).

### 3.4. Publication bias

Publication bias was assessed using Egger test, and no significant publication bias was detected (*P* = .102).

### 3.5. Sensitivity analysis and heterogeneity exploration

We conducted a sensitivity analysis by omitting individual studies and found that serum UA levels in MG patients were significantly lower than those in the healthy control group.

**Figure 3.**
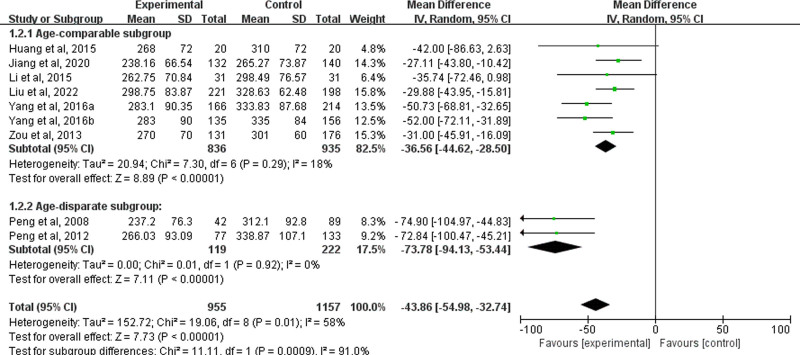
Subgroup analysis of serum UA levels related to age differences. UA = uric acid.

To investigate the sources of heterogeneity, we analyzed information from the included studies and identified significant age differences between the MG and control groups in the studies by Peng et al.^[18,19]^ This age disparity could be a potential source of heterogeneity. Consequently, we performed a subgroup analysis, which showed that after excluding these 2 studies, heterogeneity decreased (*I*^2^ = 18%), the pooled MD is −36.56 (95% CI: [‐44.62, −28.50], *P* < .00001), between age-disparate groups with MG and healthy control groups the pooled MD is −73.78 (*I*^2^ = 0%, 95% CI: [‐94.13, −53.44], *P* < .00001), confirming the stability of the results, showed that MG patients had significantly lower UA levels than healthy controls (Fig. 3).

### 3.6. Analysis of sex differences in UA levels

**Figure 4.**
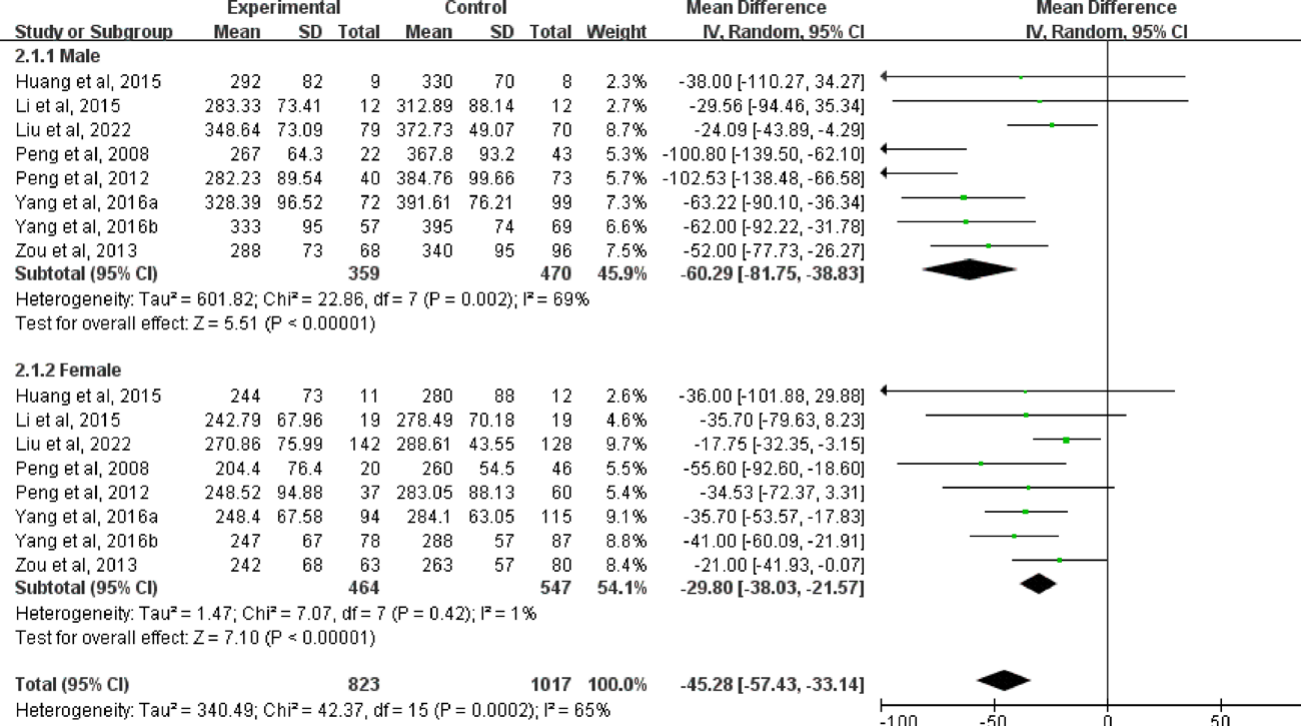
Subgroup analysis of serum UA levels in males and females. UA = uric acid.

A total of 8 studies provided data on sex differences in UA levels. The results of the random effects model meta-analysis indicated heterogeneity among males (*I*^2^ = 69%), with a combined MD of −60.29 (95% CI: [‐81.75, −38.83], *P* < .00001), UA levels in males were significantly lower than those in healthy controls. Among females, heterogeneity was minimal (*I*^2^ = 1%), the combined MD is −29.80 (95% CI: [‐38.03, −21.57], *P* < .00001). UA levels in females were significantly lower than those in healthy controls (Fig. 4).

## 4. Discussion

In this meta-analysis, we included 9 studies to compare serum UA levels between individuals with MG and healthy controls. Our findings demonstrated that UA levels in MG patients are significantly lower than those in healthy controls. This association remained consistent in sensitivity analyses and sex-based subgroup analyses, suggesting that our conclusion is robust and reliable. However, due to the presence of confounding factors and heterogeneity, these findings should be interpreted with caution. The observed heterogeneity among studies may partially reflect differences in participant age, as age-related variation in serum UA levels has been well-documented.^[23]^ Additionally, 4 studies compared UA levels among MG patients with different disease subtypes based on the MGFA clinical classification and Osserman classification systems.^[19–22]^ All 4 studies consistently reported that UA levels tended to decrease as MG severity increased, potentially reinforcing the association between UA levels and MG. However, due to differences in classification systems and limited availability of relevant data, we were unable to perform a meta-analysis to provide a unified assessment.

MG is an autoimmune neuromuscular disorder characterized by impaired neuromuscular transmission, one of the elucidated pathogenic mechanisms of which involves autoantibody-mediated abnormalities targeting the nicotinic AChR and other components of the neuromuscular junction, leading to symptoms such as muscle weakness.^[24,25]^ AChR, a transmembrane ion channel located on the muscle cell membrane, is crucial for neuromuscular transmission. Studies have shown that increased oxidative stress is associated with the destruction of autoimmune tissues,^[26]^ and a decrease in antioxidant reserves may represent an early pathogenic mechanism of MG. Oxidative stress can damage AChR and other cellular components,^[27]^ which may exacerbate the autoimmune process in MG. Oxidative stress is a pathological condition caused by the excessive production of reactive oxygen species (ROS) and reactive nitrogen species (RNS) or a reduction in the functionality of antioxidant systems. It acts through multiple levels, such as molecular, cellular pathways, and gene regulation.^[28]^ ROS, such as superoxide anion (O_2_**·**^‐^) and hydroxyl radical (**·**OH), and RNS, such as peroxynitrite (ONOO^‐^), can directly attack AChR, leading to protein oxidation, nitration, and degradation.^[29]^ ROS can induce lipid peroxidation, compromising the integrity of neuromuscular junction cell membranes. This membrane damage reduces the sensitivity of AChR to acetylcholine, potentially aggravating muscle weakness symptoms in MG.^[30]^ Oxidative stress activates various signaling pathways, such as nuclear factor κB, amplifying immune and inflammatory responses.^[31]^ The accumulation of ROS damages mitochondrial function by directly attacking mitochondrial DNA, causing mutations or deletions and reducing ATP production.^[32,33]^ Furthermore, ROS accumulation induces oxidative stress damage to skeletal muscle fibers. Oxidative modifications of key proteins, such as myosin and actin, reduce muscle contractility, while lipid peroxidation in skeletal muscle cell membranes compromises membrane integrity, exacerbating muscle fatigue and weakness.^[34,35]^

UA, as the final product of purine metabolism, has a chemical structure of 2,6,8-trihydroxypurine (C_5_H_4_N_4_O_3_) and is primarily produced through the breakdown of purine nucleotides.^[36]^ In humans, the production of UA relies on both endogenous and exogenous pathways.^[37]^ In the endogenous pathway, purine nucleotides such as adenine and guanine are metabolized into hypoxanthine and xanthine, which are subsequently converted into UA under the catalytic action of xanthine oxidase. The exogenous pathway is closely related to the purine content in dietary intake.^[38]^ UA is mainly excreted through the kidneys and intestines,^[39]^ and renal urate transporters, such as URAT1 and GLUT9, play significant roles in the excretion and reabsorption of UA.^[40]^

In addition to being the terminal product of purine metabolism, UA possesses important antioxidant properties. It scavenges ROS and RNS, including superoxide anions (O_2_**·**^‐^) and peroxynitrite (ONOO^‐^), thereby reducing oxidative stress and mitigating neuronal damage. It may prevent oxidative damage to AChR and other NMJ proteins.^[41,42]^ Moreover, UA may activate the Nrf2-ARE (nuclear factor erythroid 2-related factor 2–antioxidant response element) pathway, regulating the expression of antioxidant enzymes such as glutathione peroxidase and superoxide dismutase, thereby enhancing cellular antioxidant capacity.^[43,44]^

UA may be significantly depleted under conditions of oxidative stress, leading to reduced UA levels. Previous studies have suggested that UA plays a protective role in neurological and immune-mediated diseases. For instance, lower UA levels have been observed in multiple sclerosis and Parkinson disease.^[45]^ Sustained production of ROS and RNS can lead to rapid UA depletion as it neutralizes these reactive molecules. Reduced UA levels may indicate a state of oxidative stress in patients.^[46,47]^ However, it remains unclear whether this reduction is a cause or consequence of disease. In MG, reduced UA levels may result from increased consumption due to oxidative stress or abnormalities in the purine metabolism pathway. Future studies are needed to investigate the specific roles of UA in MG, including its regulation of purine metabolism, interactions with the immune system, and its impact on NMJ function.

## 5. Conclusion

Our meta-analysis indicates that reduced serum UA levels are negatively associated with MG onset. We hypothesize that this may be related to the regulatory role of UA in oxidative stress. However, other unexplored mechanisms and pathways may also contribute to this phenomenon. These findings suggest that serum UA levels could potentially serve as a biomarker for early screening or disease monitoring in MG, aiding early diagnosis and disease surveillance. Furthermore, interventions aimed at regulating UA levels may provide new therapeutic strategies for MG patients.

However, several limitations of this study should be acknowledged. First, the primary limitation is the limited number of studies included. Although we conducted a comprehensive search of published literature, the data obtained were limited, and all study populations were from China, lacking representation from other regions and ethnicities. Second, we noticed that 4 studies conducted by 2 research teams were included in our analysis,^[18–21]^ and it is unclear whether there was duplication of cases, which could affect the effect size. Third, the included studies comprised both outpatient and hospitalized cases. Generally, hospitalized patients tend to have more severe disease, which may have influenced the results. Fourth, there were age differences among the included case-control studies, which encompassed both pediatric and adult populations. This variability in age may have affected the conclusions. Fifth, our results indicate that the mean UA levels in MG patients were lower than those in matched healthy controls. However, since the UA levels in both groups remained within the normal range, this finding provides limited evidence to fully explain the observed difference. Sixth, several confounding factors that might influence serum UA levels, such as disease severity, physical activity, medication use, comorbidities, and diet, were not fully considered in our analysis. Seventh, the impact of UA on MG pathogenesis remains speculative and requires further experimental validation.

Therefore, we recommend conducting more comprehensive and rigorous case-control, cross-sectional, and cohort studies in the future to update meta-analyses. Experimental research is also necessary to explore the relationship between serum UA levels and MG, as well as the underlying mechanisms through which UA influences disease progression.

## Author contributions

**Writing – original draft:** Lang Liu, Tong Yang, Xingli Sun.

**Writing – review & editing:** Xi Zhang, Jiangqin Ou.

## Supplementary Material


