# Supplementary material for: Association between serum uric acid levels and myasthenia gravis: A meta-analysis
Source: Medicine (Baltimore). 2025 Oct 17;104(42):e45364. doi: 10.1097/MD.0000000000045364 (PMC12537177; doi:10.1097/MD.0000000000045364)
Supplement: Supplementary file 1 [file medi-104-e45364-s001.pdf]

·重症肌无力·

重症肌无力患者血清胆红素和尿酸变化及其意义

周霞　孙中武

【摘要】 **目的**　通过对比重症肌无力(MG)患者与正常人群的血清胆红素(BIL)和尿酸(UA)水平,分析 BIL 和 UA 在不同性别、不同分型及有无伴随胸腺增生/胸腺瘤 MG 患者之间的关系,进一步探讨血清 BIL 和 UA 在 MG 中的变化及其意义。**方法**　选取 2010 年 1 月至 2012 年 12 月在安徽医科大学第一附属医院神经内科住院的 MG 患者 131 例,其中男 68 例,女 63 例,对照组 176 名为年龄和性别相匹配的同期健康体检正常人群,其中男 96 名,女 80 名。采用氧化酶定量分析法测定血清 BIL 及 UA 值,观察两组直接胆红素(DBIL)、间接胆红素(IBIL)、总胆红素(TBIL)及 UA 值之间的差异。分别对不同性别、不同类型及有无伴随胸腺异常改变 MG 患者的各项指标进行比较,并对影响血清 BIL 及 UA 水平的各指标进行相关分析。**结果**　MG 患者的血清 IBIL、TBIL 及 UA 值[(7.4 ± 0.3) μmol/L,(11.0 ± 0.4) μmol/L,(270 ± 70) μmol/L]均显著低于正常对照组[(9.6 ± 0.3) μmol/L,(13.0 ± 0.4) μmol/L,(301 ± 60) μmol/L],差异有统计学意义($P<0.01$)。将两组受试者按不同性别分别比较时这种改变在男性和女性 MG 患者中均存在,且女性 MG 患者血清各指标值[(6.7 ± 2.7) μmol/L,(10.1 ± 3.7) μmol/L,(242 ± 68) μmol/L]均显著低于男性[(8.4 ± 4.2) μmol/L,(12.2 ± 5.5) μmol/L,(288 ± 73) μmol/L],差异有统计学意义($P<0.05 \sim 0.01$)。不同类型 MG 患者及有无伴随胸腺异常改变 MG 患者血清 DBIL、IBIL、TBIL 及 UA 水平之间无明显差异($P>0.05$)。多元逐步 Logistic 回归分析显示在校正年龄、吸烟、性别、BMI、BUN、Cr、HDL-C 及 LDL-C 等相关因素后,低 TBIL 水平者(TBIL < 11.4 μmol/L)患 MG 的风险是高 TBIL 水平的 1.98 倍;低 UA 水平者(UA < 279 μmol/L)患 MG 的风险是高 UA 水平的 2.22 倍。相关分析发现 TBIL 与 Cr($r=0.151, P=0.008$)、UA 与 Cr、HDL-C($r=0.301, P=0.000, r=-0.347, P=0.000$)间存在一定的相关关系。**结论**　MG 患者血清 IBIL、TBIL 和 UA 水平均显著低于正常健康人群,此为将来防治 MG 的发生发展提供了新的思路和依据。

【关键词】 重症肌无力;　胆红素;　尿酸

Changes of serum bilirubin and uric acid in patients with myasthenia gravis

ZHOU Xia, SUN Zhong-wu. Department of Neurology, First Affiliated Hospital, Anhui Medical University, Hefei 230022, China
Corresponding author: SUN Zhong-wu, Email: sunzhwu@hotmail.com

【**Abstract**】 **Objective**　To explore the correlations of serum total bilirubin (TBIL), direct bilirubin (DBIL), indirect bilirubin (IBIL) and uric acid (UA) with myasthenia gravis (MG). **Methods**　A total of 131 MG patients were selected as MG group and 176 healthy cases as control group. They were enrolled from the Department of Neurology, First Affiliated Hospital, Anhui Medical University between January 2010 and December 2012. And the controls were recruited from a health check-up center. The total serum BIL and UA concentrations were measured by an enzymatic method. All MG patients underwent thymus computed tomography (CT) scanning. **Results**　The serum levels of TBIL, IBIL and UA in patients with MG ((11.0 ± 0.4, 7.4 ± 0.3, 270 ± 70) μmol/L) were significantly lower than those in healthy control group ((13.0 ± 0.4, 9.6 ± 0.3, 301 ± 60) μmol/L) ($P<0.01$). Moreover, these results were consistent when the male and female cohorts were investigated separately. In MG group, females had significantly lower serum TBIL, IBIL and UA levels ((10.1 ± 3.7, 6.7 ± 2.7, 242 ± 68) μmol/L) than males ((12.2 ± 5.5, 8.4 ± 4.2, 288 ± 73) μmol/L) ($P<0.05 - 0.01$). However, no difference existed when comparing different grades of MG patients according to the modified Osserman classification. Also no significant difference existed between MG patients with thymic abnormalities and those without. In comparison with the

DOI:10.3760/cma.j.issn.0376-2491.2013.17.003

作者单位:230022 合肥,安徽医科大学第一附属医院神经内科

通信作者:孙中武,Email:sunzhwu@hotmail.com

subjects in the reference group (total bilirubin≥11.4 μmol/L, UA≥279 μmol/L), the odds ratio (95% *CI*) for MG patients in the lower tertile (total bilirubin <11.4 μmol/L, UA <279 μmol/L) were 1.98 (1.20 - 3.29) and 2.22 (1.29 - 3.82) respectively after multivariable adjustment. Serum level of TBIL and UA had positive correlations with creatintine as indicated by Pearson correlation analysis (r = 0.151, P = 0.008; r = 0.301, P = 0.000). Meanwhile, serum level of UA had a negative correlation with high density lipoprotein-cholesterol (r = -0.347, P = 0.000). **Conclusion** Decreased serum levels of BIL and UA are closely correlated with MG. As a replacement therapy, administration of BIL and UA or theirs precursors may offer benefits to the MG patients.

【**Key words**】 Bilirubin; Uric acid; Mysthenia gravis

重症肌无力(myasthenia gravis,MG)是一种以神经-肌肉接头处传递障碍为特征的自身免疫性疾病。胆红素(bilirubin,BIL)长期以来一直被视为是机体的代谢废物,但近年来的研究发现胆红素具有包括抗炎、抗氧化、免疫调节、细胞及神经保护等多种生物学活性[1-2]。尿酸(uric acid,UA)是嘌呤的代谢终产物,同时也是一种强效的氧自由基和氮自由基清除剂,尤其对过氧亚硝酸盐的清除[3]。研究表明,胆红素和尿酸与多发性硬化(MS)[4]、冠心病及脑卒中等[5-6]多种疾病的发生密切相关,目前对于二者在 MG 中的变化研究较少。本研究通过对 MG 患者血清胆红素及尿酸变化进行观察,旨在探讨血清胆红素和尿酸在 MG 中的临床意义。

对象与方法

一、对象

MG 组选自 2010 年 1 月至 2012 年 12 月在安徽医科大学第一附属医院神经内科住院的 MG 患者,共 131 例,其中男 68 例,女 63 例,年龄为 7 ~84 岁,平均(45 ±19)岁。正常对照组 176 名为同期健康体检者,其中男 96 名,女 80 名,年龄为 8 ~73 岁,平均(46 ±15)岁。本研究受试对象均知情同意且经安徽医科大学伦理委员会批准。

MG 诊断标准及分型:MG 患者的诊断依据临床表现、新斯的明试验及实验室检查结果等多方面确诊[7],同时按照 Osserman 分型标准分组:Ⅰ型为单纯眼肌型;Ⅱa 为轻度全身型,累及面肌及四肢肌肉,无明显咽喉肌受累;Ⅱb 型为中度全身型,严重肌无力伴延髓肌受累;Ⅲ型为急性进展型,首次症状出现数周内即发展至延髓肌,肢带肌、躯干肌肉和呼吸肌肉严重无力,伴重症肌无力危象;Ⅳ型为晚发全身型,由Ⅰ、Ⅱ型发展而来,症状同Ⅲ型。Ⅴ型肌萎缩型由于罕见(仅 1 例)未纳入研究组。

排除标准:(1)排除各种原因所致的肝功能损害(ALT >40 U/L,AST >40 U/L)、溶血性黄疸、阻塞性黄疸、感染性疾病、严重的肾脏疾病、自身免疫性疾病、代谢综合征和痛风患者等;(2)在测定胆红素和尿酸之前使用过甾体类等影响胆红素和尿酸代谢的药物;(3)排除可能为 Gilbert 综合征者(血清总胆红素 >34 μmol/L)。

二、方法

1. 病史收集:于入院当天收集所有患者详细的病史资料,包括身高、体重、吸烟(每天吸卷烟 1 支以上,连续或累计 6 个月)、肝肾疾病病史等,其中身高以(cm)为单位,体重以(kg)为单位,并计算体质量指数[BMI = 体质量(kg)/身高(m)^2]。

2. 生化指标测定:入院 24 h 内,采集所有患者空腹 >12 h 的静脉血。采用德国拜耳 ADVIA 1650 全自动分析仪测定血清直接胆红素(DBIL)、间接胆红素(IBIL)、总胆红素(TBIL)、ALT 及 AST 等肝功能指标,尿酸、肌酐(Cr)及尿素氮(BUN)等肾功能指标和 HDL-C、LDL-C 等血脂相关指标。

3. 胸腺 CT:所有患者入院后均行胸腺 CT 检查确定有无伴随胸腺增生/胸腺瘤。

三、统计学处理

采用 SPSS 16.0 软件进行统计学分析,计量资料以 $\bar{x} \pm s$ 表示,MG 组与正常对照组间比较采用 t 检验,MG 不同分型患者各指标采用方差分析,计数资料比较采用 χ^2 检验,相关分析采用 Pearson 分析及多元 Logistic 回归分析。以 $P \leq 0.05$ 为差异有统计学意义。

结 果

1. MG 患者与正常对照组间一般资料比较:MG 组与正常对照两组年龄、性别、吸烟、Cr 及 BUN 间差异均无统计学意义,而 MG 组 BMI、HDL-C 及 LDL-C 显著低于正常对照组,差异有统计学意义(P <0.05 ~0.01,表 1)。

2. 两组间血清 DBIL、IBIL、TBIL、尿酸水平比较:MG 组的 IBIL、TBIL、尿酸均显著低于正常对照组(P <0.01),DBIL 略高于正常对照组,差异无统计学意义。将两组按不同性别分别比较时,这种差

表 1　MG 与正常对照两组间基本临床资料比较($\bar{x} \pm s$)

组别	例数	男性(%)	年龄(岁)	BMI(kg/m^2)	吸烟(%)	BUN(μmol/L)	Cr(μmol/L)	HDL-C(μmol/L)	LDL-C(μmol/L)
MG 组	131	68(54.5)	45 ± 19	21 ± 6	34(26.0)	5.4 ± 2.0	57 ± 18	1.5 ± 0.4	2.6 ± 0.8
健康对照组	176	96(51.9)	46 ± 15	24 ± 3	47(26.7)	5.1 ± 1.7	59 ± 15	1.6 ± 0.5	3.0 ± 1.1
P 值		0.366	0.636	0.001	0.495	0.121	0.280	0.023	0.001

异仍然存在,以 IBIL 和尿酸更为显著。MG 组组内不同性别各指标比较时发现,男性 DBIL、IBIL、TBIL 及尿酸水平均高于女性,其中 IBIL、TBIL 及尿酸间差异有统计学意义($P < 0.05 \sim 0.01$,表 2)。

3. MG 组不同分型间 DBIL、IBIL、TBIL 及尿酸水平比较:MG 组不同分型间 DBIL、IBIL、TBIL 及尿酸水平差异均无统计学意义(*P* 分别为 0.124,0.516,0.218,0.507,表 3)。

表 2　MG 与正常对照组间血清胆红素和尿酸水平比较($\bar{x} \pm s$, μmol/L)

组别	例数	DBIL	IBIL	TBIL	UA
MG 组	131	3.6 ± 0.1	7.4 ± 0.3^{a}	11.0 ± 0.4^{a}	270 ± 70^{a}
男	68	3.8 ± 1.7	8.4 ± 4.2^{ac}	12.2 ± 5.5^{bd}	288 ± 73^{ac}
女	63	3.4 ± 1.4	6.7 ± 2.7^{a}	10.1 ± 3.7^{a}	242 ± 68^{b}
健康对照组	176	3.3 ± 0.1	9.6 ± 0.3	13.0 ± 0.4	301 ± 60
男	96	3.5 ± 1.2	9.8 ± 3.9	13.3 ± 5.0	340 ± 95
女	80	3.1 ± 1.1	9.1 ± 3.1	12.2 ± 4.0	263 ± 57

注:与对照组相比$^{a}P < 0.01$,$^{b}P < 0.05$;MG 组内男女间比较$^{c}P < 0.01$,$^{d}P < 0.05$

表 3　MG 不同分型患者血清胆红素和尿酸水平比较($\bar{x} \pm s$, μmol/L)

MG 分型	例数	DBIL	IBIL	TBIL	UA
Ⅰ型	45	3.3 ± 1.2	7.5 ± 4.0	10.8 ± 4.8	274 ± 71
Ⅱa 型	28	3.6 ± 1.7	7.6 ± 3.8	11.2 ± 5.1	262 ± 75
Ⅱb 型	35	4.0 ± 1.8	7.6 ± 3.8	11.6 ± 4.9	276 ± 81
Ⅲ型	15	3.6 ± 1.6	7.8 ± 3.4	11.4 ± 4.6	245 ± 51
Ⅳ型	8	3.4 ± 1.3	8.1 ± 3.9	11.4 ± 5.1	228 ± 85

4. MG 组中有无胸腺异常患者间各指标水平比较:MG 组中伴有胸腺增生/胸腺瘤患者 22 例,占 16.8%,与无胸腺增生/胸腺瘤患者相比,DBIL、IBIL、TBIL 及尿酸水平均有所降低,但差异无统计学意义(*P* 分别为 0.06,0.821,0.439,0.402)。

5. 多元逐步 Logistic 回归分析:在校正年龄、性别、吸烟、BMI、BUN、Cr、HDL-C 及 LDL-C 等相关因素后发现,低胆红素水平者(TBIL < 11.4 μmol/L)患 MG 的风险是高胆红素水平的 1.98 倍;低尿酸水平者(UA < 279 μmol/L)患 MG 的风险是高尿酸水平的 2.22 倍(表 4)。

6. 血清胆红素和尿酸与其他指标间相关关系分析:TBIL 与 Cr($r = 0.151$, $P = 0.008$)呈正相关关系;尿酸与 Cr($r = 0.301$, $P = 0.000$)间呈正相关,而与 HDL-C($r = -0.347$, $P = 0.000$)间存在负相关关系。

表 4　多元逐步 Logistic 回归分析

变量	偏回归系数	*Wald*	*P* 值	*OR*	95% *CI*
BUN	0.180	5.859	0.015	1.197	1.035 ~ 1.385
HDL-C	−1.073	9.777	0.002	0.342	0.174 ~ 0.670
LDL-C	−0.419	7.580	0.006	0.657	0.488 ~ 0.886
BMI	−1.141	14.684	0.000	0.868	0.808 ~ 0.933
UA	0.797	8.288	0.004	2.218	1.290 ~ 3.816
TBIL	0.684	7.020	0.008	1.981	1.195 ~ 3.285

讨　论

近年来有研究发现 MG 的患病率有逐年上升趋势[8],其病理机制基本明确,为各种原因导致的运动终板突触后膜上乙酰胆碱受体(AChR)的减少,其中免疫机制在这一病理生理过程中的作用较为肯定,但也有研究认为氧化损伤可能参与了自身免疫疾病的发生[9]。活性氧簇(ROS)和活性氮簇(RNS)是机体在对体内外因素氧化生物反应过程中产生的一类氮氧化物或氮氧自由基。ROS 和 RNS 的过度产生或抗氧化物质的减少均会导致机体氧化失衡,造成氧化损伤及细胞凋亡[10]。2005 年,Venkatesham 等[11]首次报道了 ROS 对 AChR 的损伤作用,认为抗氧化物质在防止自由基介导的受体损伤中发挥了重要作用。最近,Krishnaswamy 等[12]进一步阐释了 ROS 对 AChR 的损伤机制,认为可能与 ROS 导致受体残存半胱氨酸过多有关。Stuerenburg[13]也肯定了抗氧化治疗在神经肌肉疾病中的作用。有研究显示胆红素和尿酸并不仅仅是机体的代谢废物,生理浓度的胆红素抗氧化能力比其他抗氧化物质强数十倍,如维生素 E 等,而尿酸在机体清除自由基过程中占 60%。另外,胆红素由于其具有强大的亲脂活性而与细胞膜相连[1],可通过各种途径发挥细胞保护及免疫调节作用[3]。为此,我们认为 MG 患者血清胆红素和尿酸的变化及

其机制值得探讨。

我们研究发现 MG 患者的血清 IBIL 及 TBIL 水平显著低于正常对照组,将其按不同性别分别比较时这种差异仍然存在,此与 Fuhua 等[14]的研究结果基本一致。但与其结果有所不同的是,本组研究中 MG 组患者与正常对照组间 DBIL 差异并不显著,这可能是由于只有亲脂性的物质(如 TBIL 和 IBIL)才能够较容易的被动进入细胞膜发挥细胞保护、免疫传递及调节功能[15],而亲水性的 DBIL 作用可能并不突出。我们对 MG 组组内不同性别患者进行比较时发现女性患者血清胆红素水平显著低于男性,这与既往的研究结果一致[14]。

除血清胆红素改变外,我们还发现 MG 患者的血清尿酸水平亦显著低于正常对照组。为排除肾功能异常对尿酸排出所造成的影响,我们比较了受试者 Cr 和 BUN 等肾功能指标,结果 MG 组与对照组间无明显差异。考虑到性别因素对于血清尿酸的影响,我们将 MG 组和正常对照组按不同性别分别比较时也得到了类似结果。同样,MG 组组内男女患者间的尿酸水平间也存在差异,女性患者的尿酸水平较男性显著降低。目前对于这种不同性别间血清胆红素和尿酸水平差异的机制尚不十分清楚,有学者[16]认为这可能是由于男女饮食结构、生活方式不同导致体内具有保护性作用的雌激素水平不一所致,我们推测这种低水平的血清尿酸和胆红素可能与 MG 女性患病率高之间具有一定相关性,此有待将来进一步大样本研究证实。

为进一步探究 MG 患者组织受累范围与血清胆红素和尿酸改变之间的关系,我们将 MG 患者进行分型,结果发现不同类型 MG 患者血清胆红素和尿酸水平并无显著差异,这种与机体损伤部位或程度不相匹配血清胆红素和尿酸的变化同样存在于其他疾病中,如多发性硬化[17]。对于这一结果目前尚无合理阐释。而将伴有胸腺增生/胸腺瘤患者与无伴随改变者进行比较时发现,伴有胸腺异常改变者血清胆红素及尿酸均较低,但这种差异无统计学意义,可能与本研究观察样本量较少有关。为了排除年龄、性别、吸烟、BMI、BUN、Cr、HDL-C 及 LDL-C 等因素的影响,我们进行多元逐步 Logistic 回归分析发现,在校正相关因素后,低水平胆红素者患 MG 的风险是高水平的 1.98 倍,低尿酸水平者患 MG 风险则增加 2.22 倍,可见胆红素和尿酸在 MG 中具有一定的保护作用。此外,相关分析还发现 TBIL 与 Cr 间存在着一定的正相关关系,提示血浆 TBIL 水平除了与机体本身的氧化水平有关外,与肾脏排泄也密切相关;尿酸与 Cr 之间也存在一种正相关关系,而与 HDL-C 为负相关关系。在人类的生理节律中已经确定尿酸与 NO 呈负相关,而 HDL-C 能够恢复内皮细胞中被消弱了的 NO 合成[18],从而间接使得尿酸水平降低,提示机体内各指标间能够相互作用、相互影响。

通过以上研究,我们可以发现 MG 患者血清胆红素和尿酸水平较正常对照均显著降低,但对于血清胆红素和尿酸的这种变化与 MG 间的因果关系目前仍不明确。一方面 MG 的发生可能是由于患者血清中胆红素和尿酸基础量不足而导致 ROS 作用占优势,造成机体的氧化损伤及免疫调节失衡,从而导致 MG 发生;另一方面也可能由于 MG 病理状况下机体过度产生的 ROS 消耗了血清胆红素和尿酸,导致血清胆红素和尿酸水平下降。在对 MS 的研究中,有学者通过测定尿酸的代谢产物尿囊素,发现 MS 患者血清或脑脊液中尿囊素并未增加而更倾向于第一种解释[19],但这与 MG 中的改变是否一致,仍待将来进一步研究证实。

胆红素和尿酸由于具有强大的抗氧化活性和一定程度的免疫调节功能,其水平的降低可能与 MG 的发生有关。胆红素和尿酸的保护性作用同样体现在其他的自身免疫性疾病,如 MS、视神经脊髓炎(NMO)中,因此通过补充外源性胆红素及尿酸是否可预防此类疾病发生是今后的研究方向[4],这也为我们治疗 MG 提供了新的思路。

参 考 文 献

[1] Stocker R, Yamamoto Y, McDonagh AF, et al. Bilirubin is an antioxidant of possible physiological importance. Science, 1987, 235:1043-1046.

[2] Liu Y, Li P, Lu J, et al. Bilirubin possesses powerful immunomodulatory activity and suppresses experimental autoimmune encephalomyelitis. J Immunol, 2008, 181: 1887-1897.

[3] Hooper DC, Spitsin S, Kean RB, et al. Uric acid, a natural scavenger of peroxynitrite, in experimental allergic encephalomyelitis and multiple sclerosis. Proc Natl Acad Sci USA, 1998, 95: 675-680.

[4] Liu Y, Liu J, Tetzlaff W, et al. Biliverdin reductase, a major physiologic cytoprotectant, suppresses experimental autoimmune encephalomyelitis. Free Radic Biol Med, 2006, 40: 960-967.

[5] Breimer LH, Mikhailidis DP. Is bilirubin a marker of vascular disease and/or cancer and is it a potential therapeutic target? . Curr Pharm Des, 2011, 17: 3644-3655.

[6] 何平, 谢贤和, 丁毅鹏, 等. 高敏 C 反应蛋白、脂蛋白 a、血尿酸与冠状动脉病变程度的相关性. 中华医学杂志, 2010, 90: 1989-1991.

[7] Spillane J, Higham E, Kullmann DM. Myasthenia gravis. BMJ, 2012, 345: e8497.

[8] Lavrnic D, Basta I, Rakocevic-Stojanovic V, et al. Epidemiological Study of Adult-Onset Myasthenia Gravis in the Area of Belgrade (Serbia) in the Period 1979-2008. Neuroepidemiology, 2013, 40:190-194.
[9] Brambilla D, Mancuso C, Scuderi MR, et al. The role of antioxidant supplement in immune system, neoplastic, and neurodegenerative disorders: a point of view for an assessment of the risk/benefit profile. Nutr J, 2008, 7:29.
[10] Kurien BT, Scofield RH. Autoimmunity and oxidatively modified. Autoimmun Rev, 2008, 7: 567-573.
[11] Venkatesham A, Babu PS, Sagar JV, et al. Effect of reactive oxygen species on cholinergic receptor function. J India Pharmacol, 2005, 37: 366-370.
[12] Krishnaswamy A, Cooper E. Reactive oxygen species inactivate neuronal nicotinic acetylcholine receptors through a highly conserved cysteine near the intracellular mouth of the channel: implications for diseases that involve oxidative stress. J Physiol, 2012, 590(Pt 1):39-47.
[13] Stuerenburg HJ. The roles of carnosine in aging of skeletal muscle and in neuromuscular diseases. Biochemistry (Mosc), 2000, 65: 862-865.
[14] Fuhua P, Xuhui D, Zhiyang Z, et al. Antioxidant status of bilirubin and uric acid in patients with myasthenia gravis. Neuroimmunomodulation, 2012, 19: 43-49.
[15] Wu TW, Fung KP, Wu J, et al. Antioxidation of human low density lipoprotein by unconjugated and conjugated bilirubins. Biochem Pharmacol, 1996, 51: 859-862.
[16] Sullivan JL. Iron and the sex difference in heart disease risk. Lancet, 1981, 1:1293 e4.
[17] Liu B, Shen Y, Xiao K, et al. Serum uric acid levels in patients with multiple sclerosis: a meta-analysis. Neurol Res, 2012, 34: 163-171.
[18] Onat A, Can G, Yüksel H. Dysfunction of high-density lipoprotein and its apolipoproteins: new mechanisms underlying cardiometabolic risk in the population at large. Turk Kardiyol Dern Ars, 2012, 40:368-385.
[19] Kastenbauer S, Kieseier BC, Becker BF. No evidence of increased oxidative degradation of urate to allantoin in the CSF and serum of patients with multiple sclerosis. J Neurol, 2005, 252: 611-612.

（收稿日期:2013-03-01）
（本文编辑:朱瑶）

·读者·作者·编者·

本刊对来稿中统计学处理的有关要求

1. 统计研究设计:应交代统计研究设计的名称和主要做法。如调查设计(分为前瞻性、回顾性或横断面调查研究);实验设计(应交代具体的设计类型,如自身配对设计、成组设计、交叉设计、析因设计、正交设计等);临床试验设计(应交代属于第几期临床试验,采用了何种盲法措施等)。主要做法应围绕 4 个基本原则(随机、对照、重复、均衡)概要说明,尤其要交代如何控制重要非试验因素的干扰和影响。

2. 资料的表达与描述:用 $\bar{x} \pm s$ 表达近似服从正态分布的定量资料,用 $M(Q_R)$ 表达呈偏态分布的定量资料;用统计表时,要合理安排纵横标目,并将数据的含义表达清楚;用统计图时,所用统计图的类型应与资料性质相匹配,并使数轴上刻度值的标法符合数学原则;用相对数时,分母不宜小于 20,要注意区分百分率与百分比。

3. 统计分析方法的选择:对于定量资料,应根据所采用的设计类型、资料所具备的条件和分析目的,选用合适的统计分析方法,不应盲目套用 t 检验和单因素方差分析;对于定性资料,应根据所采用的设计类型、定性变量的性质和频数所具备的条件以及分析目的,选用合适的统计分析方法,不应盲目套用 χ^2 检验。对于回归分析,应结合专业知识和散布图,选用合适的回归类型,不应盲目套用简单直线回归分析,对具有重复实验数据的回归分析资料,不应简单化处理;对于多因素、多指标资料,要在一元分析的基础上,尽可能运用多元统计分析方法,以便对因素之间的交互作用和多指标之间的内在联系进行全面、合理的解释和评价。

4. 统计结果的解释和表达:当 $P < 0.05$(或 $P < 0.01$)时,应说明对比组之间的差异有统计学意义,而不应说对比组之间具有显著性(或非常显著性)的差别;应写明所用统计分析方法的具体名称(如:成组设计资料的 t 检验、两因素析因设计资料的方差分析、多个均数之间两两比较的 q 检验等),统计量的具体值(如 $t = 3.45$, $\chi^2 = 4.68$, $F = 6.79$ 等)应尽可能给出具体的 P 值(如 $P = 0.023$);当涉及到总体参数(如总体均数、总体率等)时,在给出显著性检验结果的同时,再给出 95% 可信区间。

DOI:10.16766/j.cnki.issn.1674-4152.2015.06.029

·全科临床研究·

血清胆红素和尿素水平表达对重症肌无力患者的研究价值

黄伟玲

摘要: 目的 探究血清胆红素(BIL)和尿素(UA)水平变化对于重症肌无力(MG)患者的临床价值。**方法** 选取嵊州市人民医院 2008 年 1 月—2013 年 12 月重症肌无力患者 20 例为试验组,同期体检健康正常人群 20 名为对照组。采用氧化酶定量分析法测定血清 BIL 和 UA 值,观察 2 组患者直接胆红素(DBIL)、间接胆红素(IBIL)、总胆红素(TBIL)和 UA 值之间的差异。分别对试验组不同性别和有无伴随胸腺异常 MG 患者的各项指标进行比较并进行相关分析。**结果** 对试验组和对照组患者的血清 DBIL、IBIL、TBIL、UA 分析结果显示,试验组的 IBIL、TBIL、UA 均明显低于对照组,差异有统计学意义($P=0.041$、0.027、0.029)。按性别对 2 组进行组内比较结果显示,对照组女性 UA 水平明显低于男性,差异有统计学意义($P=0.037$);试验组女性患者 IBIL、TBIL 及 UA 水平均低于男性,差异有统计学意义($P=0.011$、0.031、0.017)。试验组中有无胸腺异常患者 BIL 和 UA 水平比较结果显示,有无伴随胸腺异常患者的血清 DBIL、IBIL、TBIL、UA 水平之间差异无统计学意义($P>0.05$)。Logistic 分析:低胆红素水平者患 MG 的风险是高胆红素水平的 2.01 倍,低尿酸水平者患 MG 的风险是高尿酸水平的 2.26 倍。**结论** 血清 BIL 和 UA 水平与 MG 密切相关,具有一定临床诊断价值。

关键词: 重症肌无力;血清胆红素;尿酸;临床价值

中图分类号: R746.1 **文献标识码:** A **文章编号:**1674-4152(2015)06-0939-03

Clinical value of changes of serum bil and ua levels in patients with myasthenia gravis *HUANG Wei-ling. Department of Electromyography,the People's Hospital of Shengzhou,Shengzhou 312400,Zhejiang,China*

Abstract:Objective To explore the clinical value of changes of serum BIL and UA levels in patients with myasthenia gravis(MG). **Methods** A total of 20 MG patients,which were enrolled from my hospital between January 2008 and December 2013 were selected as test group,and 20 healthy cases as control group. The total serum BIL and UA concentrations were measured by quantitative analysis oxidase. The differences of DBIL, IBIL,TBIL and UA between the two groups were observed. The test group was grouped by gender,and thymic abnormalities. Indicators were compared and analyzed. **Results** The IBIL,TBIL,UA in patients of test group were significantly lower than those in control group($P=0.041$, 0.027,0.029). In control group,the UA in females was significantly lower than that in males($P=0.037$). In test group, the IBIL,TBIL and UA in females were significantly lower than that in males($P=0.011$,0.031,0.017). No significant difference existed between patients with thymic abnormalities and those without. The risk of suffering from MG of patients with low levels of bilirubin was 2.01 times as much as high levels of bilirubin,and the risk of suffering from MG of patients with low levels of uric acid was 2.26 times as much as high levels of uric acid. **Conclusion** The serum BIL and UA in MG patients were significantly lower than those in normal population. It can be an important basis on prevention and treatment of MG,which had great value in clinical research and application.

Key words: Myasthenia gravis;Serum bilirubin;Uric acid;Clinical value

重症肌无力(myasthenia gravis,MG)是一种以乙酰胆碱传递障碍为特征的自身免疫性疾病[1]。初期发病隐匿,多数患者未及时寻求有效医治,导致肌无力危象等危重症状。因此如何及早防治 MG 是预防和延缓患者病情加重的关键[2]。近年研究发现抗氧化物质在自身免疫性疾病的防治中发挥重要作用[3],其中胆红素(bilirubin,BIL)和尿酸(uric acid,UA)对多发性硬化的预防和保护作用[4-5]。因此,为更加深入的了解 MG 并研究更为有效的预防措施,通过对我院神经内科 MG 患者的血清胆红素和尿素水平进行分析,以期为 MG 早期诊断提供一定指导。

1 资料与方法

1.1 临床资料 选取 2008 年 1 月—2013 年 12 月在我院神经内科住院的重症肌无力患者 20 例作为试验组,其中男性 9 例,女性 11 例,年龄为 3~67 岁,平均年龄(31.25±20.17)岁。所有试验组研究对象均经临床表现、新斯的明试验、肌电图等检查确诊为重症肌无力(MG)[6]。排除标准:①排除患有各种肝肾功能损害的患者;②排除患有自身免疫性疾病、代谢综合症、Gilbert 综合征或痛风等患者;③排除试验前使用过甾体类等影响胆红素和尿素代谢的药物的患者。选择同期健康体检者 20 名作为对照组,其中男性 8 例,女性 12 例,年龄 4~69 岁,平均年龄(32.60±19.54)岁。本研究经医院伦理委员会批准,所有研究对象均签署知情同意书。

1.2 研究方法

1.2.1 病史收集 收集试验组和对照组患者的病史资料,并计算患者的体质量指数 BMI=体质量(kg)/身高^2(m^2)。

作者单位:312400 浙江省嵊州市人民医院肌电图室

(C)1994-2024 China Academic Journal Electronic Publishing House. All rights reserved. http://www.cnki.net

1.2.2 BIL 和 UA 水平检测 采集 2 组研究对象空腹 12 h 以上的静脉血。采用德国拜耳 ADVIA 1650 全自动分析仪测定肝功能指标：血清直接胆红素（DBIL）、间接胆红素（IBIL）、总胆红素（TBIL）、谷草转氨酶（AST）、谷丙转氨酶（ALT）、肾功能指标：尿酸（UA）、肌酐（Cr）、尿素氮（BUN）等和血脂相关指标：HDL-C、LDL-C 等。

1.2.3 其他检查 胸腺 CT：2 组受试者均进行胸腺 CT 检查确定是否伴随胸腺增生或胸腺瘤。

1.3 统计学方法 采用 SPSS 17.0 软件进行统计学数据分析，计量资料采用 t 检验以（$\bar{x} \pm s$）表示，计数资料比较采用 χ^2 检验，相关分析采用多元 Logistic 回归分析。$P<0.05$ 为差异有统计学意义。

2 结 果

2.1 病史资料比较结果 对试验组和对照组的一般资料进行比较，2 组受试者的年龄、性别、吸烟、肾功能指标（Cr、BUN）、血脂相关指标（HDL-C、LDL-C）间差异无统计学意义（$P>0.05$），见表 1。

表 1 2 组间人员一般资料比较

组别	例数	男性（%）	平均年龄（岁）	BMI（kg/m^2）	吸烟（%）	BUN（mmol/L）	Cr（μmol/L）	HDL-C（mmol/L）	LDL-C（mmol/L）
试验组	20	9(45)	31 ±20	20 ±7	6(30)	5.5 ±1.9	59 ±15	1.2 ±0.5	2.5 ±1.2
对照组	20	8(40)	33 ±19	24 ±4	5(25)	5.2 ±1.3	57 ±17	1.6 ±0.3	2.7 ±1.6
P 值		0.349	0.177	0.271	0.189	0.435	0.925	0.817	0.934

2.2 血清 BIL 和 UA 水平检测结果

2.2.1 试验组和对照组血清 DBIL、IBIL、TBIL、UA 比较 试验组的 IBIL、TBIL、UA 均明显低于对照组，差异有统计学意义（$P=0.041, 0.027, 0.029$），DBIL 略高于对照组，差异无统计学意义（$P=0.161$），见表 2。

表 2 2 组间 BIL 和 UA 水平比较（μmol/L）

组别	例数	DBIL	IBIL	TBIL	UA
试验组	20	3.7 ±0.6	6.5 ±0.2	10.9 ±0.5	268 ±72
对照组	20	3.2 ±0.1	9.8 ±0.4	14.1 ±0.5	310 ±69
P 值		0.161	0.041	0.027	0.029

2.2.2 按性别对 2 组进行组内比较 对照组不同性别间仍存在差异，其中 UA 水平间差异有统计学意义（$P=0.037$），见表 3；试验组组内不同性别各指标间比较发现，男性患者 DBIL、IBIL、TBIL 及 UA 水平均高于女性，其中 IBIL、TBIL、UA 间差异有统计学意义（$P=0.011$、0.031、0.017），见表 4。

表 3 对照组不同性别间 BIL 和 UA 水平比较（μmol/L）

性别	例数	DBIL	IBIL	TBIL	UA
男	8	3.3 ±1.0	9.9 ±0.7	15.6 ±4.1	330 ±70
女	12	3.1 ±1.2	9.6 ±0.3	13.3 ±5.7	280 ±88
P 值		0.634	0.731	0.511	0.037

表 4 试验组不同性别间 BIL 和 UA 水平比较（μmol/L）

性别	例数	DBIL	IBIL	TBIL	UA
男	9	3.9 ±1.0	8.7 ±0.6	11.8 ±3.1	292 ±82
女	11	3.4 ±1.5	5.9 ±1.4	9.9 ±6.9	244 ±73
P 值		0.064	0.011	0.031	0.017

2.2.3 试验组中有无胸腺异常患者 BIL 和 UA 水平比较 其中伴有胸腺异常（胸腺增生/胸腺瘤）的患者 DBIL、IBIL、TBIL、UA 水平均低于无胸腺异常者，但差异无统计学意义（$P>0.05$），见表 5。

2.2.4 Logistic 分析 在校正病史资料等相关因素后发现，低胆红素水平者患 MG 的风险是高胆红素水平的 2.01 倍，低尿酸水平者患 MG 的风险是高尿酸水平的 2.26 倍，见表 6。

表 5 试验组有无胸腺异常患者间 BIL 和 UA 水平比较（μmol/L）

有无胸腺异常	例数	DBIL	IBIL	TBIL	UA
有	5	3.6 ±1.2	6.3 ±1.3	9.7 ±0.9	259 ±83
无	15	3.8 ±0.7	6.8 ±2.2	11.1 ±1.7	271 ±76
P 值		0.194	0.201	0.097	0.328

表 6 多元逐步 Logistic 回归分析

变量	β	*Wald* 值	*OR*	95% *CI*	P 值
BUN	0.162	5.337	1.176	1.031 ~1.476	0.019
HDL-C	−1.103	9.214	0.332	0.103 ~0.651	0.002
LDL-C	−0.435	7.521	0.647	0.201 ~0.765	0.007
BMI	−0.209	14.787	0.811	0.687 ~0.985	0.000
UA	0.816	8.264	2.261	1.602 ~2.262	0.004
TBIL	0.750	6.977	2.017	1.158 ~2.161	0.008

3 讨 论

2 组的血清 DBIL、IBIL、TBIL、UA 比较，试验组的 IBIL、TBIL、UA 均明显低于对照组，提示 IBIL、TBIL、UA 对 MG 诊断有一定价值。一方面，低于正常水平的 BIL 和 UA 无法抵抗体内 ROS 和 RNS 等的氧化作用，引发机体免疫失衡，导致 MG 的发生[7-8]。另一方面，MG 患者机体需要消耗大量的 BIL 和 UA 以对抗 ROS 和 RNS 等的氧化作用，进而引起血清 BIL 和 UA 水平降低[9-10]。而 2 组 DBIL 差异无统计学意义，这可能是由于 TBIL 和 IBIL 为亲脂性物质，可通过单纯扩散等被动运输方式直接进入细胞膜发挥抗氧化作用，保护细胞免受自身免疫损伤，而 DBIL 为亲水性，膜内作用不明显[11]。

血清 UA 水平结果试验组低于对照组，结合病史资料比较 2 组患者的 Cr 和 BUN，未见明显差异，可排除肾功异常对尿酸水平的影响。按性别分别对 2 组患者进行组内比较，对照组不同性别间仍存在差异，其中 UA 水平间差异有统计学意义；试验组组内不同性别各指标间比较发现，男性患者 IBIL、TBIL 及 UA 水平均高于女性。研究结果表明试验组和对照组的女性血清 UA 水平均明显低于男性，但女性与男 （下转第 943 页）

(C)1994-2024 China Academic Journal Electronic Publishing House. All rights reserved. http://www.cnki.net

种参与凝血过程的重要中间产物。

综上所述,血小板参数和凝血指标与肝硬化患者的 Child-Pugh 分级严重程度密切相关,对估计患者病情具有重要意义。

参考文献

[1] 李玉才. 肝硬化患者血小板参数和凝血指标变化及与肝功能 Child-Pugh 分级的关系[J]. 山东医药,2010,50(35):45-46.

[2] Hong W,Yang N,He J,et al. The dynamic changes of 4 platelet parameters and coagulation factor in bleeding patients with liver cirrhosis[J]. Chinese Journal of Health Laboratory Technology,2011,9:53.

[3] 辛晓丽. 肝硬化早期腹水相关因素分析[J]. 中国现代医生,2014,8(12):11-12,16.

[4] Takaya H,Uemura M,Fujimura Y,et al. ADAMTS13 activity may predict the cumulative survival of patients with liver cirrhosis in comparison with the Child-Turcotte-Pugh score and the Model for End-Stage Liver Disease score[J]. Hepatology Research,2012,42(5):459-472.

[5] Sansoe G,Aragno M,Mastrocola R,et al. 541 Chymase-Dependent Production of Kidney Angiotensin II is a Key Factor in the Development of Sodium Retention and Ascites in Experimental Liver Cirrhosis[J]. Gastroenterology,2012,142(5):S-918.

[6] 王珏琼. 血小板、凝血指标与老年肝硬化患者 Child-Pugh 分级的关系[J]. 中国老年学杂志,2013,33(24):6103-6105.

[7] 李红艳,斯庆图娜拉,张永贵,等. 老年肝硬化患者凝血及血小板功能变化与 Child-Pugh 分级的关系[J]. 中国老年学杂志,2011,31(6):951-952.

[8] Zimmermann H W,Koch A,Seidler S,et al. Circulating soluble urokinase plasminogen activator is elevated in patients with chronic liver disease,discriminates stage and aetiology of cirrhosis and predicts prognosis[J]. Liver International,2012,32(3):500-509.

[9] Xianghong G,Guanping C,Fenghua Y,et al. Changes in platelet functional parameters and CD62 P expression in liver cirrhosis[J]. African Health Sciences,2014,13(4):1079-1083.

[10] 吕胜,张伟. 肝硬化合并胆囊结石的相关因素研究[J]. 中国现代医生,2014,8(14):1-3,7.

[11] 占国清,谭华炳,李儒贵,等. 血小板参数和凝血指标的变化与肝硬化 Child-Pugh 分级的关系[J]. 临床消化病杂志,2012,24(1):11-13,19.

[12] Chen H,Qi X,He C,et al. Coagulation imbalance may not contribute to the development of portal vein thrombosis in patients with cirrhosis[J]. Thrombosis research,2013,131(2):173-177.

[13] Imai K,Takai K,Hanai T,et al. Impact of Serum Chemerin Levels on Liver Functional Reserves and Platelet Counts in Patients with Hepatocellular Carcinoma[J]. International journal of molecular sciences,2014,15(7):11294-11306.

[14] 占国清,谭华炳,李儒贵,等. 血清前白蛋白、总胆汁酸、凝血指标、血小板参数检测在肝硬化中的临床意义[J]. 中国肝脏病杂志(电子版),2014,7(1):59-62.

[15] 乔俊妮. 老年肝硬化患者凝血指标及血小板参数与肝硬化 Child-Pugh 分级的相关性研究[J]. 实用检验医师杂志,2013,5(4):202-204.

[16] Potze W,Arshad F,Adelmeijer J,et al. Routine coagulation assays underestimate levels of antithrombin-dependent drugs but not of direct anticoagulant drugs in plasma from patients with cirrhosis[J]. British journal of haematology,2013,163(5):666-673.

收稿日期:2014-10-25

(上接第940页)

性间的血清 UA 值即存在一定差异,目前尚无有力依据对此现象进行解释。一种观点认为这可能与男女之间的饮食习惯、生活方式不同有关;另一种观点认为女性体内具有保护作用的雌激素水平较高,使女性的机体代谢与男性之间存在差异[12]。因此,我们推测试验组较低的 UA 水平可能与 MG 女性发病率较高存在一定相关性,但这一推论需要进一步的验证。

为了排除年龄、性别、吸烟情况、血脂指标、肾功能等因素对研究结果的影响,采用多元逐步分析法对相关因素进行校正后,我们发现低胆红素水平者患 MG 的风险是高胆红素水平的 2.01 倍,低尿酸水平者患 MG 的风险是高尿酸水平的 2.26 倍,提示血清 BIL 和 UA 在 MG 中存在一定的保护作用。

本研究发现 MG 患者的血清 BIL 和 UA 水平较低。因此我们可以大胆推测 MG 的防治新手段:可以通过监测血清 BIL 和 UA 水平,对 MG 进行初期筛查,还可以通过补充外源性 BIL 和 UA 对 MG 患者进行治疗,具有极大的临床研究和应用价值。

参考文献

[1] 晏琳,罗梦川,杨欢. 重症肌无力的免疫机制研究进展[J]. 中国神经免疫学和神经病学杂志,2012,19(6):419-424.

[2] 冯慧宇,刘卫彬,邱力,等. 他克莫司治疗难治性重症肌无力 36 例疗效与安全性的观察[J]. 中华医学杂志,2012,91(45):3190-3192.

[3] Pouwels S,de Boer A,Javaid MK,et al. Fracture rate in patients with myasthenia gravis:the general practice research database[J]. Osteoporosis International,2013,24(2):467-476.

[4] Kohler S,Keil TOP,Swierzy M,et al. Disturbed B cell subpopulations and increased plasma cells in myasthenia gravis patients[J]. Journal of neuroimmunology,2013,264(1):114-119.

[5] 蒋觉安,薛群,刘翠平,等. 协同刺激分子 B7-H3 在重症肌无力患者外周血中的表达[J]. 细胞与分子免疫学杂志,2012,28(8):856-859.

[6] 中国免疫学会神经免疫学分会,中华医学会神经病学分会神经免疫学组. 重症肌无力诊断和治疗中国专家共识[J]. 中国神经免疫学和神经病学杂志,2012,19(6):401-408.

[7] 王道宇. 重症肌无力患者无肌松剂进行气管插管和全身麻醉维持的可行性[J]. 中国医药指南,2012,10(25):160-161.

[8] Huijbers MG,Zhang W,Klooster R,et al. MuSK IgG4 autoantibodies cause myasthenia gravis by inhibiting binding between MuSK and Lrp4[J]. Proceedings of the National Academy of Sciences,2013,110(51):20783-20788.

[9] 陈宇,王化冰,王拥军,等. MuSK 特异性自身免疫性重症肌无力大鼠模型的建立[J]. 中华医学杂志,2013,93(17):1292-1296.

[10] 陈玉萍,王卫,王中魁,等. 重症肌无力伴发胸腺瘤患者的临床特点分析[J]. 中华内科杂志,2012,51(8):623-625.

[11] Huijbers MG,Zhang W,Klooster R,et al. MuSK IgG4 autoantibodies cause myasthenia gravis by inhibiting binding between MuSK and Lrp4[J]. Proceedings of the National Academy of Sciences,2013,110(51):20783-20788.

[12] Marx A,Pfister F,Schalke B,et al. The different roles of the thymus in the pathogenesis of the various myasthenia gravis subtypes[J]. Autoimmunity reviews,2013,12(9):875-884.

收稿日期:2014-06-23

(C)1994-2024 China Academic Journal Electronic Publishing House. All rights reserved. http://www.cnki.net

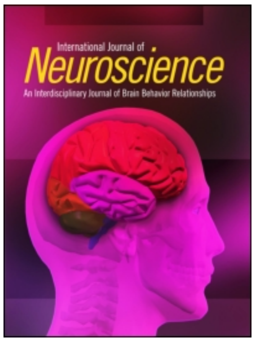

Taylor & Francis
Taylor & Francis Group

International Journal of Neuroscience

ISSN: 0020-7454 (Print) 1543-5245 (Online) Journal homepage: https://www.tandfonline.com/loi/ines20

The correlation of neutrophil-to-lymphocyte ratio with the presence and short-time curative effect of myasthenia gravis in children： a retrospectively study

Zhi Jiang, Zeshu Ning, Liming Yang, Bo Chen, Jingwen Tang, Jie Zhang, Hongjun Fang, Rong Xu, Feng Guo, Mei Chen & Kaisheng Sun

To cite this article: Zhi Jiang, Zeshu Ning, Liming Yang, Bo Chen, Jingwen Tang, Jie Zhang, Hongjun Fang, Rong Xu, Feng Guo, Mei Chen & Kaisheng Sun (2020): The correlation of neutrophil-to-lymphocyte ratio with the presence and short-time curative effect of myasthenia gravis in children：a retrospectively study, International Journal of Neuroscience, DOI: 10.1080/00207454.2020.1759592

To link to this article: https://doi.org/10.1080/00207454.2020.1759592

Accepted author version posted online: 21 Apr 2020.

Submit your article to this journal

View related articles

View Crossmark data

Full Terms & Conditions of access and use can be found at
https://www.tandfonline.com/action/journalInformation?journalCode=ines20

Check for updates

The correlation of neutrophil-to-lymphocyte ratio with the presence and short-time curative effect of myasthenia gravis in children：a retrospectively study

Zhi Jiang[a]，Zeshu Ning[a], Liming Yang[a]，Bo Chen[a]， Jingwen Tang[a]， Jie Zhang[a]，Hongjun Fang[a]，Rong Xu[a], Feng Guo[a]， Mei Chen[a], Kaisheng Sun[b]

[a]Neurology department of Hunan children's hospital, Changsha, China

[b]Institute of Pediatric Medicine，Hunan children's hospital, Changsha,China

Corresponding author Zhi Jiang，MM Department of Neurology，Hunan Children's hospital，No.86, ZiYuan Road, Yuhua District,Changsha 410007,China Tel +86-0731-85356336 E-mail jiangzhi1234@163.com

Accepted Manuscript

Objectives: This study aimed to investigate the influence of neutrophil-to-lymphocyte ratio (NLR) on the severity and short-time curative effect of myasthenia gravis (MG) in children.

Methods: Data of 132 MG children were retrospectively analyzed, and data of 140 healthy controls (HC group) in the same period were collected. The data of both groups were compared and analyzed.

Results: NLR of MG group was significantly higher than that of HC group (Z=2.644, P=0.008). According to NLR level, patients were divided into 3 groups: N1 (NLR<1.03), N2 (NLR 1.03-2.17), and N3 (NLR>2.17). Significant differences in white blood cell counts, course of disease, uric acid, albumin and the time of hospital stay among the 3 groups were observed (P<0.05, 0.01). The results of logistic regression revealed that NLR (adjusted OR=3.874, 95% CI 1.359-11.045, P=0.011) was the risk factor of MG, and it was risk factor of higher QMG during admission (adjusted OR=2.989, 95% CI 1.247-7.160, P=0.014) as well. Using the NLR level for the MG diagnostic test，the area under the receiver operating characteristic (ROC) curve was 0.765 [95%CI (0.710-0.820)，P=0.000], with a cut-off value of 1.39, sensitivity of 0.833, and specificity of 0.479. Cox regression analysis suggested that NLR (N1: Wald=9.262, P=0.010, N2: HR=12.267, 95%CI 2.432-61.863, P=0.000, and N3: HR=8.142, 95%CI 1.209-77.754, P=0.032) was associated with poor efficacy at discharge. Elevated NLR was considered as an independent risk factor of poor outcomes during discharge.

Conclusion: NLR could reflect disease severity and short time curative effect in children with MG to some extent. It may also be a potential marker in indicating diagnosis and severity of MG in children.

Key Words Myasthenia gravis, neutrophil-to-lymphocyte ratio, children,correlation

INTRODUCTION

Myasthenia gravis (MG) is an acquired autoimmune disease that is mainly mediated through acetylcholine receptor (AChR) antibodies[1,2]. Both cellular immunity and complement system are involved in the pathological process of MG, resulting in neuromuscular junction transmission

disorder, and is generally manifested as skeletal muscle weakness[3,4]. Its etiology and pathogenesis are poorly understood till date, and few previous studies have confirmed the involvement of inflammatory responses in its pathogenesis[1,5,6]. Cytokines related to inflammatory response, such as interleukin-6 (IL-6), interleukin-17 (IL-17) and tumor necrosis factor-alpha (TNF-α) were significantly increased in MG patients when compared with normal controls[7,8], and some inflammatory proteins were also increased[9]. This suggested that inflammation is also involved in the disease. However, it is still unclear regarding the role of inflammation in the process of MG and as to which extent does it affect on the outcome of the disease.

Neutrophil-to-lymphocyte ratio (NLR) is the ratio of neutrophils to lymphocytes in the peripheral blood, which is related to the degree of inflammatory response of diseases, and this may increase in diseases such as tumor, infection and chronic inflammation [9,10]. A study on MG in adults confirmed a correlation between NLR and the presence and activity of MG [11]. Moreover, MG has no optimal inflammatory or biological markers that reflect well with the disease severity.

There are some differences between the MG disease of children and adults, which are as follows: 1) MG in children is mainly ocular muscle type, and the rate of AchR antibody positive is lower than adults; 2) the incidence of MG crisis in children is lower than that in adults; 3) thymoma and hyperplasia in children are rarer than that in adults. It is well known that the proportion of granlocytes and lymphocytes in children varies with age. Previous studies have reported the association of NLR with MG in adults[1]. It is unclear whether this is true for children with MG. Hence, this study aimed to investigate whether NLR showed correlation with disease severity and short time curative effect of MG. Can NLR be used as a maker for diagnosis or severity of MG children?

1 METHODS

1.1 Study subjects

A total of 184 han nationality children from south China with MG who were admitted in the department of neurology of Hunan children’s hospital from January 2012 to September 2019 were selected. Fifty two patients with infection, thyroid dysfunction, self-discharge were excluded. Inclusion criteria were as follows: (1) patients with typical clinical symptoms, medical history (eyelid drooping or limb weakness, symptoms with volatility, etc.) and positive fatigue test, and confirmed by neostigmine test; (2) age ≤14 years; (3) patients with initial onset of the disease who did not undergo treatment with hormones and bromopizimine, and without using drugs such as intravenous immunoglobulins (IVIG) that may affect the immune function. Exclusion criteria were as follows: (1) MG combined with infection, thymoma, thyroid dysfunction, and other autoimmune diseases; (2) blepharoptosis caused by using penicillamine or other reasons such as mitochondrial encephalomyopathy; (3) patients with serious systemic diseases, and other conditions such as failed to discharge as prescribed, incomplete data and other information. A retrospective study was conducted to select 140 cases of age and gender matched children from the department of children’s healthcare (Healthy normal children about to be vaccinated) in our hospital during the same period was taken as the control group. Inclusion criteria were as follows: 1. healthy body, normal development, no obvious infectious diseases, allergic diseases, etc. 2. no hyperthyroidism, diabetes

Accepted Manuscript

and other diseases that require surgery and metabolic diseases were found. Exclusion criteria were as follows: 1. dysfunction of heart, liver and kidney or other chronic diseases such as bronchial asthma; 2. immunological diseases such as rheumatism that might affect the results of this study. This study was approved by the ethics committee of Hunan children's hospital (IRB No. KS 2019-07).

1.2 Medical history collection and clinical data

Detailed medical history data of children were collected retrospectively, which included their weight, age, course of the disease, medication, history of liver and kidney diseases, etc.

Laboratory test results and clinical data: blood routine, electrolyte, biochemical tests and other results of fasting blood samples of children on day 1 of admission were collected retrospectively. Clinical data, quantitative myasthenia gravis (QMG) score at the time of admission and at discharge were collected. Therapeutic effect and severity score were evaluated according to QMG proposed by Myasthenia Gravis Foundation of America (MGFA) [12]. This scoring method quantitatively tests the muscle strength of major muscle groups such as extraocular muscles and facial muscles, respectively, and adopts the 4-point system: normal count 0 score, and count 1 ~3 points respectively according to the (low, intermediate and high)of muscle weakness. The accumulated score of each muscle group is the QMG score of the patient, and the total score is 0-39 points. QMG1 was an admission score and QMG2 was a discharge score. We considered that the improvement of QMG(at least 1 point reduced) after treatment was effective, otherwise it woud be invalid.

1.3 Treatment and outcome events

According to the patient's condition and their parents' informed consent, the treatment plan was divided into four groups: 1) bromopizidine alone, 2) prednisone and bromopizidine, 3) methylprednisolone followed by 4) prednisone and bromopizidine, IVIG. The starting point of the observations was day 1 after admission of the child, and the outcome event was recorded on the day of discharge, and hospital stay was defined as day 1 after admission till discharge.

1.4 Follow-up

Patients were followed up after discharge, initially at every 15 days and then every 3 months, and the follow-up period completed on May 30, 2018. Failed treatment during discharge was treated as a failure event, and the time of failure event was recorded, and lost visit was taken as the cutoff data.

1.5 Statistical analysis

All statistical analyses were completed using SPSS 19.0 for Windows (IBM Corp, Armonk, NY, USA). The measurement data were first tested for normality. For example, the normal distribution data was presented as x±s, while skewed distribution was presented as M (P25, P75). The mean values of normal distribution data was done using t test or t' test, while the enumeration data or non-normal distribution was done using the non-parametric chi-square test or Mann-whitney U test. Normal distribution data were analyzed by Pearson correlation analysis and non-normal distribution

Accepted Manuscript

data by Spearman correlation analysis. Logistic regression analysis was performed with QMG score as dependent variable, and gender, course of disease, and NLR were taken as independent variables. Univariate regression was performed first, followed by stepwise multivariate regression analysis. The ROC curve was drawn with NLR test variables, and the case group and the control group were regarded as status markers. The outcome events were observed after discharge, and Cox regression analysis was performed for single factor and multiple factors. $P<0.05$ was considered to be statistically significant.

2 RESULTS

2.1 Baseline characteristics

General data and comparison of NLR were performed in 272 children. There were 132 cases in the case group (82 cases under 4 years, 22 cases between 4 and 6 years, and 28 cases over 6 years) and 140 cases in the control group (84 cases under 4 years, 26 cases between 4 and 6 years, and 30 cases over 6 years). In the case group, the NLR was 1.35(0.67, 1.94), and in the control group, it was 0.96 (0.57, 1.55), respectively, ($Z=2.644$, $P=0.008$). There were no significant differences between the two groups in age, gender, weight, neutrality, lymphoid function, platelet, total bilirubin, direct bilirubin, inosine, and total bile acid ($P>0.050$. The results are shown in table 1. The Myasthenia Gravis Foundation of America (MGFA) classification were as follow: I type 108 (81.82%), II type: 21(15.91%), III type 2 (1.52%), IV type 1 (0.76%), V type 0(0.0%). There were 4 cases of myasthenic crisis with a NLR of 1.669 and a maximum of 2.823. Only 22 children had completed cytokine assay, and their IL-6 level was 2.68 (1.78, 6.61) and TNF-α level was 2.19 (0.96, 4.90).

According to the level of NLR, the patients were divided into 3 groups: N1 (NLR<1.03), N2 (NLR 1.03–2.17), and N3 (NLR>2.17). Comparison of the 3 groups showed statistical significance in age, course of disease and albumin. Comparison of 3 groups' general data and NLR are shown in table 3.

2.2 Association of age, QMG, uric acid and albumin with NLR

Different age groups were compared, and the results revealed significant differences in NLR in different age groups ($P<0.01$, 0.05, table 2). Spearman correlation coefficient between NLR and age in case group showed significant results ($\rho=0.587$, $p=0.000$). Spearman correlation coefficient between NLR and age in HC group showed significant correlation ($\rho=0.613$, $p=0.000$). The correlation coefficient of NLR with uric acid and albumin, QMG1, and QMG2 were $\rho=-0.295$, $p=0.001$; $\rho=-0.228$, 0.003; $\rho=0.361$; 0.000, and $\rho=0.238$; 0.018, respectively in the case group. The correlation coefficient of uric acid and albumin was $r=0.225$, $P=0.016$ in the case group. The correlation coefficient of age with QMG1 and QMG2 was $\rho=0.183$, $p=0.018$, $\rho=0.157$, and $p=0.014$, respectively. Age showed mild relation with QMG. The correlation coefficient between disease duration and NLR was $\rho=0.215$, $p=0.012$, The correlation coefficient between NLR and IL-6, TNF-α were $\rho=0.434$, $p=0.032$,$\rho=0.476$, $p=0.029$. The correlation coefficient between IL-6 and TNF-α was $\rho=0.597$, $p=0.004$. The correlation coefficient between age and IL-6, TNF-α were $\rho=0.203$, $p=0.365$, $\rho=0.136$, $p=0.547$. There was no correlation between age and cytokines.

Accepted Manuscript

2.3 Elevated NLR levels is related to MG risk of disease and severity

Univariate logistic regression analysis revealed that gender, age, course of disease, weight, etc. showed no significance (P>0.05), while albumin (OR=0.384, 95%CI 0.179-0.825, P=0.014), uric acid (OR=0.139, 95%CI 0.063-0.307, P=0.000), and NLR (OR=2.378, 95%CI 1.517-3.728, P=0.000) were considered as risk factors of MG (P<0.01, 0.05). Further multi-factor regression analysis found that uric acid (OR=0.155 95%CI 0.060-0.397, P=0.000), albumin (OR=0.138, 95%CI 0.002-0.656, P=0.024), and NLR (OR=3.874, 95%CI 1.359-11.045, P=0.011) showed statistical significance (P<0.01, 0.05), in which the NLR was considered as risk factor of MG and the rest were considered as protective factors. The area under the ROC curve was 0.765 [standard error 0.028， 95%CI (0.710-0.820），P=0.000], with a cut-off value of 1.39, sensitivity of 0.833, and specificity of 0.479， see Fig.1. Regression of single factor logistic of QMG showed statistical significance for age (OR=1.780, 95%CI 1.043-3.038, P=0.035), albumin (OR=0.373, 95%CI 0.200-0.695, P=0.002), uric acid (OR=0.177, 95%CI 0.078-0.405, P=0.00) and NLR (OR=3.499, 95%CI 1.787-6.852, P=0.000). Multiple logistic regression found that albumin, uric acid and NLR were risk factors with high QMG score at admission (P<0.01, 0.05), and the results were consistent with those of MG risk factor analysis (P<0.01, 0.05), see table 4.

2.4 The association of NLR with short time curative effects of MG

Univariate regression found that albumin (HR=0.483, 95%CI 0.308-0.763,

P=0.002) globulin (HR=0.532, 95%CI 0.331-0.857, P=0.010) uric acid, (HR=0.383, 95%CI 0.253-0.581, P=0.000) and NLR (HR=3.950, 95%CI 2.555-6.109, P=0.000) showed significance with short time curative effects of MG. Further multivariate regression found that albumin, uric acid, and NLR showed significance with short time curative effects of MG. N1 (<1.03) with NLR as the reference value, and N2 (1.03-2.17), and N3 (>2.17) as relative risk factors showed no improvement in MG symptoms at the time of discharge [HR=12.267 and 8.142 (P<0.01, 0.05)], table 5. As shown in Fig 2, the inefficiencies of inpatient treatment in N1, N2, and N3 groups were 9.76% (4/41), 21.28% (10/47), and 43.18% (19/44), X^2=13.186, P=0.01.

3 DISCUSSION

The etiology of MG is still not completely clear [13,14], and is mainly related to B and T cells and complement activation [15,16]. Inflammation also plays a vital role in the pathogenesis of MG. Activation of B and T cells and activation of complement system secretes cytokines in different degrees [17-20], and some are inflammatory cytokines that can trigger inflammatory responses. TNF-α in MG patients is increased when compared with normal individuals, and is significantly decreased after improvement in clinical symptoms [21]. Also the levels of TNF-α were significantly different in different MG patients, which is not completely consistent with the severity of MG symptoms and the level of AChR-ab titer [22]. TNF-α is a major pro-inflammatory cytokine that induces a wide range of inflammatory cascade reactions, and IL-6 also acts as a pro-inflammatory cytokine that is significantly increased during inflammatory responses, and both these showed close correlation with infectious diseases[23]. In this study, due to cost or other reasons (such as restrictions of medical

Accepted Manuscript

insurance policy, etc), only a few children have completed IL-6, TNF-α detection. Although the number of cases was not so many, the relationship of NLR with these two cytokines have been confirmed.

MG patients have all kinds of Th cells in the body, such as Thl cells mainly the proinflammatory cytokines including IFN-γ, IL-2. IFN-γ is an important cytokine secretion, wherein the Thl cells have been shown to participate in a variety of autoimmune and infectious diseases. MG is associated with Th17 cells and their secreted cytokines. Cytokines such as IL-17A, IL-17F, IL-21, IL-22 and TNF-α induce and maintain inflammatory responses in tissues [7, 8, 24]. Because this study is a retrospective study, and these tests are expensive and have not finished (Our hospital can not test IL-17A, IL-17F, IL-21, IL-22 and other factors). We can’t do any further analysis. Complement system is also involved in the course of MG disease. In MG patients, there is an obvious increase in complement consumption, and slight decrease of C3 and C4 complement systems[23]. After activation of complement system, C3a, C4a and C5a were generated, causing inflammatory reactions as allergic toxins.

Monitoring of immune indicators might be helpful in assessing the severity of patients. However, monitoring of immune parameters remains complicated and expensive, which is not suitable for repeated application. It is necessary to use a simple and feasible indicator for evaluating MG. Monitoring of inflammatory responses might also be helpful in assessing the extent of diseases in children with MG, which remains to be further studied. The prognosis of children with MG is also a concern. Because the data was not fully collected, we had no further discussion. .

NLR is a simple and useful indicator for evaluating the inflammatory response, and is correlated with the prognosis of tumors and reflects the inflammatory state of patients with tumors and chronic inflammation [25]. Studies have revealed that other diseases, such as myocardial infarction and cerebral infarction, also involve changes such as increased NLR[19]. Previous evidence suggested that chronic inflammation also involves pathological process of MG[26,27]. NLR increase indicates active inflammatory response, and this might be related to the severity of MG. The correlation between NLR and QMG score in this study was not very high, suggesting that inflammation was not the main cause of the disease. Currently, there are studies confirming that the NLR has been changing in MG patients, and the NLR of adult MG patients is too high, and is associated with high risk and poor prognosis [11]. Our results were consistent with this study. However, currently, there is no direct evidence that NLR is related to the severity of MG disease in adult MG patients, and the exact reasons are still unclear. Also the cause of high NLR in MG patients is not clear. The mechanism of high NLR in MG patients was not involved in this study and warrants further investigation.

NLR is different in normal children and adults, and similarly the MG performance or prognosis is different in children and adults. NLR in children varies with age. For normal children, the granulocyte ratio is about 0.65, and the lymphatic ratio is about 0.30. The granulocyte ratio then gradually declines with increasing age, at 4 to 6 days of birth and 4-6 years of age the neutral grains and lymphocyte ratio are equal. After 6 years, the neutral grain and lymphocyte distribution of children were similar to that of adults. But no such changes were observed in normal or diseased adults. This study found that compared with normal group, MG in children of all age groups had higher NLR, suggesting a certain inflammatory response. In the normal control group, the influence of age on NLR was obvious, and the NLR was different in different age groups. The influence of age on NLR in case group showed no significance when compared with that of normal control group.

Accepted Manuscript

There was no significant difference in NLR between patients less than 4 and 4-6 years and 4-6 years and those aged more than 6 years. A significant difference was observed in children under 4 years and over 6 years. Age-related analysis of NLR in both groups also confirmed this, and this change might be related to MG. Furthermore, logistic regression analysis showed that age did not enter the regression equation, and this has also been proved the above results. In addition, in children, NLR is affected by age, which is not relevant for cytokines. If this effect is to be eliminated, the influence of age would be limited. Therefore, it seems that NLR may not be able to act as a perfect proxy inflammatory factor for MG. Whether NLR can replace cytokines or act as a inflammatory factor need further study. (We have too little data to complete the study.)

Our study confirmed a mild positive correlation between disease duration and NLR, suggesting that the NLR increases gradually as the disease progresses, which is well understood.As the disease progresses, the symptoms become more apparent, the disease becomes more severe, and the NLR will be higher. All the NLR levels here were the results before treatment, and the results after treatment and long-term follow-up have not been collected. Further research need to be done on the effects of treatment on NLR. The course and severity of the disease were correlated with the NLR (with a low correlation coefficient), which seemed to have potential as a diagnostic marker.

In this study, we found that children with MG had higher NLR than normal control group, and the results were similar to those studies in adults. Logistic regression suggested that high NLR showed association with high MG risk. NLR showed low correlation with QMG score at the time of admission and at discharge, suggesting a correlation with MG severity. Furthermore, the correlation was high at admission than at discharge, indicating that NLR could reflect QMG changes and severity of the disease. Unfortunately, due to lack of data (some of the children were not able to undergo blood routine test when they are discharged from the hospital), and otherwise, the results remain more convincing if the dynamic NLR was correlated with QMG. NLR showed correlation with uric acid, albumin, etc. According to a study, there is a confirmed a decrease in uric acid in MG patients[28]. Previous literature has confirmed that uric acid is negatively correlated with disease severity of MG[13], and NLR is correlated with uric acid, and so NLR is related to MG disease. Studies have confirmed that low albumin showed association with MG severity [18], and due to low correlation between NLR and albumin, the level might be related to the severity of MG. As the data of case group was non-normally distributed, it cannot be further verified by multiple linear regression. In this study, only logistic regression analysis was selected, and the correlation and regression coefficients remained relatively lower than the regression of parameters.

Univariate logistic regression analysis found that the high QMG score at admission was related to uric acid, albumin, and NLR in children. Multi-factor regression analysis found that OR values were statistically significant, indicating that high albumin, NLR and low uric acid levels were independent risk factors with high QMG score. The ROC curve showed 1.39 as the cut-off value for NLR, the sensitivity was 0.833, and the specificity is 0.479. The specificity is too low,which is not very significant for the diagnosis of MG. There were differences in the inefficiencies when children in the 3 groups were discharged from the hospital. Our study found that there were significant differences in age and course of disease among the three groups (according to NLR level).It suggests that low age, short course of disease lead to low NLR level. It may be due to the younger the child, the more concerned the parents were, the earlier see the doctor. On the contrary, the older the children, the later see the doctor. NLR's influence on the time of hospital stay was obvious, as shown in Table3. The NLR and MG criss correlation was not obvious, but the adult studies suggest a correlation

Accepted Manuscript

[11].Because we had a relatively few the number of cases,there were low incidence of criss MG.Actually the incidence of MG criss in children is lower than adults.

Statistical significance was observed in the HR values of N2 and N3 groups when further Cox regression analysis with NLR, using N1 as reference was performed. This suggested that the possibility of failure was greater with increasing NLR. Our study confirmed that NLR in MG children was increased with increased hospitalization time. High NLR in MG children meant that the inflammatory response *in vivo* is relatively severe. No matter whether it is caused by excessive complement activation or excessive cytokine production, the severity of the disease is still indicated. Both correlation and regression analysis supported the above conclusions.

In conclusion, NLR might be related to the disease condition of MG in children, and it might reflect the severity and short time curative effect of the disease to a certain extent. It may be a simple and useful potential indicator of diagnosis and severity of MG in children.

Conflicts of interest

The authors report no declarations of interest.

Acknowledgements

The authors thank and acknowledge all of the participants who were enrolled in this

Study.

Accepted Manuscript

REFERENCES

1, Barraud C，Desguerr I，Barnerias C，Gitiaux C,Boulay C,Chabrol B. Clinical features and evolution of juvenile myasthenia gravis in a French cohort.Mu-scleNerve,2018,57:603-609.
2，Huang S,Wang W,Chi L. Feasibility of up-regulating CD4+CD25+ Tregs by IFN-Y in my asthenia gravis patients. BMC Neurology,2015,15:163
3，Molin CJ,Westerberg E,Punga AR.Profile of upregulated inflammatory prote-ins in sera of myasthenia gravis patients. Scientific reports，2017,7:39716.
4, Uzawa A,Kanail T,Kawaguchi N,Oda F,Himuro K,Kuwabara S. Changes in inflammatoryc ytokine networks in myasthenia gravis.Scientific reports,2016,5:25886-258990.
5，Luo J,Lindstrom J.AChR-specific immunosuppressive therapy of myasthenia gravis.Biooche m Pharmacol.2015,97:609-619.
6,Jing F,Yang F, Cui F, Ling LHuang X.Rapamycin alleviates inflammation and muscle weak ness,while altering the Treg/Th17 balance in a rat model of myasthenia gravis.Bioscience rep orts 2017,37(4).
7,Shahgah AG,Fattahi E,Shahneh FZ. Interleukin-17 in human inflammatory dis-ease.Postepy Dermatol Altrgol.2014,31(4):256-261.
8,Molin CJ,Westerberg E, Punga AP. Profile of upregulated inflammatory prot-eins in sera of myasthenia gravis patients.Scientific reports，2017,7：39716.

9, Chen G,Zhu L,Yang Y,Long Y,Li X,Wang Y. Prognostic role of neutrophil-to-lymphocyte ratio inovarian cancer: a meta-analysis.Technology in Caner & Treatment,2018, 1:17.

10，Kaushik R,Gupta M,Sharma M,Jash D,Jain N,Sinha N,et al.Diagnostic and prognostic role of neutrophil-to-lymphocyte ratio in early and late phase of sepsis.Indian J Crit Care Med. 2018,22(9): 660–663.

11，Yang DH,Qian MZ,Wei MM,Li J,Yu MM,Lu XM,et al. The correlation of neutrophil-to-lymphocyte ratio with the presence and activity of myasthenia gravis. Oncotarget,2017,8(44):76099-76107.

12.Jaretzki A 3rd，Barohn RJ，Ernstoff RM，Kaminski HJ,Keesey JC,Penn AS,et al. Myasthenia gravis：recommendations for clinical research standards. Task Force of the Medical Scientific Adisory Board of the Myasthenia GravisFoundation of America. Ann Thorac Surg，2000, 70（1）：327-334.

13, Yang D,Weng Y,Lin H,Xie F,Yin F,Lou K,et al. Serum uric levels in patie-nts with myasthenia gravis are inversely correlated wirh disability. NeuroReport, 2016, 27(5) : 301-305.

14，Uzawa A,Kawaguchi N,Kanai T,Himuro K,Oda F,Kuwabara S.Increased ser-um peroxiredoxin 5 levels in myasthenia gravis. J Neuroimmunol.2015,287：16-18.

15, Uzawa A,Kawaguchi N,Kanai T,Himuro K,Kuwabara S.Serum high mobilitygroup box 1 is upregulated in myasthenia gravis. J Neurol Neurosurg Psychiat-ry.2015,86:695-7.

15，Gihus NE,Verschuuren JJ. Myasthenia gravis：subgroup classification and therapeutic strategies.Lancet Neurol. 2015,14:1023-36.

17,Uzawa A,Kawaguchi N,Himuro K,Kanai T,Kuwabara S.Serum cytokine and chemokine profiles in patients with myasthenia gravis. Cin Exp Immunol.2014,176:232-237.

18,Weng YY, Yang DH,Qian MZ,Wei MM,Yin F,Li J,et al.Low serum albumin concentrations are associated with disease severity in patients with myasthenia gravis. Medicine,2016,95:39-44.

19, Lee YS,Nam HS,Lim JH,Kim JS,Moon Y,Cho JH,et al. Prognostic impact of a new score using neutrophil-to-lymphocyte ratios in the serum and malignant pleural effusion in lung cancer.BMC Cancer 2017,17:557

20，Zhang Y,Lu JJ,Du YP,Feng CX,Wang LQ,Chen MB.Prognostic value of ne-utrophil-to-lymphocyte ratio and platelet-to-lymphocyte ratio in gastric cancer.medicine,2018,97:12.

21，Guan YZ,Gui LY,Li YF, Zhang JB.Tumor necrosis factor-α polymorphism and secretion in myasthenia gravis.CLin Med Sci,2005,20(2):104-107.

22,Lee JS,Joo IS,Sook TL.Widely varying TNF-alpha levels in patients with myasthenia gravis.Neurol Sci，2009，30（3）：259-262.

23，Aricha R,Mizrachi K,Fuchs S,Sourojon MC. Blocking of IL-6 suppresses experimental autoimmune myasthenia gravis.J.Autoimmun,2011,36:135-141.

24，CaoY，Amezquita RA，Kleinstein SH,Stathopoulos P,Nowak RJ,O’Connor KC.Autoreative T cells from patients with myasthenia gravis are characterized by elevated IL-17, IFN-Y,and GM-CSF and diminished IL-10 production. J Immunol,2016,196(5):2075-84.

25，Kusner LL,Kaminski HJ.The role of complement in experimental autoimm-une myasthenia gravis.Ann N Y Acad Sci,2012,1274:127-132.

26, Truffault F,de Montpreville V, Eymard B,Sharshar T,Le Panse R, Berrih-A-knin S. Thymic germinal centers and corticosteroids in myasthenia gravis：an immunopathological study in 1035 cases and a critical review.Clin Rev Allergy Immunol,2016.52(1):108-124.

27，Cavalcante P,Cufi P,Mantegazza R, Berrih-Aknin S,Bernasconi P,Le Panse R. Etiology of myasthenia gravis：innate immunity signature in pathological thymus.Autoimmun Rev,2013,12:

Accepted Manuscript

863-874.
28,Fuhua P,Xuhui D,Zhiyang Z,Ying J,Yu Y,Feng T,et al.Antioxidiant status of bilirubin and uric acid in Patient with myasthenia gravis.Neuroimmunomodulati-on,2012,19(1):43-9.

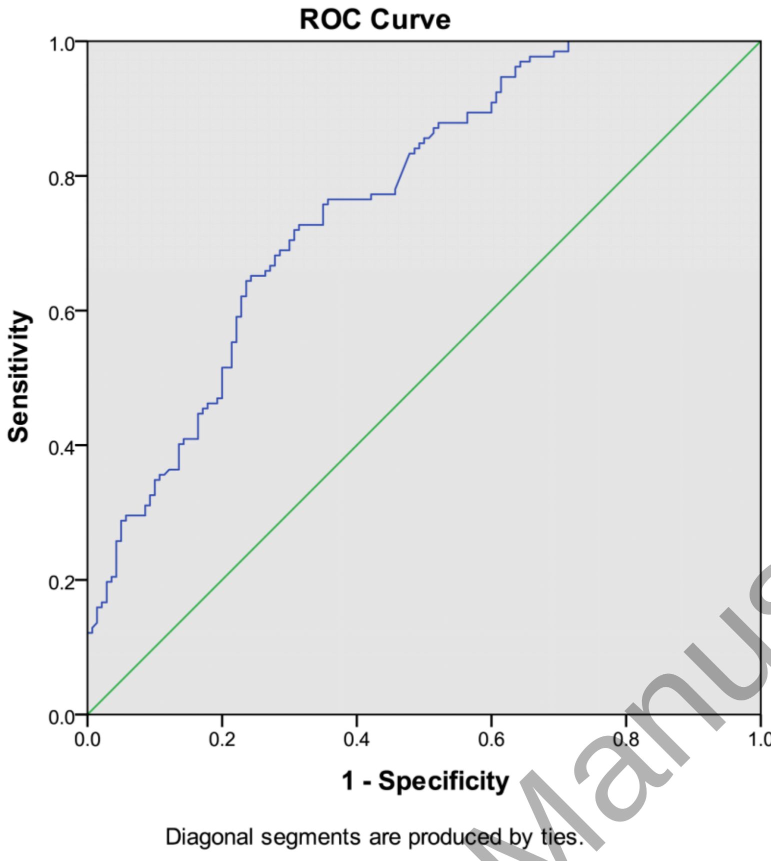

Fig.1 Prediction of ROC of MG based on NLR level

Accepted Manuscript

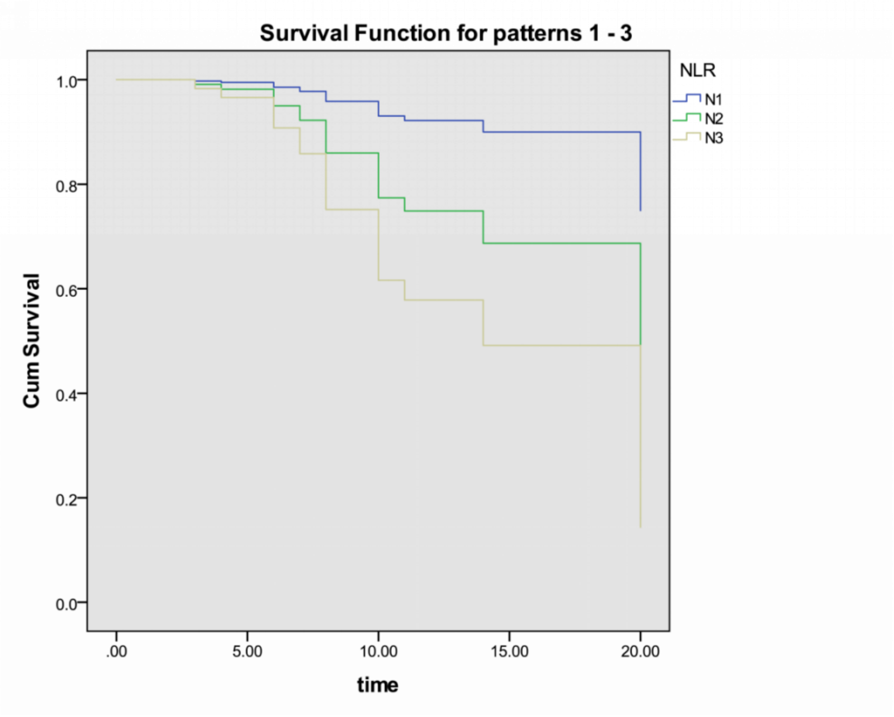

Fig.2. The influence of different NLR levels in MG children on the curative effect at discharge

Accepted Manuscript

Table 1. Comparison of clinical data between MG patients and healthy controls

	Case group (n=132)	Control group(n=140)	value	P
Age	2.92（2.08,5.83）	3.42（1.75,6.75）	0.763	0.445
Sex (male/female)	62/70	67/73	0.021	0.884
Body weight (kg)	14.0(12.0,19.0)	16.3(12.0,23.8)	0.993	0.321
Duration (day)	20（7,60）	-	-	-
neutrophil-to-lymphocyte ratio	1.35（0.67,1.94）	0.96(0.57,1.55)	2.644	0.008
WBC ($X10^9/L$)	9.18±2.70	8.11±2.56	3.327	0.001
Neutrophil ($X10^9/L$)	4.21(2.90,5.80)	3.35(2.38,4.52)	0.978	0.328
Lymphocyte ($X10^9/L$)	3.54(2.56,5.00)	3.38(2.65,4.69)	0.404	0.686
Platelet ($X10^9/L$)	301.10±81.65	304.21±88.33	0.376	0.707
Hemoglobin (g/l)	123.50±9.44	121.75±10.11	1.473	0.142
RBC ($X10^{12}/L$)	4.64±0.43	4.56±0.45	1.549	0.122
Total bilirubin	7.97±3.18	9.42±3.65	3.511	0.001
Direct bilirubin	2.65±1.04	2.89±0.88	2.100	0.037
Indirect bilirubin	5.29±2.77	6.51±3.32	3.365	0.001
Total protein	64.71±4.78	65.46±5.27	1.238	0.217
Albumin	40.27±2.28	41.29±3.27	2.976	0.003
Globulin	24.29±3.53	24.55±4.17	0.568	0.571
Uric acid	238.16±66.54	265.27±73.87	3.183	0.002
Urea nitrogen	4.44±1.08	4.23±1.12	1.526	0.128
Creatinine	27.68±11.01	26.92±12.63	0.476	0.634
Total bile acid	6.15±2.65	6.20±2.98	0.146	0.884
QMG1	3(2,4)	-	-	-
QMG2	1(1,2)	-	-	-

Accepted Manuscript

Table 2. Influence of age on NLR in children with MG compared with healthy controls

Group	Less than 4 years old	4-6 years old	More than 6 years old
MG group	1.03 (0.55,1.80)	1.43 (0.76,2.21)	1.90 (1.41,2.46)
Control group	0.62 (0.42, 1.04)	0.98 (0.61,1.20)	1.37 (0.98,2.01)
value	3.074	2.345	2.215
P	0.002	0.019	0.027

Note: The statistical values were less than 4 years old group 17.496, P=0.000 and 4 to 6 years old group were -17.571, standard error 11.006, P=0.110, and less than 4 years old group and greater than 6 years old group were -33.125, standard error 8.807, P=0.000, standard error 8.807 and greater than 6 years old group were -15.555, standard error 12.380, P=0.209. The statistical values of less than 4 years old and aged 4-6 years old groups were -22.292, standard error was 9.486, P=0.019, the statistical values of less than 4 years old and over 6 years old groups were -40.664, standard error was 7.693, P=0.000, the statistical values of 4-6 years and over 6 years old groups were -18.372, standard error was 10.040, P=0.039.

Accepted Manuscript

Table 3. Comparison of clinical characteristics in MG patients grouped according to NLR level

	N1 (N=41)	N2 (N=47)	N3 (N=44)	value	P
Sex（male/female）	20/21	21/26	23/21	0.163	0.922
Age	2.08（1.75，3.00）	3.25（2.58，5.83）	6.08（3.69，9.33）	21.039	0.000
Duration	13（7，30）	14(7,56)	31(10,94)	7.090	0.029
Body weight	13.2（11.5，17.5）	14.3(11.7,18.5)	16.0(13.0,19.9)	3.619	0.164
IGG	7.61±1.48	8.30±2.00	8.62±2.97	2.207	0.114
IGA	0.82±0.41	0.87±0.53	0.94±0.56	0.656	0.521
IGM	1.21±0.46	1.28±0.52	1.19±0.43	0.381	0.684
IGE	30.3(13.75,91.05)	24.2(10.63,62.80)	28.64(13.46,53.64)_	1.674	0.433
C3	0.86±0.19	0.90±0.20	0.94±0.18	2.005	0.139
C4	0.17±0.06	0.19±0.06	0.20±0.07	1.660	0.194
Total bilirubin	8.09±3.23	8.04±4.85	9.04±3.64	0.596	0.553
Direct bilirubin	2.74±1.10	2.90±2.04	3.33±1.45	1.155	0.319
Indirect bilirubin	5.35±2.83	5.14±3.61	5.72±2.69	0.258	0.773
Total protein	66.50±5.72	66.25±5.16	67.01±5.85	0.216	0.806
albumin	41.86±4.01	39.61±5.50	38.17±7.41	4.277	0.016
globulin	24.15±2.42	24.82±3.45	24.93±4.40	0.598	0.552
Uric acid	256.40±64.79	237.54±66.72	219.30±64.33	3.424	0.036
Total bile acid	6.99±3.58	6.76±3.74	6.59±3.49	0.135	0.874
Creatinine	23.16±8.08	25.90±5.75	24.85±6.98	1.711	0.185
Urea nitrogen	4.32±1.16	4.52±1.10	4.56±0.99	0.394	0.675
WBC	8.41±2.84	9.06±2.45	10.01±2.64	3.986	0.021
Neutrophil	2.55(1.68,3.65)	3.90(2.88,4.82)	5.34(4.70,6.74)	58.959	0.000
Lymphocyte	4.92(3.50,6.22)	3.44(2.58,3.94)	2.27(1.70,2.79)	45.613	0.000
Infection history (n/y)	34/7	38/9	31/11	1.165	0.558
MG crisis（with/without）	0/44	1/46	3/41	3.560	0.169
Hospitalizatio	7.98±2.95	8.94±3.74	10.43±3.62	5.434	0.005

Accepted Manuscript

n time（day）					

Accepted Manuscript

Table 4. Multivariate logistic regression analysis of influencing factors of QMG score at admission in children with MG

	B	SE	Wald	Exp B	95%CI	P
Sex	-0.020	0.703	0.001	0.980	0.247-3.883	0.977
age	0.360	0.460	0.731	1.433	0.621-3.306	0.398
Total protein	-0.680	0.595	1.303	0.507	0.158-1.628	0.254
albumin	-1.138	0.493	5.333	0.320	0.122-0.842	0.021
globulin	0.144	0.560	0.066	1.155	0.385-3.462	0.797
Uric acid	-1.388	0.515	7.275	0.250	0.091-0.684	0.007
NLR	1.095	0.446	6.031	2.989	1.247-7.160	0.014
constant	-1.210	0.947	1.610	0.301		0.205

Accepted Manuscript

Table 5. Multivariate Cox analysis of factors affecting the efficacy of MG in children

	B	SE	Wald	HR	95%CI	P
Albumin	-0.695	0.327	4.511	0.499	0.263-0.948	0.034
Uric acid	-0.843	0.371	5.162	0.431	0.208-0.891	0.023
Treatment	-0.369	0.157	5.502	0.691	0.508-0.941	0.019
NLR N1			9.262			0.010
NLR N2	2.507	0.826	9.222	12.267	2.432-61.863	0.002
NLR N3	2.097	0.925	5.131	8.142	1.327-49.979	0.024

Accepted Manuscript

doi: 10.13241/j.cnki.pmb.2015.35.014

血清胆红素与尿酸水平对重症肌无力的临床意义 *

李 静[1] 汪毓君[1] 王 云[2] 李 丽[2] 霍 秋[2]
(1 华中科技大学同济医学院附属武汉中心医院 重症医学科 湖北 武汉 430014;
2 武汉大学附属医院 重症医学科 湖北 武汉 430014)

摘要 目的:探讨血清胆红素以及尿酸的水平对重症肌无力患者的临床价值。**方法:**选取我院确诊的重症肌无力患者作为实验组,另选择同期体检的健康志愿者作为对照组。检测并比较两组血清直接胆红素(DBIL)、间接胆红素(IBIL)、总胆红素(TBIL)、尿酸(UA)及肌酐(Cr)等指标水平。**结果:**实验组血清 DBIL、IBIL、TBIL、UA 水平均显著低于对照组,差异有统计学意义(P<0.05),但实验组不同类型的 MG 患者血清 DBIL、IBIL、TBIL、UA 水平比较差异均无统计学意义(P>0.05)。同组男性 DBIL、IBIL、TBIL、UA 值均明显高于女性,差异具有统计学意义(P<0.05);两组间相同性别比较,实验组 DBIL、IBIL、TBIL、UA 均低于对照组,差异有统计学意义(P<0.05)。**结论:**检测血清 BIL 和 UA 水平对诊断重症肌无力具有一定的参考价值。

关键词:胆红素;尿酸;重症肌无力;临床价值
中图分类号:R746.1 **文献标识码:**A **文章编号:**1673-6273(2015)35-6857-03

Effect of Serum Levels of BIL and UA in Myasthenia Gravis Patients*

LI Jing[1], WANG Yu-jun[1], WANG Yun[2], LI Li[2], HUO Qiu[2]
(1 Department of Severe Medicine, Wuhan Central Hospital, Wuhan, Hubei, 430014, China;
2 Department of Severe Medicine, Affiliated Hospital of Wuhan University, Wuhan, Hubei, 430014, China)

ABSTRACT Objective: To investigate the clinical value of BIL and UA levels in serum of patients with myasthenia gravis. Methods: 31 patients with myasthenia gravis who were diagnosed in our hospital were selected as the experimental group, and another 31 healthy people who had taken the examination were chosen to be the control group. Then the direct bilirubin (DBIL), indirect bilirubin (IBIL), total bilirubin (TBIL), uric acid (UA) and creatinine (Cr) were tested and compared between the two groups. Results: Compared with the control group, the levels of DBIL, IBIL, TBIL and UA in the experimental group were lower (P<0.05); There was no statistically significant difference about the levels of DBIL, IBIL, TBIL and UA of patients with different types of MG in the experimental group (P>0.05); Compared with the female patients in the same group, the levels of DBIL, IBIL, TBIL and UA were higher than those of the male patients (P<0.05). Compared with the control group, the levels of DBIL, IBIL, TBIL and UA of patients with the same gender were lower in the experimental group (P<0.05). Conclusion: The levels of BIL and UA in serum were important for the diagnosis of myasthenia gravis.

Key words: BIL; UA; Myasthenia gravis; Clinical value
Chinese Library Classification(CLC): R746.1 Document code: A
Article ID: 1673-6273(2015)35-6857-03

前言

重症肌无力(Myasthenia gravis,MG)是一种由神经 - 肌肉接头处传递功能障碍引起的自身免疫性疾病,其发病与乙酰胆碱传递障碍密切相关,并与 T 细胞、细胞因子、神经肌肉接头处抗体等密不可分[1,2],临床表现为部分或全身骨骼肌无力、易疲劳,活动后症状加重,休息会可减轻,若病情严重或医治不及时,可出现肌无力危象等症状,严重影响患者的健康及生活[3]。因此,及时诊断对 MG 患者的预后具有重要意义。

近年来研究发现抗氧化物质在自身免疫性疾病的发生发展过程中发挥着重要作用。胆红素(Bilirubin,BIL)是体内铁卟啉化合物的主要代谢产物,具有抗氧化剂功能,而尿酸(Uric acid,UA)则是体内嘌呤核酸分解代谢的终末产物[4]。本研究通过分析 MG 患者血清 BIL 及 UA 水平,旨在为 MG 早期诊断提供更多的参考依据。

1 资料与方法

1.1 一般资料

选取 2010 年 8 月至 2015 年 2 月在我院确诊为重症肌无力的患者 31 例作为实验组,其中男 12 例,年龄 17(5-40)岁;女 19 例,年龄 19(3-39)岁。另选择同期于我院进行体检的健康人 31 例作为对照组,其中男 12 例,年龄 18(6-40)岁;女 19 例,年龄 17(4-39)岁。两组间一般临床特征比较无统计学差异(P>0.05),如表 1。

* 基金项目:湖北省武汉市科技项目(WX12C35);湖北省自然科学基金项目(2011CDB479)
作者简介:李静(1985-),女,本科,主管护师,研究方向:主要从事危重症研究,电话:13797010252,E-mail:1493984185@qq.com
(收稿日期:2015-08-06 接受日期:2015-08-22)

(C)1994-2023 China Academic Journal Electronic Publishing House. All rights reserved. http://www.cnki.net

表 1 两组患者的一般临床资料比较

Table 1 Comparison of the general information between the two groups

General information	Experiment group	Control group
Case(n)	31	31
Gender (male / female)	12/19	12/19
Age (year)	18.35± 13.03	16.33± 12.75
Course of disease (year)	7.3± 4.8	0
Type I (ocular)	9	0
Type IIA (mild systemic type)	7	0
II B (moderate systemic type)	6	0
Type III (acute progressive)	6	0
Type IV (late type)	3	0
V type (muscular atrophy type)	0	0
AST (U/L)	29.75± 4.72	26.29± 5.17
ALT (U/L)	22.89± 3.85	21.93± 4.38
Cr (μmol/L)	57.38± 11.42	59.73± 12.27

1.2 诊断依据

根据中国免疫学会神经免疫学分会、中华医学会神经病学分会神经免疫学组于 2012 年制定的诊断标准[5] 排除标准 :①不符合以上诊断标准者 ;②有严重的心脑血管、肝、肾、血液等系统疾病或恶性肿瘤者 ;③患有其他自身免疫性疾病、代谢性疾病等 ;④有激素及免疫抑制剂治疗史者 ;⑤近三月内使用过影响胆红素和尿素代谢药物者 ;⑥任何原因无法配合治疗者。

1.3 检测方法

所有研究对象均采集空腹 12 小时后静脉血 5 mL,然后分离血清,采用全自动分析仪(日立 7600-120)测定血清胆红素(DBIL)、间接胆红素 (IBIL)、总胆红素 (TBIL)、谷草转氨酶(AST)、谷丙转氨酶(ALT)、尿酸(UA)、肌酐(Cr)等。

1.4 统计学方法

计量数据以均数± 标准差($\bar{x}$± s)表示,以 SPSS 19.0 统计软件进行单因素方差分析法,使用 Turkey 检验[6]进行组间检验、校正,以 $P<0.05$ 认为有统计学意义。

2 结果

2.1 两组血清 BIL 及 UA 水平的比较

实验组 DBIL、IBIL、TBIL、UA 水平均显著低于对照组,差异有统计学意义 ($P<0.05$);而实验组各分型之间血清 BIL 及 UA 水平比较差异均无统计学意义($P>0.05$)。见表 2。

表 2 两组血清 BIL 及 UA 水平的比较

Table 2 Comparison of the serum levels of UA and BIL between two groups

Group	Type	n	DBIL(μmol/L)	IBIL(μmol/L)	TBIL(μmol/L)	UA(μmol/L)
Experimental group	Type I	9	3.25± 1.07	7.56± 0.75	10.74± 2.95	273.23± 71.23
	Type II a	7	3.39± 1.21	8.03± 0.69	9.89± 2.57	252.71± 69.46
	Type II b	6	3.04± 0.97	7.29± 1.17	10.45± 2.14	269.89± 77.59
	Type III	6	2.95± 1.05	7.99± 1.08	11.01± 2.87	259.63± 65.31
	Type IV	3	3.18± 1.09	8.14± 0.89	9.97± 2.31	280.97± 72.37
	Total	31	3.17± 1.11*	7.83± 0.77*	10.21± 2.09*	262.75± 70.84*
Control group		31	3.85± 0.88	9.57± 1.04	15.27± 3.95	298.49± 76.57

Note: compared with the control group, *$P<0.05$.

2.2 两组不同性别患者血清 BIL 及 UA 水平的比较

同组男性 DBIL、IBIL、TBIL、UA 值均明显高于女性,差异具有统计学意义($P<0.05$) 对两组相同性别比较,实验组 DBIL、IBIL、TBIL、UA 均低于对照组,差异有统计学意义($P<0.05$),如表 3。

表 3 两组不同性别患者血清 BIL 及 UA 的水平的比较

Table 3 Comparison of the serum levels of UA and BIL between different genders in two groups

Group	Gender	n	DBIL(μmol/L)	IBIL(μmol/L)	TBIL(μmol/L)	UA (μmol/L)
Experimental group	Male	12	3.15± 1.17*#	8.53± 0.86*#	11.04± 2.95*#	283.33± 73.41*#
	Female	19	2.97± 1.01#	6.27± 0.69#	9.25± 2.17#	242.79± 67.96#
Control group	Male	12	3.94± 0.89*	9.98± 1.17*	16.45± 4.57*	312.89± 88.14*
	Female	19	3.72± 0.87	9.31± 0.95	14.02± 3.64	278.49± 70.18

Note: compared with female with groups, *$P<0.05$; Compared with the control group, #$P<0.05$.

(C)1994-2023 China Academic Journal Electronic Publishing House. All rights reserved. http://www.cnki.net

2.3 两组不同年龄患者血清 BIL 及 UA 水平的比较

两组患者相同年龄层血清 DBIL、IBIL、TBIL、UA 值进行比较，差异情况与组间整体比较情况完全一致(P＞0.05)，无特异性差异；同组内各年龄层之间比较并无统计学差异（P＞0.05)。

3 讨论

重症肌无力可导致患者部分或全身骨骼肌易疲劳、波动性的肌无力，严重者可累及呼吸肌，危及生命。在我国，重症肌无力的发病率约为(77-150)/100 万[7]，女性高于男性，约占患者总数的 60%，各年龄段均有发病，儿童 1-5 岁居多，且近年来发病率仍有上升趋势[8]。重症肌无力的临床诊断目前国内没有明确的诊断标准，依靠患者的临床表现、既往史、血清学检测、神经系统检查等进行综合判断。许多学者认为新斯的明试验是确诊 MG 的重要标准之一，检测血清乙酰胆碱受体抗体滴度水平也可对疾病程度及预后进行预测[9]。但也有研究表明 Lambert-Eaton 肌无力综合征、吉兰 - 巴雷综合征、运动神经元病等均会出现新斯的明试验阳性结果，乙酰胆碱受体抗体滴度也并不完全与患者的临床表现吻合，故其预测作用也并未得到完全的认可[10]。因此，寻求一种方法对 MG 进行早期明确诊断十分必要。

近年来，有研究发现胆红素具有抗炎、抗氧化、免疫调节等多种作用，其抗氧化作用可高于如维生素 E 等抗氧化物质数十倍[11]。而尿酸作为嘌呤的代谢终产物，是一种强效的氧、氮自由基清除剂，对过氧亚硝酸盐清除作用亦极强，在机体清除自由基的过程中，尿酸发挥的作用约可占到 60%[12]。二者均可在机体的抗氧化损伤中发挥重要作用，其变化水平可能对 MG 的诊断具有重要价值[13]。本组研究结果显示 MG 患者的 DBIL、IBIL、TBIL、UA 水平均显著低于健康对照人群，Evoli A[14]等的研究也得到了相似的结论。当机体 BIL 和 UA 水平降低时，抗氧化能力就会受到极大影响，此时机体免疫就会失衡，机体免疫的下降极易导致 MG 的发生；同时，MG 患者机体免疫失衡会逐渐加剧，氧化程度逐渐升高，在对抗逐渐加剧的机体氧化过程中，BIL 和 UA 会被大量消耗，进而进一步使血清 BIL 和 UA 水平降低[15]。在对不同分型的 MG 进行比较后，我们发现不同类型 MG 患者血清 BIL 和 UA 水平并无显著差异。尽管对于这种机体损伤程度与监测指标不相匹配的情况现象我们可以找到类似的情况[16]，并且也有许多学者得出类似结论，但学界对其原因仍不十分清楚，还有待进一步探讨。

考虑到 MG 的发病率有较大的性别及年龄差异，我们还对不同性别及年龄层的患者及健康人血清 BIL 和 UA 水平进行了比较，发现无论 MG 患者还是健康对照人群，男性血清 DBIL、IBIL、TBIL、UA 值均明显高于同组女性，这也与既往 Huang X[17]等的研究结论一致。有研究认为不同性别人群体内激素水平有着显著差异，对血清 BIL 和 UA 水平会产生一定影响，同时不同的生活习惯、饮食习惯、情绪因素等都会对机体免疫及抗氧化能力产生极大影响[18,19]。这可能是导致本实验中性别对观察结果影响的原因。同时，也极有可能是引起 MG 女性发病率高于男性的重要原因[20]。但通过我们的另一项统计结果可以看出不同年龄层的患者血清 BIL 和 UA 值并未具有统计学差异，这不能解释为何儿童 MG 发病率高于成人，因此还需要进行进一步的深入探讨。

综上所述，血清 BIL 和 UA 与重症肌无力患者的疾病发生存在着一定的联系，检测血清 BIL 和 UA 水平对重症肌无力患者的诊断有一定的参考价值。

参 考 文 献 (References)

[1] PasnoorM, Wolfe GI, Nations S, et al. Clinical findings in MuSK-antibody positive myasthenia gravis: a U. S. experience[J]. Muscle Nerve, 2010, 41(3): 370-374
[2] Pouwels S, de Boer A, Javaid MK, et al. Fracture rate in patients with myasthenia gravis:the general practice research database [J]. Osteoporosis International, 2013, 24(2): 467-476
[3] Kohler S, Keil TOP, Swierzy M, et al. Disturbed B cell subpopulations and increased plasma cells in myasthenia gravis patients[J]. Journal of neuroimmunology, 2013, 264(1): 114-119
[4] Breimer LH, Mikhailidis DP. Is bilinlbin a marker of vascular disease and / or cancer and is it a potential therapeutic tareet?[J]. CuH PhaHn Des, 2011, 17: 3644-3655
[5] Lavmic D, Basta I, Rakocevic-stojanovic V, et a1. Epidemiological Study of Adult-0nset Myasthenia Gravis in the Area of Belgrade(Serbia) in the Period 1979-2008 [J]. Neuro epidemiology, 2013, 40: 190-194
[6] Liu B, Shen Y, Xiao K, et aI. Semm uric acid leveIs in patients with multiple sclemsis：a meta analysis[J]. Neuml Res, 2012, 34: 163-171
[7] Fuhua P, Xuhui D, Zhiyang Z, el a1. Ancioxidant status of bilirubin and uric acid in patients with mvasthenia gravis [J]. Neumimmunomodulation, 2012, 19: 4349
[8] Dalakas M C. Novel future therapeutic options in myasthenia gravis[J]. Autoimmunity Reviews, 2013, 12(9): 936-941
[9] Huijbers MG, Zhang W, Klooster R, et al. MuSK IgG4 auto antibodies cause myasthenia gravis by inhibiting binding between MuSK and Lrp4[J]. Proceedings of the National Academy of Sciences, 2013, 110 (51): 20783-20788
[10] Cheng Z, Qiu S, Jiang L, et al. MiR-320a is down regulated in patients with myasthenia gravis and modulates inflammatory cytokines production by targeting mitogen-activated protein kinase 1[J]. Journal of Clinical Immunology, 2013, 33(3): 567-576
[11] Sieb J P. Myasthenia Gravis: An Update for the Clinician[J]. Clinical & Experimental Immunology, 2013, 175(3): 408-418
[12] Berrih-Aknin S, Frenkian-Cuvelier M, Eymard B. Diagnostic and clinical classification of autoimmune myasthenia gravis[J]. Journal of Autoimmunity, 2014, 48-49(2): 143-148
[13] Cavalcante P, Cufi P, Mantegazza R, et al. Etiology of myasthenia gravis: innate immunity signature in pathological thymus[J]. Autoimmunity Reviews, 2013, 12(9): 863-874
[14] Evoli A, Padua L. Diagnosis and therapy of myasthenia gravis with antibodies to muscle-specific kinase [J]. Autoimmunity Reviews, 2013, 12(9):931-935
[15] Cristian M I, Acsadi G. Management of Juvenile Myasthenia Gravis [J]. Pediatric Neurology, 2013, 48(2): 95-104
[16] Fekih-Mrissa N, Klai S, Zaouali J, et al. Association of HLA-DR/DQ polymorphism with myasthenia gravis in Tunisian patients [J]. Clin Neurol Neurosurg, 2013, 115(1): 32-36
(下转第 6914 页)

(C)1994-2023 China Academic Journal Electronic Publishing House. All rights reserved. http://www.cnki.net

[6] Gundlach KK. Ankylosis of the temporomandibular joint[J]. Journal of Cranio-Maxillofacial Surgery, 2010, 15(02): 122-130

[7] Brenkert DR. Orthodontic treatment for the TMJ patient fol lowing splint therapy to stabilize a displaced disk (s): A systemized approach. Part Ⅰ, TMJ orthodontic diagnosis [J]. Cranio-the Journal of craniomandibular practice, 2010, 21(03): 193-199

[8] Molina F. Mandibular distraction osteogenesis:a clinical experience of the last 17 years[J]. Journal of Cranio-Maxillofacial Surgery, 2009, 39 (Suppl 2): 1794-1800

[9] Ringold S, Cron RQ. The temporomandibular joint in juvenile idiopathic arthritis:frequently used and frequently arthritic [J]. Pediatr Rheumatol Online J, 2009, 7(01): 11

[10] Yu H, Yang X, Cheng J. Distraction osteogenesis combined with tissue-engineered cartilage in the reconstruction of condylar osteochondral defect [J]. Journal of Oral and Maxillofacial Surgery, 2011, 14 (12): e558-e564

[11] Yu H, Wang X, Fang B. Comparative study of different osteotomy modalities in maxillary distraction osteogenesis for cleft lip and palate [J]. Journal of Oral and Maxillofacial Surgery, 2012, 13 (11): 2641-2647

[12] Yang D, Yu H, Cheng AH. Sequential maxillary bifocal transport distraction osteogenesis after radiotherapy[J]. Journal of Craniofacial Surgery, 2011, 26(02): 742-745

[13] Cai M, Lu X, Shen G. Customized bifocal and trifocal transport distraction osteogenesis device for extensive mandibular reconstruction [J]. Journal of Craniofacial Surgery, 2011, 19(02): 562-565

[14] Yu H, Cheng J, Cheng AH. Preliminary study of virtual orthognathic surgical simulation and training [J]. Journal of Craniofacial Surgery, 2011, 24(02): 648-651

[15] Liang X, Jacobs R, Hassan B. A comparative evaluation of Cone Beam Computed Tomography (CBCT) and Multi-Slice CT (MSCT) Part Ⅰ. On subjective image quality [J]. European Journal of Radiology, 2010, 21(02): 265-269

[16] Lorenzoni DC, Bolognese AM, Garib DG. Cone-beam computed tomography and radiographs in dentistry: aspects related to radiation dose[J]. Int J Dent, 2012, 18(09): 233-234

[17] Cevidanes LHS, Hajati AK. Quantification of condylar resorption in temporomandibular joint osteoarthritis [J]. Oral Surgery, Oral Medicine, Oral Pathology, Oral Radiology & Endodontics, 2010, 20 (01): 110-117

[18] Wiese M, Svensson P, Bakke M. Association between temporomandibular joint symptoms, signs, and clinical diagnosis using the RDC/TMD and radiographic findings in temporomandibular joint tomograms[J]. Journal of Orofacial Pain, 2008, 33(03): 239-25l

[19] Ghoneima AA, Al am ES, Zunt SL. Bisphosphonates treatment and orthodontic considerations [J]. Orthod Craniofac Res, 2010, 18(01): 1-10

[20] Brusveen EM, Brudvik P, Bøe OE. Apical root resorption of incisors after orthodontic treatment of impacted maxil ary canines: a radiographic study[J]. {H}American Journal of Orthodontics and Dentofacial Orthopedics, 2012, 22(04): 427-435

(上接第 6859 页)

[17] Huang X, Liu W B, Men L N, et al. Clinical features of myasthenia gravis in southern China: a retrospective review of 2,154 cases over 22 years[J]. Neurological Sciences, 2013, 34(6): 911-917

[18] Zhang M, Zhou Y, Guo J, et al. Thymic TFH cells involved in the pathogenesis of myasthenia gravis with thymoma [J]. Experimental Neurology, 2014, 254(4): 200-205

[19] Mineo T C, Ambrogi V. Outcomes after thymectomy in class I myasthenia gravis[J]. Journal of Thoracic & Cardiovascular Surgery, 2013, 145(5): 1319-1324

[20] Chu J, Han B, Ying D. Robotic-assisted Extended Thymectomy for Myasthenia Gravis in Elderly Patients [J]. Chinese Journal of Minimally Invasive Surgery, 2013, 13(1): 9-29

(C)1994-2023 China Academic Journal Electronic Publishing House. All rights reserved. http://www.cnki.net

中图分类号：R746.1 学校代码：10660
UDC：616.8 学　　号：2019120020232
学科专业代码：105104 密　　级：公开

貴州醫科大學

2022 届硕士学位论文

（专业学位）

重症肌无力患者血清尿酸、胆红素、白蛋白水平变化的临床分析

Clinical Analysis of Changes in Serum Uric Acid, Bilirubin and Albumin Levels in Patients with Myasthenia Gravis

研 究 生：刘 磊
指导教师：张艺凡　主任医师
年　　级：2019 级
专　　业：神经病学
提交日期：二〇二二年六月

中国知网 https://www.cnki.net

中图分类号：R746.1
UDC：616.8
学科专业代码：105104
学校代码：10660
学　　号：2019120020232
密　　级：公开

贵州医科大学

2022 届 硕 士 学 位 论 文

（专业学位）

重症肌无力患者血清尿酸、胆红素、白蛋白水平变化的临床分析

Clinical Analysis of Changes in Serum Uric Acid, Bilirubin and Albumin Levels in Patients with Myasthenia Gravis

论文作者：刘磊
指导教师：张艺凡 主任医师
申请学位：医学硕士
培养单位：贵州医科大学临床医学院
学科专业：神经病学
研究方向：神经免疫疾病
研究起止日期：2019年7月至2022年3月
答辩委员会主席：商慧芳 教授
论文答辩日期：2022年6月1日

中国知网　https://www.cnki.net

中国知网 https://www.cnki.net

目　录

中文摘要 …………………………………………………… I
英文摘要 …………………………………………………… II
略缩词表 …………………………………………………… III
前言 ………………………………………………………… 1
1 材料与方法 ……………………………………………… 3
2 结果………………………………………………………4
3 讨论 ……………………………………………………… 11
4 结论 ……………………………………………………… 17
参考文献 …………………………………………………… 18
综述 ………………………………………………………… 23
作者简介及攻读学位期间科研成果………………………31
致谢 ………………………………………………………… 32
学位论文数据集 …………………………………………… 33

中国知网 https://www.cnki.net

重症肌无力患者血清尿酸、胆红素、白蛋白水平变化的临床分析

专业：神经病学　　学号：2019120020232　研究生：刘磊

导师：张艺凡

摘要

目的：

通过分析重症肌无力（MG）患者血清内源性抗氧化剂尿酸（UA）、胆红素（BIL）、白蛋白（ALB）的水平变化，进一步探讨MG患者血清抗氧化水平与MG的相关性，为临床诊治提供依据。

方法：

选取2016年1月至2021年12月于贵州医科大学附属医院就诊的MG患者共221例，同时选取同期性别、年龄比相近的健康体检者共198例，收集所有研究对象的临床资料及血清尿酸、总胆红素（TBIL）、直接胆红素（DBIL）、间接胆红素（IBIL）、白蛋白、尿素（Urea）、肌酐（Crea）、谷丙转氨酶（ALT）、谷草转氨酶（AST）等实验室检查资料，收集69例随访患者的QMG及MGFA-PIS评分信息，分别对不同性别、有无胸腺瘤、不同疾病分型MG患者的各项指标进行分析，并分析MG患者年龄、病程、BMI与血清尿酸、胆红素、白蛋白的相关性，进一步分析了随访患者治疗前后的血清尿酸、胆红素、白蛋白变化及其与QMGS的相关性。

结果：

1.MG组与HC组相比，男女比例、年龄、BMI、Urea、Crea、ALT、AST等均无明显差异（$P>0.05$），MG组分别按照有无胸腺瘤、不同疾病分型比较，上述指标之间均无明显差异（$P>0.05$）。

2.MG患者血清UA、TBIL、DBIL、IBIL、ALB水平[(298.75±83.87)

中国知网　https://www.cnki.net

μmol/L,(11.21±3.28) μmol/L,(3.72±1.19) μmol/L,(7.49±2.35) μmol/L,(42.29±5.97) g/L]显著低于健康对照组[(328.63±62.48)μmol/L，(13.12±3.69)μmol/L，(4.50±1.24)μmol/L，(8.62±2.68)μmol/L，(44.67±3.23)g/L]，差异具有统计学意义（P<0.001）。

3. MG组男性血清UA、TBIL、DBIL、ALB水平较HC组男性降低（P<0.05），IBIL无明显差异（P=0.074）；MG组女性血清UA、TBIL、DBIL、IBIL、ALB较HC组均明显降低（P<0.05）；MG患者组间，女性患者血清UA、TBIL、DBIL、IBIL、ALB较男性均明显下降（P<0.01）。

4.有无胸腺瘤MG患者间血清UA、TBIL、DBIL、IBIL、ALB水平均无明显差异（P>0.05）。

5.全身型MG患者中，不论男女血清UA、TBIL、DBIL、IBIL、ALB水平均较眼肌型MG患者低，差异具有统计学意义（P<0.05）。

6.MG患者血清TBIL、DBIL、IBIL、ALB与年龄、病程、BMI无相关性（P>0.05），血清UA水平与病程呈负相关（P=0.015），与年龄、BMI无相关性。

7.在经治疗后患者QMG评分明显下降（P<0.001），经治疗后患者UA、TBIL、IBIL、ALB水平均有所升高（P<0.05）。

8.随访组患者治疗前血清UA、TBIL、IBIL、ALB水平与QMG评分呈负相关（P<0.05），治疗后患者血清UA、ALB与QMG评分呈负相关（P<0.05）。

结论:

1.MG女性患者血清UA、TBIL、DBIL、IBIL、ALB水平均低于男性患者，可能是女性较男性更易患MG的原因之一，女性比男性更易患全身型MG，眼肌型MG患者的血清抗氧化水平高于全身型MG患者，血清UA、BIL、ALB水平下降及女性性别可能是导致OMG转化为GMG的危险因素之一。

2.有无胸腺瘤MG患者之间的血清UA、BIL、ALB水平无明显差异，可能是由于胸腺瘤相关MG的发病机制不同以及胸腺瘤复杂的免疫机制及凋亡机制导致的。

3.血清UA、BIL、ALB水平或许可以作为评价MG患者急性期疾病严重程度的一个客观指标，其有望成为监测MG疾病发展、评估治疗疗效的生物标志物。

中国知网 https://www.cnki.net

关键词：

重症肌无力；尿酸；胆红素；白蛋白；氧化应激

中国知网 https://www.cnki.net

Clinical Analysis of Changes in Serum Uric Acid, Bilirubin and Albumin Levels in Patients with Myasthenia Gravis

ABSTRACT

Major: Neurology Student ID：2019120020232 Candidate: Liu Lei

Supervisor: Zhang Yifan

Objective：

By analyzing the changes in the levels of serum endogenous antioxidants uric acid (UA), bilirubin (BIL) and albumin (ALB) in patients with myasthenia gravis (MG), the correlation between serum antioxidant levels and MG in MG patients is further explored to provide a basis for clinical diagnosis and treatment.

Methods：

A total of 221 MG patients attending the Affiliated Hospital of Guizhou Medical University from January 2016 to December 2021 were selected, along with a total of 198 health checkups with similar sex and age ratios during the same period. Clinical data and laboratory test information such as serum uric acid, total bilirubin (TBIL), direct bilirubin (DBIL), indirect bilirubin (IBIL), albumin, urea, creatinine (Crea), alanine aminotransferase (ALT), and aspartate aminotransferase (AST) were collected from all study subjects. The information of QMG and MGFA-PIS scores of 69 follow-up patients were collected to analyze indicators of MG patients with different sexes, with or without thymoma, and different disease subtypes separately, as well as the correlation between ages, course of disease, BMI and serum uric acid, bilirubin and albumin of MG patients, and to further analyze the changes of serum uric acid, bilirubin and albumin before and after treatment and their correlation with QMGS of follow-up patients.

Results:

1. There was no significant difference between the MG group and the HC group in terms of

中国知网 https://www.cnki.net

male to female ratio, age, BMI, Urea, Crea, ALT and AST ($P > 0.05$), and there was no significant difference between the above indicators of the MG group according to the presence or absence of thymoma and different disease subtypes, respectively ($P > 0.05$).

2. The serum levels of UA, TBIL, DBIL, IBIL, and ALB [(298.75±83.87) μmol/L, (11.21±3.28) μmol/L, (3.72±1.19) μmol/L, (7.49±2.35) μmol/L, (42.29±5.97) g/L] were significantly lower in MG patients than those in the HC group [(328.63±62.48) μmol/L, (13.12±3.69) μmol/L, (4.50±1.24) μmol/L, (8.62±2.68) μmol/L, (44.67±3.23) g/L], the difference between the two groups has statistically significance (P<0.001).

3. In the MG group, the serum levels of UA, TBIL, DBIL, and ALB in men were lower than those in male in HC group ($P < 0.05$), but there was no significant difference in IBIL ($P = 0.074$); in the MG group, the serum levels of UA, TBIL, DBIL, IBIL, and ALB of female were significantly lower than those of the HC group (P<0.05); among the MG patient groups, the serum levels of UA, TBIL, DBIL, IBIL, and ALB in female patients were significantly lower than those in male patients (P<0.01).

4. There was no significant difference in serum levels of UA, TBIL, DBIL, IBIL, and ALB between MG patients with and without thymoma ($P > 0.05$).

5. In the patients with systemic MG, the serum levels of UA, TBIL, DBIL, IBIL, and ALB in both men and women were lower than those in the patients with ophthalmoplegia MG, and the difference was statistically significant (P<0.05).

6. The serum levels of TBIL, DBIL, IBIL and ALB in MG patients had no correlation with age, course of disease and BMI (P>0.05), and serum UA level was negatively correlated with course of disease (P=0.015), but not with age and BMI.

7. After treatment, the QMG score of patients was significantly decreased (P<0.001), and the serum levels of UA, TBIL, IBIL, and ALB were increased after treatment (P<0.05).

8. In the follow-up patients, the serum levels of UA, TBIL, IBIL and ALB before treatment were negatively correlated with QMG score ($P < 0.05$), and the serum levels of UA, and ALB were negatively correlated with QMG score after treatment ($P < 0.05$).

Conclusions:

中国知网 https://www.cnki.net

1.The serum UA、TBIL、DBIL、IBIL、ALB level in female patients with MG is lower than that in male patients, which may be one of the reasons why women are more prone to MG than men. Female are more likely to suffer from generalized MG than male. The serum antioxidant level of patients with ocular MG is greater than that of generalized MG. The decrease of serum levels of UA, BIL and ALB as well as female gender may be one of the risk factors leading to the conversion of ocular MG to generalized MG.

2. There was no significant difference in serum levels of UA, BIL, and ALB between MG patients with and without thymoma, which may be due to the different pathogenesis of thymoma-related MG and the complex immune mechanism and apoptosis mechanism of thymoma.

3. Serum UA, BIL, and ALB levels may be an objective indicator to evaluate the severity of disease in the acute phase in patients with MG, and are expected to be biomarkers for monitoring MG disease progression and assessing therapeutic effect.

Key Words:

myasthenia gravis; Uric acid; Bilirubin; Albumin; Oxidative stress

中国知网 https://www.cnki.net

缩略词表

缩略词	英文全称	中文全称
AchR	acetylcholine receptor	乙酰胆碱受体
ALB	albumin	白蛋白
BIL	bilirubin	胆红素
GMG	generalized myasthenia gravis	全身型重症肌无力
MG	myasthenia gravis	重症肌无力
NT-MG	nonthymomatous myasthenia gravis	非胸腺瘤相关重症肌无力
OMG	ocular myasthenia gravis	眼肌型重症肌无力
OS	oxidative stress	氧化应激
QMGS	quantitive MG score	MG 定量评分
ROS	reactive oxygen species	活性氧
T-MG	thymomatous myasthenia gravis	胸腺瘤相关重症肌无力
UA	uric acid	尿酸

中国知网 https://www.cnki.net

重症肌无力患者血清尿酸、胆红素、白蛋白水平变化的临床分析

前 言

重症肌无力（Myasthenia Gravis，MG）是影响骨骼肌神经肌肉接头(Neuromuscular Junction,NMJ) 的最常见疾病，其是由于自身抗体的产生，导致神经-肌肉接头突触后膜处主要以乙酰胆碱受体（Acetylcholine Receptor,AchR）减少所引起的自身免疫性疾病[1] 。MG的症状范围包括单纯的眼部症状到肢体、延髓和呼吸肌的严重无力，症状的晨轻暮重和波动性是其典型表现。有研究表明，MG的患病率约为（150-200人）/100万人，并且由于对疾病的识别、诊断和治疗的改进，这一数字还在逐步上升[2]。MG可根据累及肌群和严重程度、血清抗体类型、发病年龄以及胸腺病理类型等分为多种类型[3,4]：（1）美国重症肌无力基金会 (Myasthenia Gravis Foundation of America,MGFA)临床分型，其中I型称为眼肌型MG（Ocular Myasthenia Gravis,OMG），其他累及眼肌外任一肌群的统称为全身型MG（Generalized Myasthenia Gravis,GMG）；（2）根据血清抗体类型可分为：抗AchR型MG、抗肌肉特异性受体酪氨酸激酶(MuSK)型MG、抗低密度脂蛋白受体相关蛋白 4 (LRP4)型MG、抗肌联蛋白（Titin）型MG以及血清阴性MG。（3）根据有无胸腺瘤分为：胸腺瘤相关性MG（Thymomatous Myasthenia Gravis，T-MG）及非胸腺瘤相关性MG（Nonthymomatous Myasthenia Gravis，NT-MG）。（4）根据发病年龄可分为：早发型MG(Early-onset Myasthenia Gravis，EOMG)：首次发病在50岁之前；晚发型MG(Late-onset Myasthenia Gravis，LOMG)：首次发病在50岁之后。近年来，氧化应激（Oxidative Stress，OS）已被证实参与了动脉粥样硬化、糖尿病和癌症等多种疾病的发展，其在自身免疫及神经退行性疾病中的作用也越来越受到关注。机体在氧化应激过程中将产生大量活性氧（Reactive Oxygen Species,ROS），大量的ROS会增加炎症活动并激活免疫细胞，已有多项研究表明氧化应激参与了多发性硬化（Multiple Sclerosis,MS）、系统性红斑狼疮（Systemic Lupus Erythematosus,SLE）及类风湿性关节炎（Rheumatoid Arthritis,RA）等自身免疫病的疾病活动[5,6]。在MG中，也有研究表明MG患者体内存在活跃的氧化应激状态[7]，并且ROS的产生会对AchR造成损伤[8]。在人类机

中国知网 https://www.cnki.net

体存在着多种内源性抗氧化系统来对抗氧化应激导致的损伤，胆红素（Bilirubin，BIL）由血红素加氧酶和胆绿素还原酶催化形成，是血色素的终产物，具有抗炎及抗氧化活性[9]。尿酸（Uric Acid，UA）是嘌呤核苷酸代谢的最终产物，在人体内由黄嘌呤脱氢酶合成，约占血浆中抗氧化能力的60%[10]。人血清白蛋白(Albumin，ALB)是血浆中含量最丰富的蛋白质，在防止氧化损伤中起重要作用[11]。本文通过研究血清UA、BIL、ALB水平与MG的关系，进一步探讨MG的发病机理，并为MG的发病机制和治疗提供思路。

中国知网 https://www.cnki.net

1 材料与方法

1.1 研究对象

研究对象选取2016年1月至2021年12月于我院门诊或住院部就诊的新发或稳定后复发重症肌无力患者，共收集221例MG患者，其中男79例，女142例（男:女为1：1.80），共69例患者有完整的随访信息，其中男22例，女47例（男：女为1：2.14）。健康对照组选取同期于我院体检中心体检的年龄、BMI相近的健康人员共198例，其中男70例，女128例（男:女为1：1.83）。

1.1.1 纳入标准：

（1）病例组需符合《中国重症肌无力诊断和治疗指南(2015版)》诊断标准[12]；

（2）患者均处于急性期；

（3）随访患者需达到具有至少两次完整的QMGS记录及相应的实验室指标，且随访中患者症状有所减轻或恢复。

1.1.2 排除标准：

（1）患者同时或既往患有任何原因的肝功能异常、肾衰竭病史、糖尿病、痛风、高尿酸血症、胆道疾病、感染性疾病、其他自身免疫病。

（2）患者近期或目前正在使用任何甾体类药物及任何除治疗MG的药物外可能导致尿酸、胆红素升高药物的患者。

1.2 方法

1.2.1 临床资料收集

收集病例信息包括：性别、年龄、病程、用药情况、既往史、BMI、胸腺情况、MGFA分型、MG定量评分 (Quantitive MG Score,QMGS)，美国MG协会干预后分型量表(Myasthenia Gravis Foundation of America Postintervention Status，MGFA-PIS)。QMGS是常用的客观评价MG患者各肌群无力程度的标准化量表，临床应用广泛，对评估MG患者症状严重程度具有良好的可靠性[13]，评估指标包含眼肌、面肌、延髓肌、颈肌、四肢肌、呼吸肌等共13个条目，每条根据肌群无力程度分为正常（0分）、轻度（1分）、中度（2分）、重度（3分），共计39分，分值越高代表患者肌无力越严重。MGFA-PIS旨在评估MG患者在治疗后的临床状态，该量表将MG治疗后状态定义为完全稳定缓解（CSR）、药理缓解（PR）、最小表现（MM）、改良（I）、无改变（U）、加重（W）、恶化（E）、死亡（D of MG）

中国知网 https://www.cnki.net

[14]，本研究将达到CSR、PR、MM、I评分等级的患者视为治疗有效。

1.2.2 病例分组

将收集的所有MG患者作为MG组，根据患者有无胸腺瘤分为胸腺瘤相关MG（T-MG）组及非胸腺瘤相关MG（NT-MG）组；根据患者累及肌群分为眼肌型MG（OMG）组及全身型MG（GMG）组；将收集的具有完整随访信息的患者作为随访组；将收集的所有健康人作为对照组（HC组）。

1.2.3 实验室指标收集

所有受试者均于门诊就诊或入院当天或次日收集空腹外周静脉血3-5ml，检验项目包括：血清尿素（Urea）、肌酐（Crea）、尿酸（UA）、丙氨酸氨基转移酶(ALT)、天门冬氨酸氨基转移酶（AST）、总胆红素（TBIL）、直接胆红素（DBIL）、间接胆红素（IBIL）、白蛋白（ALB）浓度，所有数据均来自贵州医科大学附属医院临床检验中心。

1.3 统计学方法

使用SPSS26.0软件进行数据分析处理。所有计量资料先进行正态分布检验，如果各组均满足正态性，采用均数±标准差进行统计描述，采用T检验进行组间比较；否则采用中位数（四分位数间距）进行统计描述，采用非参数检验进行组间比较，$P<0.05$认为两组间具有显著差异。对于可进行相关分析的数据采用spearman或pearson线性相关性分析，其中 $P<0.05$ 差异有统计学意义。

2 结 果

2.1 一般临床资料

221例MG患者共79例男性，142例女性（男:女为1：1.80），平均年龄48.55±16.79岁，平均BMI为22.46±3.21kg/m^2，病程中位数为4月；HC组共198名，其中70例男性，128例女性（男:女为1：1.83），平均年龄48.35±16.65岁，平均BMI为22.58±3.34kg/m^2。两组间性别比、年龄、BMI、Urea、Crea、ALT、AST均无显著差异。MG组中共41名患者患有胸腺瘤，其中15例男性，26例女性（男:女为1：1.73），平均年龄51.10±15.34岁，平均BMI为21.98±2.73kg/m^2,125名患者未发现胸腺瘤，其中45例男性，80例女性（男:女为1：1.78），平均年龄48.64±17.46 岁，平均BMI为22.78±3.47kg/m^2,两组间无明显差异，其余患者为胸腺增生、胸腺退化

中国知网 https://www.cnki.net

表1 所选研究对象的一般临床资料

Tab. 1 General clinical information of the selected study subjects

	例数（男/女）	男女之比	平均年龄（岁, $\bar{x}\pm s$）	BMI（kg/m², $\bar{x}\pm s$）	病程[月, M(P25,P75)]	Urea [mmol/L, $\bar{x}\pm s$]	Crea（μmol/L, $\bar{x}\pm s$）	ALT [U/L, $\bar{x}\pm s$]	AST [U/L, $\bar{x}\pm s$]
MG组	221（79/142）	1/1.80	48.55±16.79	22.46±3.21	4（1,24）	4.90±1.49	66.39±17.78	20.12±9.49	20.42±6.67
T-MG组	41（15/26）	1/1.73[a]	51.10±15.34[a]	21.98±2.73[a]	4（0.85,30）[a]	5.06±1.51[a]	63.47±16.49[a]	18.57±7.95[a]	20.70±6.11[a]
NT-MG组	125（45/80）	1/1.78	48.64±17.46	22.78±3.47	4（0.67,24）	5.00±1.51	67.57±17.72	20.68±9.44	20.43±7.14
OMG组	85（36/49）	1/1.36[b]	50.12±16.64[b]	22.23±3.26[b]	4（1,24）[b]	4.80±1.34[b]	63.47±15.51[b]	21.32±9.78[b]	21.10±6.74[b]
GMG组	136（43/93）	1/2.16	47.53±16.87	22.61±3.19	5（1,24）	4.96±1.58	68.21±18.90	19.37±9.27	20.00±6.62
随访组	69（22/47）	1/2.14	40.30±17.76	22.81±3.88	5（1,24）	4.78±1.38	58.77±14.84	19.61±10.59	18.45±5.71
HC组	198（70/128）	1/1.83	48.35±16.65	22.58±3.34	-	4.92±1.27	68.54±17.44	19.36±9.08	20.36±5.50
*P*值		0.933	0.946	0.703	-	0.907	0.213	0.403	0.917

注：*P*值：MG组与HC组相比；[a]:与NT-MG组相比，$P>0.05$；[b]:与GMG组相比，$P>0.05$。

中国知网 https://www.cnki.net

不全、胸腺残留或无相关检查结果；OMG患者共85名，其中36例男性，49例女性（男:女为1：1.36），平均年龄50.12±16.64岁，平均BMI为22.23±3.26 kg/m^2,GMG患者共136名，其中43例男性，93例女性（男:女为1：2.16），平均年龄47.53±16.87岁, 平均BMI为22.61±3.19kg/m^2，两组间OMG组男女比例大于GMG组，但无明显统计学差异（P=0.105），其余各指标之间均无显著差异；69例随访患者共22例男性，47例女性（男：女为1：2.14），平均年龄40.30±17.76岁，平均BMI为22.81±3.88kg/m^2，病程中位数为5月。（表1）

2.2 MG组与HC组血清UA、TBIL、DBIL、IBIL、ALB水平比较

MG组血清UA为（298.75±83.87）μmol/L，TBIL为（11.21±3.28）μmol/L，DBIL为（3.72±1.19）μmol/L，IBIL为（7.49±2.35）μmol/L，ALB为（42.29±5.97）g/L，较HC组明显降低，差异具有统计学意义（P＜0.001)。（表2）（图1）

表2 MG组与HC组间血清UA、TBIL、DBIL、IBIL、ALB水平比较（$\bar{x}$±s）

Tab. 2 Comparison of serum UA, TBIL, DBIL, IBIL and ALB levels between the MG and HC groups（$\bar{x}$±s）

组别	UA （μmol/L）	TBIL （μmol/L）	DBIL （μmol/L）	IBIL （μmol/L）	ALB （g/L）
MG组	298.75±83.87	11.21±3.28	3.72±1.19	7.49±2.35	42.29±5.97
HC组	328.63±62.48	13.12±3.69	4.50±1.24	8.62±2.68	44.67±3.23
P值	＜0.001	＜0.001	＜0.001	＜0.001	＜0.001

中国知网 https://www.cnki.net

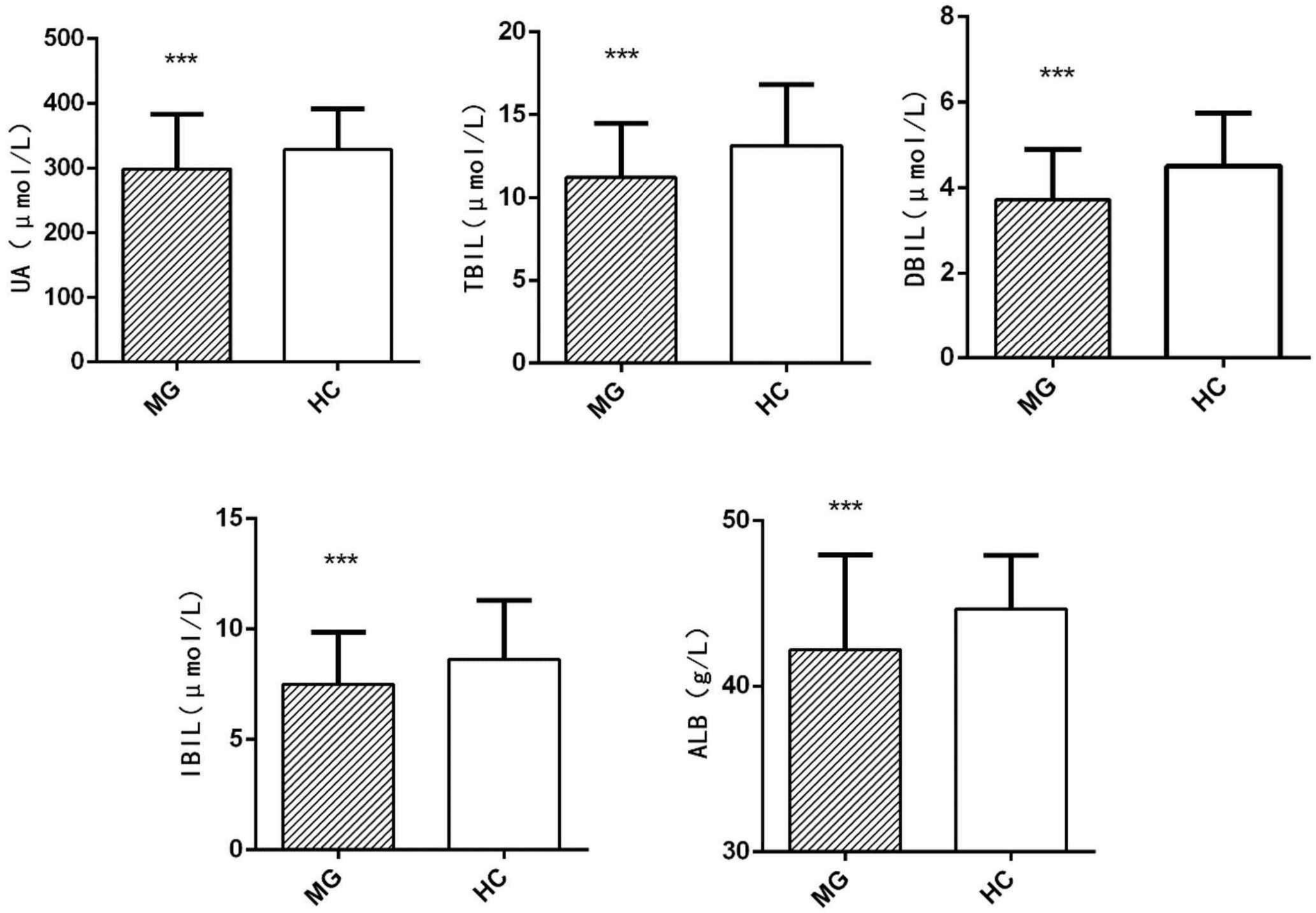

注：与 HC 组相比，***P＜0.001。

图 1 MG 组与 HC 组间血清 UA、TBIL、DBIL、IBIL、ALB 水平

Fig. 1 Serum UA, TBIL, DBIL, IBIL and ALB levels between the MG and HC groups

2.3 MG组与HC组不同性别之间血清UA、TBIL、DBIL、IBIL、ALB水平比较

MG组男性血清UA、TBIL、DBIL、ALB水平较HC组男性降低，差异具有统计学意义（P＜0.05），MG组男性IBIL较HC组男性有所下降，差异无统计学意义（P=0.074）；女性MG患者中，血清UA、TBIL、DBIL、IBIL、ALB较HC组均明显降低，差异具有统计学意义（P＜0.05）。MG患者组间，女性患者血清UA、TBIL、DBIL、IBIL、ALB较男性均明显下降，差异具有统计学意义（P＜0.01）。（表3）

中国知网 https://www.cnki.net

表3　MG组与HC组不同性别之间血清UA、TBIL、DBIL、IBIL、ALB水平比较（$\bar{x}\pm s$）

Tab. 3 Comparison of serum UA, TBIL, DBIL, IBIL, and ALB levels between MG and HC groups by gender（$\bar{x}\pm s$）

组别	例数（例）	UA（μmol/L）	TBIL（μmol/L）	DBIL（μmol/L）	IBIL（μmol/L）	ALB（g/L）
男性						
MG组	79	348.64±73.09$^{*\#\#\#}$	12.44±3.64$^{*\#\#\#}$	4.17±1.27$^{**\#\#\#}$	8.27±2.73$^{\#\#}$	46.55±4.43$^{**\#\#\#}$
HC组	70	372.73±49.07	13.71±3.16	4.76±1.21	8.94±2.18	44.76±3.18
女性						
MG组	142	270.86±75.99*	10.53±2.84***	3.47±1.06***	7.06±1.99***	39.80±4.91***
HC组	128	288.61±43.55	12.64±4.07	4.28±1.22	8.35±3.05	44.51±3.33

注：与HC组相比$^{*}P$＜0.05，$^{**}P$＜0.01,$^{***}P$＜0.001；MG组内男女间相比$^{\#\#}P$＜0.01，$^{\#\#\#}P$＜0.001。

2.4 T-MG组与NT-MG组间血清UA、TBIL、DBIL、IBIL、ALB水平比较

MG患者组中，共有41名患者患有胸腺瘤，125名患者无胸腺瘤，两组患者间血清UA、TBIL、DBIL、IBIL、ALB水平均无明显差异（P＞0.05）。（表4）

表4 T-MG组与NT-MG组间血清UA、TBIL、DBIL、IBIL、ALB水平比较（$\bar{x}\pm s$）

Tab. 4　Comparison of serum UA, TBIL, DBIL, IBIL and ALB levels between the T-MG and NT-MG groups（$\bar{x}\pm s$）

组别	UA（μmol/L）	TBIL（μmol/L）	DBIL（μmol/L）	IBIL（μmol/L）	ALB（g/L）
T-MG	291.86±76.84	10.83±3.90	3.77±1.50	7.06±2.71	42.20±5.25
NT-MG	297.16±79.40	11.23±3.31	3.66±1.16	7.57±2.38	42.05±5.84
P值	0.705	0.553	0.660	0.280	0.882

2.5 OMG组与GMG组间血清UA、TBIL、DBIL、IBIL、ALB水平比较

为排除 OMG 与 GMG 患者组间男女比例不同对指标的影响，分别比较了两组间男性及女性血清 UA、TBIL、DBIL、IBIL、ALB 水平的差异。男性患者中，OMG 组血清 UA、TBIL、DBIL、IBIL、ALB 水平较 GMG 组高，差异具有统计学意义（P＜0.05）。在女性 OMG 患者与 GMG 患者间，OMG 患者血清 UA、

中国知网　https://www.cnki.net

TBIL、DBIL、IBIL、ALB 水平均较 GMG 组高（P＜0.05），其中 UA、DBIL 水平显著高于 GMG 组（P＜0.001）。（表 5）

表5 OMG组与GMG组间血清UA、TBIL、DBIL、IBIL、ALB水平比较（$\bar{x}\pm s$）

Tab. 5 Comparison of serum UA, TBIL, DBIL, IBIL and ALB levels between the OMG and GMG groups（$\bar{x}\pm s$）

分组	例数（例）	UA（μmol/L）	TBIL（μmol/L）	DBIL（μmol/L）	IBIL（μmol/L）	ALB（g/L）
男性						
OMG组	36	367.82±78.10*	13.78±3.89**	4.63±1.31**	9.16±2.84**	48.09±4.64**
GMG组	43	332.58±65.26	11.32±3.04	3.79±1.11	7.53±2.42	45.26±3.85
女性						
OMG组	49	303.07±80.81$^{\#\#\#}$	11.56±3.04$^{\#\#}$	3.89±1.14$^{\#\#\#}$	7.67±2.12$^{\#\#}$	41.12±3.94$^{\#}$
GMG组	93	254.10±68.71	9.98±2.58	3.24±0.96	6.74±1.84	39.10±5.24

注：与男性GMG组相比，$^{*}P$＜0.05，$^{**}P$＜0.01；与女性GMG组相比，$^{\#}P$＜0.05，$^{\#\#}P$＜0.01，$^{\#\#\#}P$＜0.001。

2.6 MG组血清UA、TBIL、DBIL、IBIL、ALB与年龄、病程、BMI的相关性分析

MG患者血清TBIL、DBIL、IBIL、ALB与年龄、病程、BMI无相关性（P＞0.05），血清UA水平与病程呈负相关（r=-0.163），与年龄、BMI无相关性。（表6）

表6 MG组血清UA、TBIL、DBIL、IBIL、ALB与年龄、病程、BMI的相关性分析（P值）

Tab. 6 Correlation analysis of serum UA, TBIL, DBIL, IBIL and ALB with age, disease duration and BMI in MG group(P-value)

变量	UA	TBIL	DBIL	IBIL	ALB
年龄	0.149	0.422	0.642	0.376	0.740
病程	0.015	0.354	0.320	0.561	0.625
BMI	0.054	0.889	0.499	0.591	0.575

2.7 随访组治疗前后血清UA、TBIL、DBIL、IBIL、ALB水平及QMG评分比较

共69名随访患者至少达到了MGFA-PIS等级中的改良（I）等级，随访组治疗前后Urea、Crea、ALT、AST变化无明显差异（P＞0.05），在经治疗后患者QMG

中国知网 https://www.cnki.net

评分明显下降（*P*<0.001），血清UA、TBIL、DBIL、IBIL、ALB水平均有所升高，其中UA、TBIL、IBIL、ALB具有统计学意义（*P*＜0.05）（表7）（图2）。

表 7 随访组治疗后血清 UA、TBIL、DBIL、IBIL、ALB 水平及 QMG 评分比较（ $\bar{x}\pm s$）

Tab. 7 Comparison of serum UA, TBIL, DBIL, IBIL, ALB levels and QMG scores in follow-up group after treatment（ $\bar{x}\pm s$）

变量	治疗前	治疗后	*t*	*P*
Urea(mmol/L)	4.78±1.38	4.74±1.38	0.154	0.878
Crea(mmol/L)	58.77±14.84	60.28±13.53	-0.623	0.534
ALT(U/L)	19.61±10.59	19.40±13.87	-0.097	0.923
AST(U/L)	18.45±5.71	18.72±9.91	-0.199	0.842
UA(μmol/L)	278.54±82.68	308.92±86.35	-2.111	0.037
TBIL(μmol/L)	10.89±2.82	12.26±3.63	-2.472	0.015
DBIL(μmol/L)	3.73±1.26	4.10±1.48	-1.552	0.123
IBIL(μmol/L)	7.16±2.00	8.16±2.46	-2.640	0.009
ALB(g/L)	42.01±6.67	45.39±4.47	-3.490	<0.001
QMG 评分	8.01±4.11	3.32±2.89	7.765	<0.001

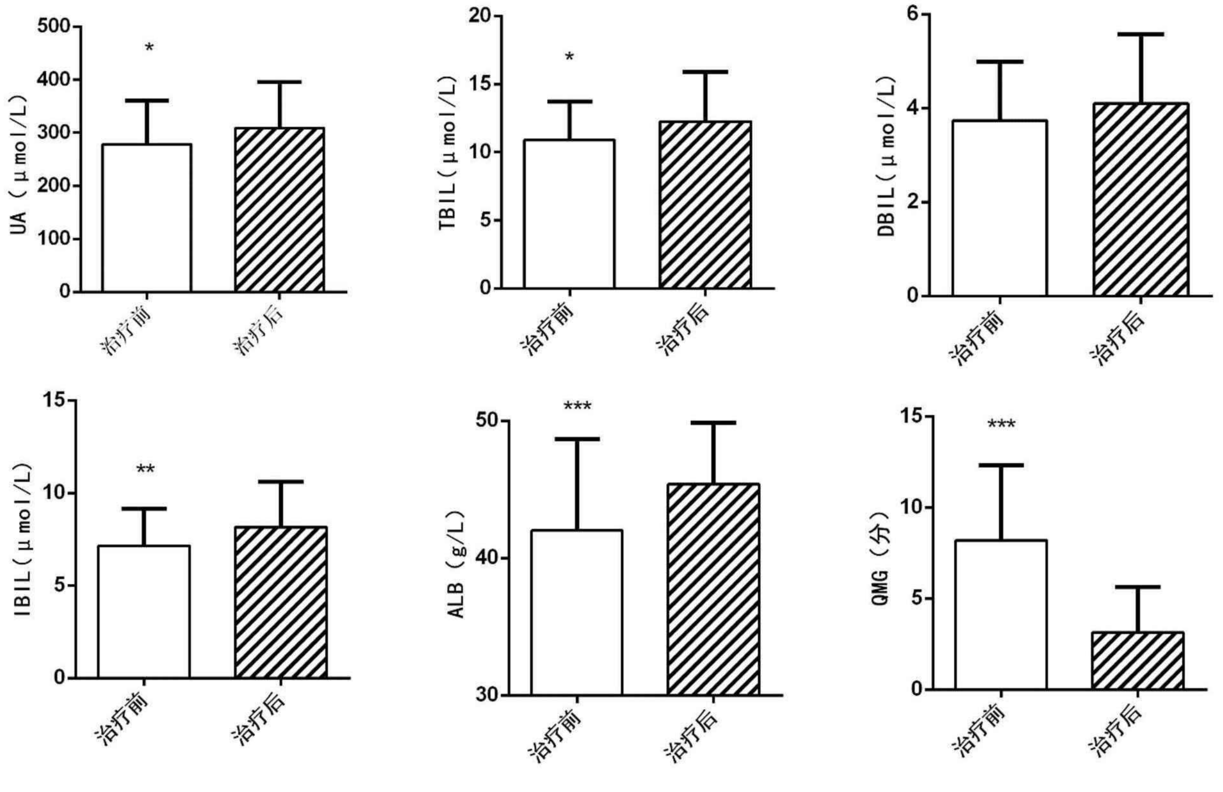

注：与治疗后相比，*P＜0.05，**P＜0.01，***P＜0.001

图 2 随访组治疗前后血清 UA、TBIL、DBIL、IBIL、ALB 水平及 QMG 评分

中国知网 https://www.cnki.net

Fig. 2 Serum UA, TBIL, DBIL, IBIL, ALB levels and QMG scores between the pretherapy and post-treatment in the follow-up group

2.8 随访组治疗前后QMG评分与血清UA、TBIL、DBIL、IBIL、ALB水平相关性分析

随访组患者治疗前血清UA、TBIL、IBIL、ALB水平与QMG评分呈显著相关（*P*=0.008，*P*=0.028，*P*=0.018，*P*=0.001），且QMG评分越高，血清UA、TBIL、IBIL、ALB水平越低（*r*=-0.318，*r*=-0.265，*r*=-0.283，*r*=-0.394）（表8）（图3）；治疗后患者血清UA、ALB与QMG评分相关（*P*=0.035，*P*=0.038），且两者呈负相关（*r*=-0.255，*r*=-0.251）（表8）。

表8 随访组治疗前后QMG评分与血清UA、TBIL、DBIL、IBIL、ALB水平相关性分析

Tab. 8 Correlation analysis of QMG score and serum UA, TBIL, DBIL, IBIL and ALB levels between the pretherapy and post-treatment in the follow-up group

QMG评分	UA	TBIL	DBIL	IBIL	ALB
治疗前					
P	0.008	0.028	0.231	0.018	0.001
r	-0.318	-0.265	-0.146	-0.283	-0.394
治疗后					
P	0.035	0.654	0.794	0.413	0.038
r	-0.255	-0.055	-0.032	-0.100	-0.251

3 讨 论

MG是由自身抗体介导、补体参与的自身免疫性疾病，在大多数MG患者体内可检测到针对肌肉烟碱型乙酰胆碱受体 (nAChR) 的自身抗体，T、B淋巴细胞亚群及细胞因子的失衡一直以来被认为是导致MG发生发展的重要影响因素[15]。近年来，ROS在自身免疫性疾病中的重要作用越来越受到重视，ROS是线粒体代谢和许多其他过程的副产物，当ROS升高并超过机体的抗氧化能力，导致氧化损伤和氧化修饰的过程被称为氧化应激，其也被认为是炎症反应、免疫耐受性崩溃和细胞凋亡的重要原因[16]。在对实验性自身免疫性重症肌无力（Experimental Autoimmune Myasthenia Gravis,EAMG）大鼠模型的骨骼肌基因分析研究中发现，Mt1e在所有骨骼肌中有所升高，而Mt1a是一种富含半胱氨酸的金属硫蛋白，可

中国知网 https://www.cnki.net

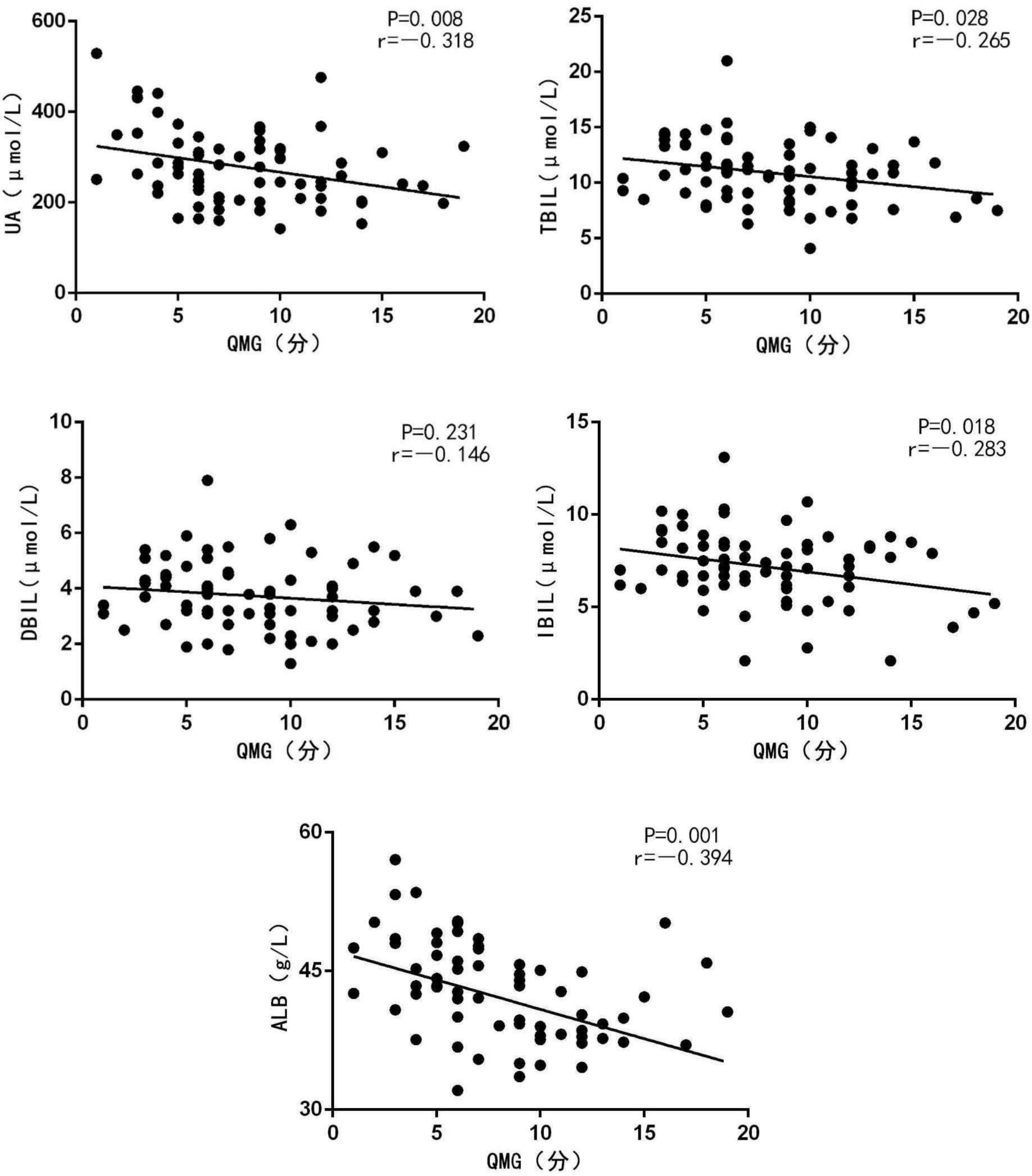

图 3 随访组治疗前血清 UA、TBIL、DBIL、IBIL、ALB 水平与 QMG 评分的线性相关

Fig.3 Linear correlation between pretherapy serum UA, TBIL, DBIL, IBIL, ALB levels and QMG scores in the follow-up group

导致细胞内活性氧水平升高，证明EAMG大鼠体内存在氧化应激状态[17]。此外，对MG患者的内源性抗氧化剂水平的研究也表明，MG患者体内的抗氧化水平下降，间接表明了MG患者存在高浓度的ROS水平[18]。人体内存在着内、外源性两

中国知网 https://www.cnki.net

种抗氧化防御系统，这两类抗氧化剂都能清除ROS和自由基[19]，尿酸、胆红素、白蛋白等因为其在人体内出色的抗氧化能力，近年来在MS、SLE、RA等自身免疫病中均有检测到降低。

UA存在于细胞、组织和器官中，具有很强的清除ROS的能力，其可通过清除过氧自由基和羟基自由基，或者通过结合其他催化自由基反应的金属离子来起到抗氧化剂的作用。从血浆中去除UA时，抗氧化能力下降了大约80%[20]，证明UA具有较强的抗氧化能力。此外，体外研究表明高水平的UA还能刺激单核细胞趋化蛋白-1、IL-1β、IL-6和INF-α等促炎分子的合成[21]，并且多项研究已表明痛风与RA、SLE及MS的共患率非常低[22,23]，证明UA与自身免疫性疾病的发生密切相关。胆红素是单线态氧的有效清除剂，得益于胆绿素-胆红素循环，IBIL可有效地保护神经元细胞免受高浓度的H_2O_2诱导的氧化应激损伤[24]，当细胞处于氧化应激状态下，细胞和组织中的胆红素水平上升，以防止过度的氧化应激损伤，还可预防各种氧化应激相关综合征[25]。除了抗氧化应激以外，BIL还可以抑制补体系统激活[26]、诱导效应性T细胞凋亡、促进Treg的增生[27]，均证实了BIL对免疫系统的调节作用。ALB含有一个单一的硫醇基团Cys34，约占人体血浆中还原硫醇的80%，Cys34具有高度的氧化还原特性，是血浆中多种活性氧和活性氮的清除剂，Anraku 等人[28]的研究表明，Cys34对ALB抗氧化活性的贡献水平分别占36%至68%不等。ALB也是血浆中最丰富的循环蛋白，其抗氧化活性源于其配体结合能力，除了可与游离金属离子结合，减少其ROS的产生外，ALB还可与半胱氨酸结合，减轻氧化损伤[29]，白蛋白还参与了胆红素的转运，间接提高了其抗氧化活性[30]。总之，以上研究表明了生理浓度的UA、BIL、ALB能够通过清除ROS、螯合产生自由基的物质等方式来保护细胞免受氧化应激介导的损伤，可充当氧化应激的生物标志物。

视神经脊髓炎（Neuromyelitis Optica,NMO）作为一种由水通道蛋白4（AQP4）的致病性自身抗体引起的自身免疫性疾病，其血清UA、TBIL、IBIL、ALB水平显著低于健康对照组[31]。在MS中也发现尿酸水平下降，并且与疾病活动相关[32]。在MG中，已有几项研究表明MG患者血清UA、BIL水平下降，Peng等人[33]的研究中发现MG患者的UA、TBIL、DBIL、IBIL水平显著低于健康对照组，且与疾病分型无关。周霞等人[34]比较了131名MG患者与健康人的UA、BIL水平，结果提示MG患者血清UA、TBIL、IBIL水平显著低于健康人，经校正影响因素后，

中国知网 https://www.cnki.net

低TBIL、低UA水平者患MG风险高于高TBIL、高UA水平者。其他的研究也有类似结论，不同的是在其他研究中，除了UA和BIL外，MG患者的血清ALB和Crea水平也有所降低[18]，并且UA水平与MG活动度呈负相关[35]。在本研究中，为排除年龄、BMI、肝肾功能异常对指标的影响，我们首先比较了MG患者与健康对照组的年龄、BMI、Urea、Crea、ALT、AST等指标，结果发现MG组的Crea较HC组有所降低，但差异无统计学意义（P=0.213），其余指标在两组间均无显著差异。在随后的研究中，我们发现MG患者血清UA、TBIL、DBIL、IBIL及ALB水平较HC组明显下降，这与之前的研究相符，而这些指标的下降提示了MG患者体内内源性抗氧化防御系统的受损，但其是否能充当MG发病的生物标志物尚需进一步研究。由于UA、BIL水平已被证明在男性中普遍高于女性[36,37]，因此本研究分别对男性及女性MG患者进行比较，发现女性MG患者的UA、TBIL、DBIL、IBIL及ALB水平均低于男性MG患者，且女性IBIL及ALB较男性下降更明显，这一结果与先前的研究相符，而更低的UA、BIL、ALB水平意味着机体更低的抗氧化应激防御能力，这似乎解释了女性更易患MG这一原因，但具体机制有待进一步探讨。不同的是，在我们比较不同性别的MG患者与健康对照组之间BIL的差异时，发现男性MG患者IBIL水平较HC组下降，但差异无统计学意义（P=0.074），这与之前的研究[34]不符，这些差异可能是由于样本量不同及不同地区患者的饮食习惯、生活方式的不同所导致的。

大约15%的MG患者患有胸腺瘤，胸腺瘤也因常合并自身免疫性疾病特别是MG而闻名。胸腺的生发中心是B细胞分化为产生抗体的细胞的部位，不论MG患者是否合并胸腺瘤，都推荐早期行胸腺切除术以缓解症状或减少复发[3]。有文献报道T-MG患者血清抗AchR抗体大多呈阳性[38]，MG合并胸腺瘤也意味着疾病严重程度越高以及预后较差[39]。本研究比较了T-MG以及NT-MG患者之间的血清UA、BIL、ALB水平的差异，意外的是两者之间均无明显差异，这与之前的研究一致[34]，但导致这一结果的原因尚不明确，目前仅有报道称两者的发病机制可能不同[38]，并且由于胸腺瘤具有复杂的免疫机制及凋亡机制，可能导致了T-MG与NT-MG之间血清UA、BIL、ALB水平无明显差异这一结局。

为了更好的诊断、治疗及评估预后，MG被分为多种亚组。其中，根据肌无力累及的肌群，MG被分为了眼肌型MG（OMG）及全身型MG（GMG），除此之外，在部分学者将肌无力症状持续至少24个月且仅表现出眼部症状的病例称为单

中国知网 https://www.cnki.net

纯OMG[40]。OMG患者会因眼外肌或眼轮匝肌受累而出现上睑下垂和复视症状，GMG患者则累及延髓肌、四肢肌、呼吸肌等导致全身多个肌群的无力，故GMG患者常比OMG患者表现出更严重的症状。由于眼外肌的突触结构缺乏二次折叠，且其放电频率较骨骼肌更高，因此大多数MG患者会出现眼外肌症状[41]。据报道，大约85%的MG患者首发症状为上睑下垂和/或复视，高达90%的OMG患者会在发病后两年内发展为GMG[42]。此外，OMG患者的血清AChR-Ab阳性率显著低于GMG患者，而MuSK-Ab抗体阳性的OMG患者将更快的发展为GMG[43]。国内外已有较多研究表明发病年龄、胸腺瘤、血清AChR-Ab阳性、免疫抑制剂治疗、面神经重复电刺激阳性是OMG泛化为GMG的危险因素[44,45]，而女性性别是否作为OMG泛化的预测因素在不同的研究中表现出了不同的结果[40,45]，这些差异除归因于研究人群及样本量的不同外，还因考虑到不同研究对于OMG的定义不同所造成的差异。在本研究中，我们将MG患者按传统分型分为了OMG及GMG组，显而易见的是GMG组患者男女比例低于OMG组，随后我们分别比较了男性及女性不同分型的血清UA、BIL及ALB水平，发现不论性别，OMG患者血清UA、BIL、ALB水平均较GMG患者高，在女性OMG组及GMG组中这种差异更加明显，造成这一结果的可能原因是GMG患者因为累及更多肌群，体内存在更活跃的氧化应激状态，并且女性MG患者抗氧化应激水平较男性低。因此我们得出结论，女性比男性更易患GMG，女性患MG的严重程度普遍较男性高，OMG患者的血清抗氧化水平高于GMG。结合以上结果，我们怀疑血清UA、BIL、ALB水平下降及女性性别可能是导致OMG转化为GMG的危险因素之一，但由于本研究没有纳入患者的初发症状及何时泛化等指标，因此这一猜想尚需进一步实验来证实。

为了进一步验证血清UA、BIL、ALB水平与MG严重程度之间的关系，本研究回顾了随访组患者经治疗前后的QMG评分及血清UA、BIL、ALB水平，所有纳入患者都至少达到了MGFA-PIS分型量表中的改善（I）评级，即与治疗前相比，肌无力临床症状明显减轻或 MG 治疗药物剂量明显减少。我们的研究发现，经治疗后患者的血清UA、TBIL、DBIL、IBIL、ALB水平均有所升高，提示了抗氧化水平的升高。在随后的研究中，我们分别比较了治疗前后QMG评分与血清UA、BIL、ALB水平的关系，结果发现急性期患者的QMG评分与血清UA、TBIL、IBIL、ALB水平均呈负相关，而在症状缓解后，QMG评分仅与UA及ALB呈负相关关系，我们推测造成这一现象的原因是因为UA的代谢效率较快以及部分患者接受了白

中国知网 https://www.cnki.net

蛋白治疗有关，因此在MG急性期，血清UA、TBIL、IBIL、ALB水平一定程度上可反映患者的严重程度。Yang等人[18,35]的研究已经发现MG患者UA、ALB水平与MGFA分型相关，而MGFA分型按照受累肌群及严重程度划分，因此他们认为MG患者血清UA、ALB水平的相对降低与疾病进展程度相关。本研究采用了QMG评分评估患者的严重程度，其客观性优于MGFA分型，研究结果表明了BIL与UA、ALB一样可反映患者的疾病严重程度，适度高水平的UA、BIL、ALB能减轻MG患者病情，因此血清UA、BIL及ALB或许可以作为MG患者急性期严重程度及治疗效果的一个客观评估指标。

氧化应激在免疫相关疾病中的作用机制较为复杂，目前关于MG与氧化应激的机制研究甚少。已有研究发现ROS可作为第二信使参与T、B细胞的信号传导，影响其分化、增殖及凋亡[46,47]。ROS除了影响免疫细胞外，还可损伤AchR。Venkatesham等人[48]的研究发现高浓度的ROS可对AchR造成持续损害，抗氧化剂在AchR氧化损伤中起到主要保护作用。多项研究表明ROS可诱导神经元nAChRs进入失活状态[49,50]，Krishnaswamy等人[51] 表明ROS可通过神经元AchR上高度保守的半胱氨酸残基来介导受体的失活。因此，MG患者体内氧化应激的发生，除了通过影响T、B淋巴细胞等免疫细胞的分化发育来影响MG发生发展外，还可造成AchR受损，进一步加重MG的严重程度。综上所述，血清UA、BIL、ALB作为人体的内源性抗氧化剂，既往多被当成人体代谢所产生的废物，随着对其深入的研究，人们逐渐发现了其在抗氧化应激方面的作用。人体在氧化应激过程中常伴随着大量ROS的产生，UA、BIL、ALB均通过可清除ROS起到抗氧化应激作用，故推测MG患者因体内升高的ROS，启动氧化应激级联反应从而使UA、BIL、ALB等抗氧化物质水平下降，随着病情好转，氧化应激水平下降，血清UA、BIL、ALB水平则有所恢复，因此，在生理范围内适当提高血清UA、BIL、ALB水平可能成为MG的有效治疗策略。

目前为止，已有数十项研究记录了UA、BIL、ALB等内源性抗氧化剂与心血管疾病、糖尿病、癌症、自身免疫性疾病或精神疾病之间的关联。作为一项容易检测的实验室指标，上述指标有望成为监测MG疾病发展、评估治疗疗效的生物标志物。本研究存在一定的局限性，由于疾病治疗的需要，大多数患者均接受了多种药物的治疗，例如皮质类固醇、免疫抑制剂等，这些药物可能会影响患者的血清UA、BIL、ALB水平，需在以后的研究中进一步分析及补充。

中国知网 https://www.cnki.net

4 结 论

1.MG 女性患者血清 UA、TBIL、DBIL、IBIL、ALB 水平均低于男性患者，可能是女性较男性更易患 MG 的原因之一，女性比男性更易患全身型 MG，眼肌型 MG 患者的血清抗氧化水平高于全身型 MG 患者，血清 UA、BIL、ALB 水平下降及女性性别可能是导致 OMG 转化为 GMG 的危险因素之一。

2.有无胸腺瘤 MG 患者之间的血清 UA、BIL、ALB 水平无明显差异，可能是由于胸腺瘤相关 MG 的发病机制不同以及胸腺瘤复杂的免疫机制及凋亡机制导致的。

3.血清 UA、BIL、ALB 水平或许可以作为评价 MG 患者急性期疾病严重程度的一个客观指标，其有望成为监测 MG 疾病发展、评估治疗疗效的生物标志物。

中国知网 https://www.cnki.net

参考文献

[1] Beloor S A, Asuncion R. Myasthenia Gravis[J], 2022.

[2] Dresser L, Wlodarski R, Rezania K, Soliven B. Myasthenia Gravis: Epidemiology, Pathophysiology and Clinical Manifestations[J]. Journal of Clinical Medicine, 2021,10(11).

[3] 常婷. 中国重症肌无力诊断和治疗指南(2020版)[J]. 中国神经免疫学和神经病学杂志, 2021,28(01):1-12.

[4] Gilhus N E, Verschuuren J J. Myasthenia Gravis: Subgroup Classification and Therapeutic Strategies[J]. Lancet Neurology, 2015,14(10):1023-1036.

[5] Adamczyk B, Adamczyk-Sowa M. New Insights Into the Role of Oxidative Stress Mechanisms in the Pathophysiology and Treatment of Multiple Sclerosis[J]. Oxidative Medicine and Cellular Longevity, 2016,2016:1973834.

[6] Wojcik P, Gegotek A, Zarkovic N, Skrzydlewska E. Oxidative Stress and Lipid Mediators Modulate Immune Cell Functions in Autoimmune Diseases[J]. International Journal of Molecular Sciences, 2021,22(2).

[7] Adamczyk-Sowa M, Bieszczad-Bedrejczuk E, Galiniak S, Rozmilowska I, Czyzewski D, Bartosz G, Sadowska-Bartosz I. Oxidative Modifications of Blood Serum Proteins in Myasthenia Gravis[J]. Journal of Neuroimmunology, 2017,305:145-153.

[8] Li L, Shou Y, Borowitz J L, Isom G E. Reactive Oxygen Species Mediate Pyridostigmine-Induced Neuronal Apoptosis: Involvement of Muscarinic and Nmda Receptors[J]. Toxicol Appl Pharmacol, 2001,177(1):17-25.

[9] Ziberna L, Martelanc M, Franko M, Passamonti S. Bilirubin is an Endogenous Antioxidant in Human Vascular Endothelial Cells[J]. Sci Rep, 2016,6:29240.

[10] Mikami T, Sorimachi M. Uric Acid Contributes Greatly to Hepatic Antioxidant Capacity Besides Protein[J]. Physiological Research, 2017,66(6):1001-1007.

[11] Anraku M, Chuang V T, Maruyama T, Otagiri M. Redox Properties of Serum Albumin[J]. Biochim Biophys Acta, 2013,1830(12):5465-5472.

[12] 李柱一. 中国重症肌无力诊断和治疗指南2015[J]. 中华神经科杂志, 2015,48(11):934-940.

[13] 闫旭东, 张莹. 重症肌无力临床常用测评量表的介绍与应用[J]. 中国临床神经科学,

中国知网 https://www.cnki.net

2019,27(03):339-346.

[14] Jaretzki A R, Barohn R J, Ernstoff R M, Kaminski H J, Keesey J C, Penn A S, Sanders D B. Myasthenia Gravis: Recommendations for Clinical Research Standards. Task Force of the Medical Scientific Advisory Board of the Myasthenia Gravis Foundation of America[J]. Neurology, 2000,55(1):16-23.

[15] Uzawa A, Kuwabara S, Suzuki S, Imai T, Murai H, Ozawa Y, Yasuda M, Nagane Y, Utsugisawa K. Roles of Cytokines and T Cells in the Pathogenesis of Myasthenia Gravis[J]. Clinical and Experimental Immunology, 2021,203(3):366-374.

[16] Ramani S, Pathak A, Dalal V, Paul A, Biswas S. Oxidative Stress in Autoimmune Diseases: An Under Dealt Malice[J]. Curr Protein Pept Sci, 2020,21(6):611-621.

[17] Kaminski H J, Himuro K, Alshaikh J, Gong B, Cheng G, Kusner L L. Differential Rna Expression Profile of Skeletal Muscle Induced by Experimental Autoimmune Myasthenia Gravis in Rats[J]. Frontiers in Physiology, 2016,7:524.

[18] Yang D, Su Z, Wu S, Bi Y, Li X, Li J, Lou K, Zhang H, Zhang X. Low Antioxidant Status of Serum Bilirubin, Uric Acid, Albumin and Creatinine in Patients with Myasthenia Gravis[J]. International Journal of Neuroscience, 2016,126(12):1120-1126.

[19] Pizzino G, Irrera N, Cucinotta M, Pallio G, Mannino F, Arcoraci V, Squadrito F, Altavilla D, Bitto A. Oxidative Stress: Harms and Benefits for Human Health[J]. Oxidative Medicine and Cellular Longevity, 2017,2017:8416763.

[20] Nalsen C, Ohrvall M, Kamal-Eldin A, Vessby B. Plasma Antioxidant Capacity Among Middle-Aged Men: The Contribution of Uric Acid[J]. Scand J Clin Lab Invest, 2006,66(3):239-248.

[21] Glantzounis G K, Tsimoyiannis E C, Kappas A M, Galaris D A. Uric Acid and Oxidative Stress[J]. Curr Pharm Des, 2005,11(32):4145-4151.

[22] Abrams B. How Gout Inhibits the Development of Cell-Mediated Autoimmune Disease[J]. Medical Hypotheses, 2009,72(1):102-103.

[23] Hooper D C, Spitsin S, Kean R B, Champion J M, Dickson G M, Chaudhry I, Koprowski H. Uric Acid, a Natural Scavenger of Peroxynitrite, in Experimental Allergic Encephalomyelitis and Multiple Sclerosis[J]. Proc Natl Acad Sci U S A, 1998,95(2):675-680.

[24] Tell G, Gustincich S. Redox State, Oxidative Stress, and Molecular Mechanisms of

中国知网 https://www.cnki.net

Protective and Toxic Effects of Bilirubin On Cells[J]. Curr Pharm Des, 2009,15(25):2908-2914.

[25] Zelenka J, Muchova L, Zelenkova M, Vanova K, Vreman H J, Wong R J, Vitek L. Intracellular Accumulation of Bilirubin as a Defense Mechanism Against Increased Oxidative Stress[J]. Biochimie, 2012,94(8):1821-1827.

[26] Basiglio C L, Arriaga S M, Pelusa F, Almara A M, Kapitulnik J, Mottino A D. Complement Activation and Disease: Protective Effects of Hyperbilirubinaemia[J]. Clin Sci (Lond), 2009,118(2):99-113.

[27] Rocuts F, Zhang X, Yan J, Yue Y, Thomas M, Bach F H, Czismadia E, Wang H. Bilirubin Promotes De Novo Generation of T Regulatory Cells[J]. Cell Transplantation, 2010,19(4):443-451.

[28] Anraku M, Chuang V T, Maruyama T, Otagiri M. Redox Properties of Serum Albumin[J]. Biochim Biophys Acta, 2013,1830(12):5465-5472.

[29] Roche M, Rondeau P, Singh N R, Tarnus E, Bourdon E. The Antioxidant Properties of Serum Albumin[J]. Febs Letters, 2008,582(13):1783-1787.

[30] Belinskaia D A, Voronina P A, Goncharov N V. Integrative Role of Albumin: Evolutionary, Biochemical and Pathophysiological Aspects[J]. J Evol Biochem Physiol, 2021,57(6):1419-1448.

[31] Peng F, Yang Y, Liu J, Jiang Y, Zhu C, Deng X, Hu X, Chen X, Zhong X. Low Antioxidant Status of Serum Uric Acid, Bilirubin and Albumin in Patients with Neuromyelitis Optica[J]. European Journal of Neurology, 2012,19(2):277-283.

[32] Moccia M, Lanzillo R, Palladino R, Russo C, Carotenuto A, Massarelli M, Vacca G, Vacchiano V, Nardone A, Triassi M, Morra V B. Uric Acid: A Potential Biomarker of Multiple Sclerosis and of its Disability[J]. Clinical Chemistry and Laboratory Medicine, 2015,53(5):753-759.

[33] Fuhua P, Xuhui D, Zhiyang Z, Ying J, Yu Y, Feng T, Jia L, Lijia G, Xueqiang H. Antioxidant Status of Bilirubin and Uric Acid in Patients with Myasthenia Gravis[J]. Neuroimmunomodulation, 2012,19(1):43-49.

[34] 周霞, 孙中武. 重症肌无力患者血清胆红素和尿酸变化及其意义[J]. 中华医学杂志, 2013(17):1287-1291.

中国知网 https://www.cnki.net

[35] Yang D, Weng Y, Lin H, Xie F, Yin F, Lou K, Zhou X, Han Y, Li X, Zhang X. Serum Uric Acid Levels in Patients with Myasthenia Gravis are Inversely Correlated with Disability[J]. Neuroreport, 2016,27(5):301-305.

[36] Huang J H, Li R H, Huang S L, Sia H K, Yu C H, Tang F C. Gender Difference in the Relationships Between Inflammatory Markers, Serum Uric Acid and Framingham Risk Score[J]. Int J Environ Res Public Health, 2021,18(13).

[37] Saegeman V S, Vierendeels I, Moens M J, Moerman J. Should Gender-Related Reference Values be Used for Total Bilirubin?[J]. Clinical Chemistry and Laboratory Medicine, 2009,47(10):1309-1310.

[38] Okumura M, Fujii Y, Shiono H, Inoue M, Minami M, Utsumi T, Kadota Y, Sawa Y. Immunological Function of Thymoma and Pathogenesis of Paraneoplastic Myasthenia Gravis[J]. Gen Thorac Cardiovasc Surg, 2008,56(4):143-150.

[39] Alvarez-Velasco R, Gutierrez-Gutierrez G, Trujillo J C, Martinez E, Segovia S, Arribas-Velasco M, Fernandez G, Paradas C, Velez-Gomez B, Casasnovas C, Nedkova V, Guerrero-Sola A, Ramos-Fransi A, Martinez-Pineiro A, Pardo J, Sevilla T, Gomez-Caravaca M T, Lopez D M A, Jerico I, Pelayo-Negro A L, Martin M A, Morgado Y, Mendoza M D, Perez-Perez H, Rojas-Garcia R, Turon-Sans J, Querol L, Gallardo E, Illa I, Cortes-Vicente E. Clinical Characteristics and Outcomes of Thymoma-Associated Myasthenia Gravis[J]. European Journal of Neurology, 2021,28(6):2083-2091.

[40] Mazzoli M, Ariatti A, Valzania F, Kaleci S, Tondelli M, Nichelli P F, Galassi G. Factors Affecting Outcome in Ocular Myasthenia Gravis[J]. International Journal of Neuroscience, 2018,128(1):15-24.

[41] O'Hare M, Doughty C. Update On Ocular Myasthenia Gravis[J]. Seminars in Neurology, 2019,39(6):749-760.

[42] Nair A G, Patil-Chhablani P, Venkatramani D V, Gandhi R A. Ocular Myasthenia Gravis: A Review[J]. Indian Journal of Ophthalmology, 2014,62(10):985-991.

[43] Melson A T, McClelland C M, Lee M S. Ocular Myasthenia Gravis: Updates On an Elusive Target[J]. Current Opinion in Neurology, 2020,33(1):55-61.

[44] Witthayaweerasak J, Rattanalert N, Aui-Aree N. Prognostic Factors for Conversion to Generalization in Ocular Myasthenia Gravis[J]. Medicine (Baltimore), 2021,100(19):e25899.

中国知网 https://www.cnki.net

[45] Feng X, Huan X, Yan C, Song J, Lu J, Zhou L, Wu H, Qiao K, Lu J, Xi J, Luo S, Zhao C. Adult Ocular Myasthenia Gravis Conversion: A Single-Center Retrospective Analysis in China[J]. European Neurology, 2020,83(2):182-188.

[46] Belikov A V, Schraven B, Simeoni L. T Cells and Reactive Oxygen Species[J]. Journal of Biomedical Science, 2015,22:85.

[47] Tohyama Y, Takano T, Yamamura H. B Cell Responses to Oxidative Stress[J]. Curr Pharm Des, 2004,10(8):835-839.

[48] A V, DR K, J V S, P S B. Effect of Reactive Oxygen Species On Cholinergic Receptor Function[J]. Indian Journal of Pharmacology, 2005,37(6).

[49] Campanucci V A, Krishnaswamy A, Cooper E. Mitochondrial Reactive Oxygen Species Inactivate Neuronal Nicotinic Acetylcholine Receptors and Induce Long-Term Depression of Fast Nicotinic Synaptic Transmission[J]. Journal of Neuroscience, 2008,28(7):1733-1744.

[50] Zhao J, Zheng Y, Xue F, Chang Y, Yang H, Zhang J. Molecular Basis of Reactive Oxygen Species-Induced Inactivation of Alpha4Beta2 Nicotinic Acetylcholine Receptors[J]. Free Radic Biol Med, 2016,97:520-530.

[51] Krishnaswamy A, Cooper E. Reactive Oxygen Species Inactivate Neuronal Nicotinic Acetylcholine Receptors through a Highly Conserved Cysteine Near the Intracellular Mouth of the Channel: Implications for Diseases that Involve Oxidative Stress[J]. J Physiol, 2012,590(1):39-47.

中国知网 https://www.cnki.net

综述

氧化应激在重症肌无力中的研究进展

刘磊 综述　　张艺凡 审校

概要：

重症肌无力（MG）是一种自身抗体介导的自身免疫性疾病，其可累及全身多个肌群造成严重的肌无力症状。T、B、NK等免疫细胞参与了MG的发生发展，AchR抗体等自身抗体的产生是其发病机制。近年来，氧化应激（OS）已被证明参与了SLE、MS、RA等自身免疫性疾病的发病过程，已有研究也表明MG患者体内存在活跃的OS过程。本文就OS对免疫系统的影响及其在MG患者中的研究作一综述，以期为MG的发病机制及治疗提供新的思路。

关键词：

重症肌无力；氧化应激；活性氧；免疫细胞；自身免疫

重症肌无力 (Myasthenia Gravis,MG) 是一种自身抗体介导的自身免疫性疾病，也是最常见的神经肌肉接头疾病，重症肌无力的诊断基于临床和血清学检查，患有此病的患者的主要特征是受累肌肉的晨轻暮重表现。MG 可累及包括呼吸机在内的全身多个肌群，但由于眼外肌的突触结构与骨骼肌不同，并且有更高频率的突触放电，因此眼外肌更常受到影响。据统计，超过 50%的 MG 患者的最初表现为上睑下垂和复视，这些患者大多数在发病最初两年内发展为全身性疾病[1]，因此，MG 是一种严重影响患者生命及生活质量的疾病。T 细胞、B 细胞、NK 细胞等免疫细胞已被证明参与了 MG 的发生发展[2,3]，免疫细胞调节失衡导致 MG 患者产生致病性自身抗体是其主要发病机制，免疫调节治疗也是现在治疗 MG 的主要方法。引起 MG 的主要自身抗体是抗乙酰胆碱受体(Acetylcholine Receptors，AChR)抗体[4]，除此以外，抗肌肉特异性受体酪氨酸激酶(MuSK)抗体、抗低密度脂蛋白受体相关蛋白 4 (LRP4)等抗体也可在部分 MG 患者血清中检测到，AChR 抗体通过加速受体降解、诱导 AChR 构象变化或阻断乙酰胆碱（ACh）结合而直接致病[1]。近年来，已有较多研究表明氧化应激参与了 MG 的发病机制，本文就

中国知网 https://www.cnki.net

此方面的研究进展进行综述如下。

1.活性氧（Reactive Oxygen Species,ROS）及氧化应激（Oxidative Stress,OS）

ROS 是是一类含氧活性物质的总称，包括超氧阴离子($O2^{\bullet-}$)、过氧化氢(H_2O_2)、羟基自由基($OH^{\bullet}$)等，其主要来源是线粒体呼吸链和还原型辅酶II（NADPH）[5]。ROS 的动态平衡在减少氧化损伤和满足能量需求方面起着重要作用，低浓度的 ROS 对于细胞存活、细胞生长、增殖和分化以及免疫反应等多种生物学功能是必要的，ROS 的过量产生被认为是导致许多疾病发病的主要因素，包括癌症、炎症性疾病、神经系统疾病、心血管疾病以及生物体的衰老过程[6]。氧化应激这一概念最初是在 1985 年被提出，人体的有氧代谢与 ROS 的产生有关，在生物进化过程中，也形成了一定的抗氧化能力，包括抗氧化酶和非酶抗氧化剂，可减少 ROS 的不利影响，人体在受到内源性或外源性损伤后，由于代谢、环境、食物等多种因素的影响，产生了大量 ROS，导致促氧化剂和抗氧化剂之间的不平衡，造成机体损伤，被称为氧化应激[7]。

2.OS 与免疫调节的关系

自身免疫被定义为针对宿主体内抗原的免疫反应，自身免疫性疾病则是由对自身抗原的免疫耐受性丧失引发的疾病。近年来的研究已经证实了 ROS 通过影响各类淋巴细胞活性参与了免疫反应的病理生理过程[8]。

2.1 ROS 与自然杀伤（Natural Killer,NK）细胞

NK 细胞在机体正常的免疫、防御机制中起重要作用，是先天性免疫系统的主要效应细胞。早年间的研究证实了 ROS 通过降低 NK 细胞中 CD3 和 CD16 的表达，导致信号转导改变，进而抑制 NK 细胞的功能[9]；Keap1-Nrf2 通路是抗氧化防御的重要信号通路，一项对系统性红斑狼疮（SLE）患者的研究发现 NK 细胞中的 Keap1 及 Nrf2 表达下降，表明 SLE 患者中 NK 细胞抗氧化防御系统受损[10]。此外，单核细胞衍生的 ROS 可诱导 NK 细胞的增殖、凋亡及细胞毒性[11]，根据这些发现，提示了 ROS 在调节 NK 细胞的分化、活化及凋亡等均起到调节作用。

2.2 ROS 与树突状细胞（Dendritic Cell,DC）

DC 是最重要的抗原呈递细胞（Antigen Presenting Cell,APC），在抗原特异性免疫反应及效应 T 细胞的调节中起关键作用。DC 的成熟过程可以被 H_2O_2 或 $O2^{\bullet-}$ 诱导，并且可以被抗氧化剂抵消[11]。例如，一项体外研究表明经黄

中国知网 https://www.cnki.net

嘌呤氧化酶（一种能产生ROS的酶）处理的DC呈现出更强的成熟性[12]，表明氧化应激可提高DC的抗原提呈活性。此外，氧化应激可增加DC促炎细胞因子的产生、降低抑炎细胞因子的产生，还可增强DC的迁移能力[13]。

2.3 ROS与T细胞

ROS可以作为细胞信号传导中的次要信使，通过氧化还原激活信号系统调节T细胞功能[14]。目前对于T细胞的研究大多集中在$CD4^+$T细胞上，$CD4^+$T细胞需分化为Treg、Th1、Th2、Th17等亚型以发挥免疫活性，在这一过程中需要大量ATP的支持，因此，正常的$CD4^+$T细胞会上调糖酵解并产生 ATP、ROS 和三羧酸循环产物。在类风湿性关节炎（Rheumatoid Arthritis,RA）患者的研究中观察到，T 细胞下调糖酵解，并将葡萄糖分流到磷酸戊糖途径(Pentose Phosphate Pathway,PPP)，以减少ROS的产生，促进NADPH的产生，维持谷胱甘肽的还原状态[14]。而在ROS清除剂的存在下培养健康的$CD4^+$T细胞可以促进Th1和Th17等促炎细胞亚群分化定型，且主要发挥抗炎效应的Th2及Treg似乎不受影响，提示了过低的ROS会导致T细胞的促炎效应增强[16]。此外，有研究表明激活的中性粒细胞抑制了人类T细胞的DNA合成，并且与培养基中的超氧化物水平成比例[17]，另一项研究也表明人类$CD4^+$T细胞与粒细胞共培养后活性降低，而添加过氧化氢酶则逆转了这一现象，表明粒细胞产生的ROS可以到达T细胞并对T 细胞的信号传递、激活、增殖产生负面影响[18]。正如前所述，ROS 可作为细胞信号传导的第二信使，这一作用在H_2O_2中尤为明显，这是因为它足够稳定，能够穿过细胞膜，并且优先与半胱氨酸残基反应[19]，因此，在一项研究Treg对氧化应激的敏感性研究中将H_2O_2作为促氧化剂来模拟氧化应激，研究结果表明了Treg细胞可免受H_2O_2诱导的凋亡、小剂量的H_2O_2并不影响Treg的抑制能力[20]。综上所述，在生理条件下，ROS和抗氧化系统之间的平衡是T细胞正常运作所必需的，不论是内源性或外源性途径打破这一平衡，均可对T细胞的正常功能产生影响。

2.4 ROS与B细胞

B细胞在免疫系统的主要作用是产生抗体，H_2O_2在B细胞激活和B细胞受体(B Cell Receptor,BCR)信号转导中起重要作用。在B细胞中，H_2O_2刺激后最早的事件之一是细胞蛋白的酪氨酸磷酸化，在B细胞暴露于H_2O_2或刺激 B 细胞中产生H_2O_2的酶后，蛋白酪氨酸磷酸酶被抑制并失去其对BCR的调节能力，导

中国知网 https://www.cnki.net

致脾酪氨酸激酶（Sky）快速活化并增加酪氨酸磷酸化，被激活的 Syk 可以进一步刺激 NADPH 氧化酶产生更多的 ROS[11, 21]，此外，Syk 还可保护细胞免受氧化应激诱导的细胞凋亡[22]。Roberta 等人[23] 跟踪了 B 细胞分化过程中的氧化及抗氧化过程，发现 ROS 伴随着分化过程而产生，并且可被谷胱甘肽等抗氧化系统所抵消。总之，ROS 及其相应的抗氧化系统对 B 细胞的分化及生存起着重要作用，但仍需大量研究进一步阐明其机制。

3.MG 患者体内的低抗氧化状态

许多疾病的过程中均可观察到 OS 的发生，并在其中一些疾病的病因中发挥重要作用，如自身免疫性疾病、神经退行性疾病、炎症及癌症等。作为人体的内源性抗氧化系统的一部分，血清尿酸、胆红素、白蛋白等天然抗氧化剂已被证实在许多自身免疫性疾病中呈下降状态[24,25]。作为一种典型的自身免疫性疾病，同样已有相关报道证明了 MG 患者的血清胆红素、尿酸、白蛋白等水平显著低于健康对照组。Peng 等人[26] 的研究中发现 MG 患者的血清尿酸、总胆红素、直接胆红素、间接胆红素均低于健康人，在多发性硬化（Multiple Sclerosis,MS）患者中也得出相同的结论，此外，他们的研究还发现 MG 患者疾病严重程度与血清尿酸水平或胆红素水平没有显著相关性。在此项研究之后，Yang 等人[27,28] 同样比较了上述指标及肌酐在 MG 患者与健康对照组之间的关系，不同的是，在他们的研究中发现男性 MG 患者的血清直接胆红素水平与男性健康对照组相比并没有显著差异，而在他们将 MG 患者按美国 MG 协会（MGFA）分型将患者分组比较时，发现 MG 患者的平均血清尿酸、白蛋白和肌酐水平的下降程度与疾病严重程度相关，而上述指标在 MG 患者有无胸腺瘤之间的差异无统计学意义。除了这些研究，Adamczyk-Sowa 等人[29] 的研究还通过测定 MG 患者血清蛋白的氧化标记物，证实了 MG 患者发生了 OS，并随着病程发展逐渐加重。对实验性自身免疫性重症肌无力（Experimental Autoimmune Myastheia Gravis,EAMG）大鼠骨骼肌差异 RNA 表达谱的对比分析提示，EAMG 大鼠体内明显扩增的基因属于新陈代谢、应激反应和信号传导的类别，提示 EAMG 可以直接或间接地增加细胞内 ROS 水平。这些研究均表明，MG 患者体内的抗氧化防御能力下降。

4.氧化应激对 MG 患者的影响

MG 是由神经-肌肉接头突触后膜的致病性自身抗体所引起疾病，众所周知，当神经冲动传导至突触前膜导致 Ach 释放，并与突触后膜的 AchR 结合而产生终

中国知网 https://www.cnki.net

板电位，终板电位通过横管系统扩散至整个肌纤维导致肌肉收缩，这一过程受到乙酰胆碱酯酶（AchE）及突触后膜 AchR 数量的影响。已有研究报道在 99% 的全身性 MG 患者和 40% 至 77% 的眼肌型 MG 患者中可检测到抗 AChR 抗体[1]，这一抗体通过结合、阻断或调节 AChR 活性来损害受体功能，导致肌无力的产生。

氧化应激已被证实参与了 SLE、NMO、RA 等自身免疫性疾病的发病机制[24,30,31]，而在 MG 患者中，也有研究提出了抗氧化能力的减少可能是 MG 的早期致病机制[27]，但这一理论尚无更深层次的研究来进一步证实。在 ROS 与 AchR 之间的关系中，已有研究报道了氧自由基可对 AchR 造成抑制或损害。早在 2005 年，Venkatesham 等人[32] 就通过在腹直肌及青蛙心脏制剂中添加不同剂量的 H_2O_2 及 $OH^{\bullet}$ 的方式评估了 ROS 对烟碱型 AchR（nAchR）及毒蕈碱型 AchR（mAchR）的影响，结果发现氧自由基介导的 AchR 损伤与 ROS 的剂量和影响时间相关，并且抗氧化剂可对 nAchR 产生明显的保护作用。在关于神经元 nAchR 的报道中，也提出了在富含线粒体的神经末梢和突触后结构中，线粒体会产生足够的 ROS 来灭活 nAChR[33]，Zhao 等人[34] 的研究进一步证明了 H_2O_2 可以氧化 α4β2-nAChR 连接子中的半胱氨酸，使 nAChR 处于失活状态。综上所述，MG 患者体内存在活跃的 OS 过程，并且 MG 患者体内的抗氧化能力随着疾病的加重而下降，因此，OS 的发生可能会通过影响 MG 患者的免疫细胞活性及损害 MG 患者的 AchR，导致肌无力的发生。

5.总结与展望

在 MG 的发病过程中，淋巴细胞亚群的失衡起到了关键作用，ROS 可通过影响免疫细胞生成、分化及凋亡对疾病的发展起到调控作用，OS 的发生也可通过影响 AchR 的活性影响疾病的发生发展，但目前对于 OS 在 MG 患者中的作用研究尚停留在表象上，仍需更严谨的实验设计来证明其分子机制。补充抗氧化剂已成为许多疾病的新兴治疗方法，在 MG 这一类严重影响人类生存质量的疾病中，积极探索 OS 的发生机制有助于更好的治疗及预防这一疾病。

参考文献

[1] Nair A G, Patil-Chhablani P, Venkatramani D V, Gandhi R A. Ocular Myasthenia Gravis: A Review[J]. Indian Journal of Ophthalmology, 2014,62(10):985-991.

中国知网 https://www.cnki.net

[2] Uzawa A, Kuwabara S, Suzuki S, Imai T, Murai H, Ozawa Y, Yasuda M, Nagane Y, Utsugisawa K. Roles of Cytokines and T Cells in the Pathogenesis of Myasthenia Gravis[J]. Clinical and Experimental Immunology, 2021,203(3):366-374.

[3] Liu R T, Li W, Guo D, Yang C L, Ding J, Xu J X, Duan R S. Natural Killer Cells Promote the Differentiation of Follicular Helper T Cells Instead of Inducing Apoptosis in Myasthenia Gravis[J]. International Immunopharmacology, 2021,98:107880.

[4] Martinez T S, Gomez M I, Martinez G R. [an Update On Myasthenia Gravis][J]. Semergen, 2018,44(5):351-354.

[5] Sarniak A, Lipinska J, Tytman K, Lipinska S. Endogenous Mechanisms of Reactive Oxygen Species (Ros) Generation[J]. Postepy Hig Med Dosw (Online), 2016,70(0):1150-1165.

[6] Yang S, Lian G. Ros and Diseases: Role in Metabolism and Energy Supply[J]. Molecular and Cellular Biochemistry, 2020,467(1-2):1-12.

[7] Sies H. Oxidative Stress: A Concept in Redox Biology and Medicine[J]. Redox Biology, 2015,4:180-183.

[8] Li R, Jia Z, Trush M A. Defining Ros in Biology and Medicine[J]. React Oxyg Species (Apex), 2016,1(1):9-21.

[9] Corsi M M, Maes H H, Wasserman K, Fulgenzi A, Gaja G, Ferrero M E. Protection by L-2-Oxothiazolidine-4-Carboxylic Acid of Hydrogen Peroxide-Induced Cd3Zeta and Cd16Zeta Chain Down-Regulation in Human Peripheral Blood Lymphocytes and Lymphokine-Activated Killer Cells[J]. Biochemical Pharmacology, 1998,56(5):657-662.

[10] Tandon A, Anupam K, Kaushal J, Gautam P, Sharma A, Bhatnagar A. Altered Oxidative Stress Markers in Relation to T Cells, Nk Cells & Killer Immunoglobulin Receptors that are Associated with Disease Activity in Sle Patients[J]. Lupus, 2020,29(14):1831-1844.

[11] Yang Y, Bazhin A V, Werner J, Karakhanova S. Reactive Oxygen Species in the Immune System[J]. International Reviews of Immunology, 2013,32(3):249-270.

[12] Gotz A, Ty M C, Rodriguez A. Oxidative Stress Enhances Dendritic Cell Responses to Plasmodium Falciparum[J]. Immunohorizons, 2019,3(11):511-518.

[13] Batal I, Azzi J, Mounayar M, Abdoli R, Moore R, Lee J Y, Rosetti F, Wang C, Fiorina P, Sackstein R, Ichimura T, Abdi R. The Mechanisms of Up-Regulation of Dendritic Cell Activity by Oxidative Stress[J]. J Leukoc Biol, 2014,96(2):283-293.

中国知网 https://www.cnki.net

[14] Reth M. Hydrogen Peroxide as Second Messenger in Lymphocyte Activation[J]. Nature Immunology, 2002,3(12):1129-1134.

[15] Weyand C M, Shen Y, Goronzy J J. Redox-Sensitive Signaling in Inflammatory T Cells and in Autoimmune Disease[J]. Free Radic Biol Med, 2018,125:36-43.

[16] Yang Z, Shen Y, Oishi H, Matteson E L, Tian L, Goronzy J J, Weyand C M. Restoring Oxidant Signaling Suppresses Proarthritogenic T Cell Effector Functions in Rheumatoid Arthritis[J]. Science Translational Medicine, 2016,8(331):331r-338r.

[17] Cemerski S, Cantagrel A, Van Meerwijk J P, Romagnoli P. Reactive Oxygen Species Differentially Affect T Cell Receptor-Signaling Pathways[J]. Journal of Biological Chemistry, 2002,277(22):19585-19593.

[18] Belikov A V, Schraven B, Simeoni L. T Cells and Reactive Oxygen Species[J]. Journal of Biomedical Science, 2015,22:85.

[19] Winterbourn C C. The Biological Chemistry of Hydrogen Peroxide[J]. Methods Enzymol, 2013,528:3-25.

[20] Mougiakakos D, Johansson C C, Kiessling R. Naturally Occurring Regulatory T Cells Show Reduced Sensitivity Toward Oxidative Stress-Induced Cell Death[J]. Blood, 2009,113(15):3542-3545.

[21] Villamor N, Montserrat E, Colomer D. Cytotoxic Effects of B Lymphocytes Mediated by Reactive Oxygen Species[J]. Curr Pharm Des, 2004,10(8):841-853.

[22] Tohyama Y, Takano T, Yamamura H. B Cell Responses to Oxidative Stress[J]. Curr Pharm Des, 2004,10(8):835-839.

[23] Vene R, Delfino L, Castellani P, Balza E, Bertolotti M, Sitia R, Rubartelli A. Redox Remodeling Allows and Controls B-Cell Activation and Differentiation[J]. Antioxid Redox Signal, 2010,13(8):1145-1155.

[24] Shah D, Mahajan N, Sah S, Nath S K, Paudyal B. Oxidative Stress and its Biomarkers in Systemic Lupus Erythematosus[J]. Journal of Biomedical Science, 2014,21:23.

[25] Peng F, Yang Y, Liu J, Jiang Y, Zhu C, Deng X, Hu X, Chen X, Zhong X. Low Antioxidant Status of Serum Uric Acid, Bilirubin and Albumin in Patients with Neuromyelitis Optica[J]. European Journal of Neurology, 2012,19(2):277-283.

[26] Fuhua P, Xuhui D, Zhiyang Z, Ying J, Yu Y, Feng T, Jia L, Lijia G, Xueqiang H.

中国知网 https://www.cnki.net

Antioxidant Status of Bilirubin and Uric Acid in Patients with Myasthenia Gravis[J]. Neuroimmunomodulation, 2012,19(1):43-49.

[27] Yang D, Su Z, Wu S, Bi Y, Li X, Li J, Lou K, Zhang H, Zhang X. Low Antioxidant Status of Serum Bilirubin, Uric Acid, Albumin and Creatinine in Patients with Myasthenia Gravis[J]. International Journal of Neuroscience, 2016,126(12):1120-1126.

[28] Yang D, Weng Y, Lin H, Xie F, Yin F, Lou K, Zhou X, Han Y, Li X, Zhang X. Serum Uric Acid Levels in Patients with Myasthenia Gravis are Inversely Correlated with Disability[J]. Neuroreport, 2016,27(5):301-305.

[29] Adamczyk-Sowa M, Bieszczad-Bedrejczuk E, Galiniak S, Rozmilowska I, Czyzewski D, Bartosz G, Sadowska-Bartosz I. Oxidative Modifications of Blood Serum Proteins in Myasthenia Gravis[J]. Journal of Neuroimmunology, 2017,305:145-153.

[30] Peng F, Yang Y, Liu J, Jiang Y, Zhu C, Deng X, Hu X, Chen X, Zhong X. Low Antioxidant Status of Serum Uric Acid, Bilirubin and Albumin in Patients with Neuromyelitis Optica[J]. European Journal of Neurology, 2012,19(2):277-283.

[31] Quinonez-Flores C M, Gonzalez-Chavez S A, Del R N D, Pacheco-Tena C. Oxidative Stress Relevance in the Pathogenesis of the Rheumatoid Arthritis: A Systematic Review[J]. Biomed Research International, 2016,2016:6097417.

[32] A V, DR K, J V S, P S B. Effect of Reactive Oxygen Species On Cholinergic Receptor Function[J]. Indian Journal of Pharmacology, 2005,37(6).

[33] Krishnaswamy A, Cooper E. Reactive Oxygen Species Inactivate Neuronal Nicotinic Acetylcholine Receptors through a Highly Conserved Cysteine Near the Intracellular Mouth of the Channel: Implications for Diseases that Involve Oxidative Stress[J]. J Physiol, 2012,590(1):39-47.

[34] Zhao J, Zheng Y, Xue F, Chang Y, Yang H, Zhang J. Molecular Basis of Reactive Oxygen Species-Induced Inactivation of Alpha4Beta2 Nicotinic Acetylcholine Receptors[J]. Free Radic Biol Med, 2016,97:520-530.

中国知网 https://www.cnki.net

中国知网 https://www.cnki.net

致 谢

行文至此，落笔为终。三年的研究生生涯至此结束，回忆实习时首次踏进贵医，各位老师的指导均带给了我深刻的影响，三年来的学习生涯，使我倍感留恋。三年前，我站在此处，怀着对研究生学习的憧憬，三年后，我仍在此处，向人生交上不完美但无遗憾的答卷，在这一晃而过的三年青春里，见证了我的成长与改变，酸甜苦辣皆于心头，但更多的是感激。

桃李不言，下自成蹊，在此，衷心感谢我的导师张艺凡老师，给了我继续深造的机会，也在我的论文攥写过程中给了我很大的帮助，张艺凡老师严谨治学、亲切随和的治学态度，以及兢兢业业、认真负责的工作态度，带给了我巨大的影响，是我终生学习的榜样，师恩难忘，铭记于心。其次，感谢在我学习生活中给过我一切帮助的师兄师姐、师弟师妹们，希望你们在今后前程似锦、一帆风顺。最后，感谢所有教研室的老师们，是你们的的教学与指导，让我丰富了理论知识，提高了自己在学习过程中的认知能力和克服困难的勇气。与此同时，感谢参加本论文评阅、答辩、和提出宝贵意见的各位老师。

山水有别，愿我们均能登高望顶！

中国知网 https://www.cnki.net

学位论文数据集

关键词*	密级*	中图分类号*	UDC	论文资助
重症肌无力;尿酸;胆红素;白蛋白；氧化应激	☑公开<br>☐不公开	R746.1	616.8	

学位授予单位名称*	学位授予单位代码*	学位类别*	学位级别*
贵州医科大学	10660	专业学位	医学硕士

论文题名*	论文语种*
重症肌无力患者血清尿酸、胆红素、白蛋白水平变化的临床分析	中文

并列题名*	Clinical Analysis of Changes in Serum Uric Acid, Bilirubin and Albumin Levels in Patients with Myasthenia Gravis		
作者姓名*	刘磊	学号*	2019120020232

培养单位名称*	培养单位代码*	培养单位地址	邮编
贵州医科大学	10660	贵州省贵阳市云岩区北京路 9 号	550004

学科专业*	研究方向*	学制*	学位授予年*
神经病学	重症肌无力	三年	2022 年

论文提交日期*	2022 年 6 月		
导师姓名*	张艺凡	职称*	主任医师

评阅人	答辩委员会主席*	答辩委员会成员
	商慧芳	徐平、蔡毅媛、吴珊、于云莉

电子版论文提交格式 文本（✔） 图像（） 视频（） 音频（） 多媒体（） 其它（）<br>推荐格式：application/msword；application/pdf

电子版论文出版（发布）者	电子版论文出版（发布）地	权限声明

论文总页数*	44
共 33 项，其中带*为必填数据，为 22 项。	

中国知网 https://www.cnki.net

RESEARCH PAPER *Multiple Sclerosis* 2008; **14**: 188–196

Serum uric acid levels of patients with multiple sclerosis and other neurological diseases

Fuhua Peng[1], Bin Zhang[1], Xiufeng Zhong[2], Jin Li[1], Guihong Xu[1], Xueqiang Hu[1], Wei Qiu[1] and Zhong Pei[3]

The serum uric acid (UA) levels were measured in 112 patients with multiple sclerosis (MS) and 794 patients with different types of other neurological diseases (OND) or healthy control group. Serum UA levels, along with relevant clinical parameters of MS and OND, were also investigated. MS patients had significantly lower UA levels than those with transient ischemia attack (344.6 ± 130.6 μmol/L, $P = 0.000$), cerebral hemorrhage (311.9 ± 104.7 μmol/L, $P = 0.000$), cerebral infarction (291.3 ± 101.6 μmol/L, $P = 0.014$) and the healthy control group (312.1 ± 92.8 μmol/L, $P = 0.000$). MS patients had significantly higher serum UA levels than those with cryptococcus meningitis or meningoencephalitis (178.9 ± 107.0 μmol/L, $P = 0.000$) and tuberculous meningitis or meningoencephalitis patients (175.7 ± 99.9 μmol/L, $P = 0.000$). There were no significant differences in UA levels between patients with MS and those with facial neuritis, viral meningitis or encephalitis, pulmonary tuberculosis, polymyositis or dermatomyositis, myasthenia gravis, subarachnoid hemorrhage, migraine, Guillain–Barre syndrome and myelitis. In addition, UA levels were independently correlated with gender and duration of MS, but neither with MRI activity, disability nor subtypes of the disease in MS patients. Our data suggest that UA has two biphasic functions: neuroprotective and injurious. Our studies may help physicians to deal with conditions having abnormal UA levels. *Multiple Sclerosis* 2008; **14**: 188–196. http://msj.sagepub.com

Key words: multiple sclerosis; neuroprotection; other neurological diseases; treatment; uric acid

Introduction

Multiple sclerosis (MS) is a chronic inflammatory demyelinating disease of the central nervous system (CNS). Nitric oxide (NO) and its oxidizing congeners, such as peroxynitrite (PN), have been implicated in the immunopathogenesis of MS and its animal model, experimental autoimmune encephalomyelitis (EAE) [1]. By virtue of its potent oxidant activity, PN is believed to be responsible for the majority of damage to CNS attributed to NO [2].

Uric acid (UA), the end product of purine metabolism, is a known scavenger of PN [3]. There is evidence to show that treatment with UA promotes the recovery of neurological function in mice with EAE [3,4]. In addition, it is well established that patients suffering from MS have lower serum UA levels than the healthy control subjects or patients with other neurological diseases (OND) [3–5]. The literature reports inconsistent results on the relationship between UA level and disease activity. In some studies, UA levels were significantly correlated with disease activity and immunomodulating or immunosuppressing drugs [5,6]. The authors of these studies suggested that UA might serve as an easily detectable marker of disease activity as well as response to therapy [7]. On the other hand, other studies did not suggest the correlation [8,9].

[1] Department of Neurology, The Third Affiliated Hospital of Sun Yat-Sen University, 600 Tianhe Road, Guangzhou 510630, Guangdong Province, People's Republic of China
[2] State Key Laboratory of Ophthalmology, Zhongshan Ophthalmic Center of Sun Yat-Sen University, 54 Xianlie Road, Guangzhou 510060, Guangdong Province, People's Republic of China
[3] Department of Neurology, The First Affiliated Hospital of Sun Yat-Sen University, 89 Zhongshaner Road, Guangzhou 510080, Guangdong Province, People's Republic of China
Author for correspondence: Xueqiang Hu, MD, Department of Neurology, The Third Affiliated Hospital of Sun Yat-Sen University, 600 Tianhe Road, Guangzhou 510630, Guangdong Province, People's Republic of China. E-mail: huxueqiangzssy@yahoo.com.cn
Received 12 February 2007; revised 28 April 2007; accepted 30 May 2007

© 2008 SAGE Publications
Los Angeles, London, New Delhi and Singapore
10.1177/1352458507082143

Furthermore, the relationship between UA levels of patients with MS and those of specific types of OND were not investigated individually. Moreover, the effect of gender and age was not evaluated in the majority of previous studies. It is well known that gender and age have an important impact on the level of serum UA [4,5]. Additionally, some types of OND have much more severe inflammation or damage of blood–brain barrier than MS. In the light of such considerations, we therefore conducted a hospital-based study aimed at determining whether serum UA levels of patients with MS were lower than those of different types of OND. In addition, we also investigated the relationship between serum UA levels of MS patients and clinical parameters of MS patients, such as gender, duration of disease, MRI activity, disability and subtypes of diseases.

Patients and methods

Serum samples were collected from 908 individuals comprising 112 patients with MS and 796 patients with OND or healthy subjects divided into 15 groups: myelitis, Guillain–Barre syndrome, myasthenia gravis, polymyositis or dermatomyositis, facial neuritis, viral meningitis or encephalitis, cryptococcus meningitis or meningoencephalitis, tuberculous meningitis or meningoencephalitis, pulmonary tuberculosis, transient ischemia attack (TIA), cerebral infarction, cerebral hemorrhage, subarachnoid hemorrhage, migraine and healthy controls. All the patients had been in hospital and had definite diagnosis according to the different disease diagnostic criteria. Demographic and clinical characteristics of MS patients, patients with OND and healthy control group are presented in Table 1.

All MS patients had definite MS according to the criteria of Poser *et al.* [10] or McDonald *et al.* [11]. Most reasons for hospitalization were for diagnostic or therapeutic purposes in patients with clinically active disease (defined as the development within the previous two weeks of new neurological symptoms or signs attributable to demyelination). All the MS patients were scored by the Expanded Disability Status Scale (EDSS) [12]. Mean EDSS score was 3.5 ± 2.2, range 1–9.5. The mean duration of the disease was 5.2 ± 3.8 years, range 0.1–15 years. Of the 112 patients with MS, 78 had the relapsing–remitting (RR) type of the disease, 6 had primary progressive–remitting MS and 18 the primary progressive (PP) type of the disease.

Patients with MS were divided into two groups: 53 with brain or spinal cord [magnetic resonance imaging (MRI)] activity and 31 without. Patients with MS were considered to have MRI activity if they had one or more enhancing lesions in T1-weighted spin-echo images after gadopentate dimeglumine (Gd-DTPA) injection. Gd-DTPA was given intravenously at a dose of 0.1 mmol/kg, and ~15 min after contrast injection, the T1-weighted sequence was repeated.

Ethical consent has been obtained by all patients and healthy controls for studying UA serum levels. Exclusion criteria were treatment with acetylsalicylic acid, thiazide diuretics, steroids, ibuprophen and other drugs that could increase or affect UA levels, as well as subjects with diabetes or renal failure.

Table 1 Demographic and clinical characteristics of MS patients, patients with OND and healthy control group

Patients	Number of Patients	Male	Female	Ages	Mean of ages	Duration
MS	112	70	42	4–68	33.5	5.2 ± 3.8 y
Myelitis	42	24	18	5–83	37.6	8.2 ± 4.1 d
Guillain–Barre syndrome	41	28	13	5–74	35.1	5.5 ± 1.3 d
Myasthenia gravis	42	22	20	2–69	28.8	0.9 ± 0.4 y
Polymyositis or dermatomyositis	49	20	29	12–73	41.1	2.3 ± 1.5 y
Facial neuritis	60	29	31	3–74	46.1	3.6 ± 1.7 d
Viral meningitis or encephalitis	70	41	29	5–58	27.4	6.8 ± 3.4 d
Cryptococcus meningitis or meningoencephalitis	59	44	15	5–73	36.6	15.2 ± 5.2 d
Tuberculous meningitis or meningoencephalitis	49	26	23	14–70	38.1	17.6 ± 8.9 d
Pulmonary tuberculosis	59	37	22	15–81	44.8	20.3 ± 10.8 d
Transient ischemia attack	38	26	12	43–82	64.1	3.4 ± 1.1 d
Cerebral infarction	84	50	34	29–92	56.0	2.5 ± 1.4 d
Cerebral hemorrhage	44	29	15	21–86	56.6	2.2 ± 1.9 d
Subarachnoid hemorrhage	43	21	22	16–80	49.5	1.9 ± 0.6 d
Migraine	27	5	22	24–79	47.7	1.2 ± 0.8 y
Healthy control group	89	43	46	20–68	41.0	

y, year; d, day.

All patients and control subjects completed a diet questionnaire, including average weekly meat and alcohol consumption. Blood was drawn by venepuncture after an overnight fast and stored at below 2–8 °C to prepare for assay in the following way. Uric acid concentration was measured by the direct enzymatic method, in which UA was oxidized by uricase coupled with peroxidase. Serum UA was measured using a Clinical Analyzer 7180-ISE (Hitachi High-Technologies, Tokyo, Japan). In our hospital, the normal range of serum UA values is 150–360 μmol/L in women and 210–430 μmol/L in men.

Statistical analysis

All statistical analyses were performed using the Statistical Program for Social Sciences (SPSS) statistical software (version 11.0, Chicago, IL, USA). All the data in this study are presented as mean ± SD. Statistical significance was set at $P < 0.05$. The effect of age and gender on serum UA levels of different groups were analysed by covariance analysis. The comparison between serum UA levels of the MS patients and OND or the control subjects was performed using covariance analysis with age as the covariant. Since serum UA levels have been shown to be dependent on gender, in order to eliminate the effect of gender, patients with each group were divided into two subgroups according to the gender. Covariance analysis was also used to compare serum UA levels of males and females with MS, OND or the control subjects with age as the covariant. Furthermore, to explain the effect of different ages of females, serum UA levels of women of ages from 18 to 50 were analysed by covariance analysis with age as the covariant.

Results

In this study, the average serum UA level of all participants was 252.7 ± 108.2 μmol/L. Serum UA levels of patients with MS were significantly lower (239.4 ± 107.4 μmol/L) when compared with patients having TIA (344.6 ± 130.6 μmol/L, $P = 0.000$), cerebral hemorrhage (311.9 ± 104.7 μmol/L, $P = 0.000$), cerebral infarction (291.3 ± 101.6 μmol/L, $P = 0.014$) and the healthy control group (312.1 ± 92.8 μmol/L, $P = 0.000$). However, serum UA levels of patients with MS were significantly higher when compared with the patients with cryptococcus meningitis or meningoencephalitis (178.9 ± 107.0 μmol/L, $P = 0.000$) and tuberculous meningitis or meningoencephalitis patients (175.7 ± 99.9 μmol/L, $P = 0.000$). In addition, there was no significant difference in the serum UA levels between patients with MS and those with facial neuritis (267.0 ± 85.0 μmol/L, $P = 0.284$), viral meningitis or encephalitis (248.9 ± 104.2 μmol/L, $P = 0.856$), polymyositis or dermatomyositis patients (241.6 ± 78.5 μmol/L, $P = 0.792$), myasthenia gravis (237.2 ± 76.3 μmol/L, $P = 0.534$), subarachnoid hemorrhage (236.5 ± 105.7 μmol/L, $P = 0.404$), migraine (229.2 ± 115.6 μmol/L, $P = 0.170$), Guillain–Barre syndrome (224.2 ± 67.3 μmol/L, $P = 0.124$) and myelitis patients (221.5 ± 113.1 μmol/L, $P = 0.065$) (Table 2 and Figure 1).

In healthy controls as well as diseases such as MS, facial neuritis, viral meningitis or encephalitis,

Table 2 Serum UA levels in MS patients, patients with OND and healthy control group

Patients	Mean ± SD (μmol/L)	Range (μmol/L)	*P**
MS	239.4 ± 107.4	41.2–623.4	
TIA	344.6 ± 130.6	76.8–663.8	0.000
Cerebral hemorrhage	311.9 ± 104.7	83.5–551.0	0.000
Healthy control group	312.1 ± 92.8	59.6–536.4	0.000
Cerebral infarction	291.3 ± 101.6	80.4–533.0	0.014
Facial neuritis	267.0 ± 85.0	108.5–472.4	0.284
Viral meningitis or encephalitis	248.9 ± 104.2	110.3–510.4	0.856
Pulmonary tuberculosis	244.4 ± 99.9	46.3–496.2	0.420
Polymyositis or dermatomyositis	241.6 ± 78.5	10.8–465.7	0.792
Myasthenia gravis	237.2 ± 76.3	47.5–377.3	0.534
Subarachnoid hemorrhage	236.5 ± 105.7	66.2–496.4	0.404
Migraine	229.2 ± 115.6	87.6–489.7	0.170
Guillain–Barre syndrome	224.2 ± 67.3	87.3–339.6	0.124
Myelitis	221.5 ± 113.1	41.4–668.8	0.065
Cryptococcus meningitis or meningoencephalitis	178.9 ± 107.0	27.0–429.1	0.000
Tuberculous meningitis or meningoencephalitis	175.7 ± 99.9	45.4–475.5	0.000

*MS versus different types of OND or healthy control group.

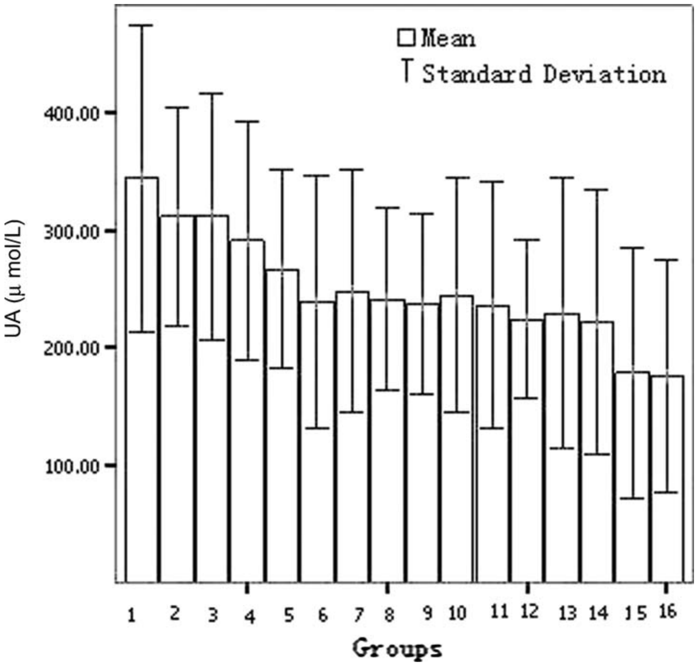

Figure 1 Distribution of UA levels in serum samples of patients with MS and OND. 1: transient ischemia attack, 2: healthy control group, 3: cerebral hemorrhage, 4: cerebral infarction, 5: facial neuritis, 6: MS, 7: viral meningitis or encephalitis, 8: polymyositis or dermatomyositis, 9: myasthenia gravis, 10: pulmonary tuberculosis, 11: subarachnoid hemorrhage, 12: Guillain–Barre syndrome, 13: migraine, 14: myelitis, 15: cryptococcus meningitis or meningoencephalitis, 16: tuberculous meningitis or meningoencephalitis.

myasthenia gravis, pulmonary tuberculosis and myelitis, women had significant lower serum UA levels than men ($P < 0.05$). Women had lower serum UA levels than men in other groups, but the difference was not statistically significant (Table 3 and Figure 2). Comparison of UA levels between female and male migraine patients was not performed due to the small number of male migraine cases (five cases).

In addition, we found that serum UA levels of female patients with MS were significantly different from those of female patients with cryptococcus meningitis or meningoencephalitis, tuberculous meningitis or meningoencephalitis, TIA and the healthy control group ($P < 0.05$). Serum UA levels of male patients with MS were also significantly different from those of male patients with cryptococcus meningitis or meningoencephalitis, tuberculous meningitis or meningoencephalitis, TIA and the healthy control group ($P < 0.05$) (Table 3 and Figure 2).

To explain the effect of different ages of females, serum UA levels of women of ages from 18 to 50 were analysed (Table 4 and Figure 3). Serum UA levels of female MS patients were significantly lower compared with those of healthy control women ($P = 0.027$), but significantly higher than those of female patients with tuberculous meningitis or meningoencephalitis ($P = 0.045$) and cryptococcus meningitis or meningoencephalitis ($P = 0.000$). The comparison of UA concentrations between patients with TIA and cerebral hemorrhage was not conducted, because female TIA patients were older than 50 years and only three females with cerebral hemorrhage were aged range between 18 and 50 years.

To investigate the effect of tuberculosis on the levels of serum UA, we compared serum UA levels of pulmonary tuberculosis patients with those of MS patients and the patients with tuberculous meningitis or meningoencephalitis. The patients with tuberculous meningitis or meningoencephalitis had significantly lower serum UA levels compared with patients with MS ($P = 0.000$). In contrast, patients with pulmonary tuberculosis had low serum UA levels, but the difference was not statistically significant when compared with patients with MS ($P = 0.765$).

Among MS patients, those with no MRI activity had low serum UA levels (240.2 ± 117.9 μmol/L), but the difference was not statistically significant when compared with patients with MRI activity (251.1 ± 107.7 μmol/L; $P = 0.673$) (Table 5). UA levels were mildly lower in patients with shorter disease duration (>5 years) (214.4 ± 88.7 μmol/L) than those in patients with longer duration of MS (≤5 years) (258.1 ± 116.6 μmol/L; $P = 0.033$)

Table 3 Serum UA levels in male and female MS, OND patients and healthy controls

Patients	Male (μmol/L)	Female (μmol/L)	*P*1*	*P*2[a]	*P*3[b]
MS	281.6 ± 132.5	214.0 ± 79.9	0.001		
Migraine	378.3 ± 95.4	195.3 ± 91.2	0.220		
Healthy control group	367.8 ± 93.2	260.0 ± 54.5	0.000	0.001	0.012
Transient ischemia attack	362.6 ± 122.2	305.6 ± 145.1	0.216	0.000	0.011
Cerebral hemorrhage	330.1 ± 95.3	276.8 ± 116.2	0.111	0.059	0.068
Cerebral infarction	304.4 ± 106.4	271.9 ± 92.2	0.150	0.301	0.018
Facial neuritis	291.0 ± 85.0	244.5 ± 79.8	0.033	0.709	0.296
Viral meningitis or encephalitis	271.2 ± 94.1	216.1 ± 111.3	0.030	0.647	0.760
Myasthenia gravis	267.0 ± 64.3	204.4 ± 76.4	0.006	0.596	0.624
Polymyositis or dermatomyositis	266.3 ± 76.5	224.6 ± 76.5	0.067	0.577	0.672
Pulmonary tuberculosis	265.3 ± 101.7	209.3 ± 88.0	0.036	0.477	0.714
Subarachnoid hemorrhage	260.1 ± 113.3	213.9 ± 95.1	0.155	0.427	0.580
Myelitis	253.8 ± 124.0	178.6 ± 81.5	0.031	0.284	0.111
Guillain–Barre syndrome	233.1 ± 67.0	205.1 ± 66.5	0.219	0.051	0.812
Cryptococcus meningitis	190.7 ± 101.0	144.4 ± 120.2	0.150	0.000	0.006
Tuberculous meningitis or meningoencephalitis	180.0 ± 103.5	170.9 ± 95.5	0.728	0.000	0.000

[a]Male patients with different types of OND or healthy control group versus male patients with MS.
[b]Female patients with different types of OND or healthy control group versus female patients with MS.
*Male versus female in each group.

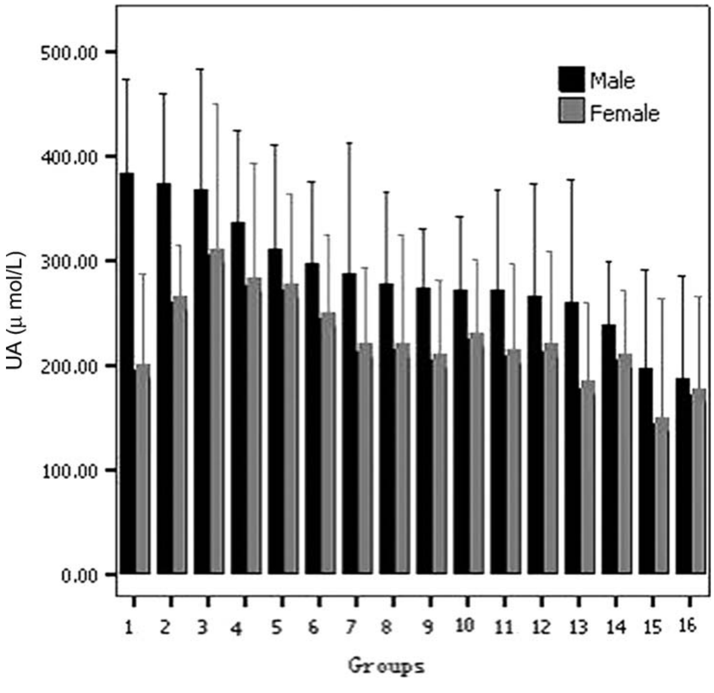

Figure 2 Serum UA levels of men and women in each group. 1: migraine, 2: healthy control group, 3: transient ischemia attack, 4: cerebral hemorrhage, 5: cerebral infarction, 6: facial neuritis, 7: MS, 8: viral meningitis or encephalitis,9: myasthenia gravis, 10: polymyositis or dermatomyositis, 11: pulmonary tuberculosis, 12: subarachnoid hemorrhage, 13: myelitis, 14: Guillain–Barre Syndrome, 15: cryptococcus meningitis or meningoencephalitis, 16: tuberculous meningitis or meningoencephalitis.

(Table 5). Patients with severe disabling disease (EDSS ≥ 3.5) (217.9 ± 111.0 μmol/L) had low serum UA levels, but the difference was not statistically significant compared with those having less disabling disease (EDSS < 3.5) (254.9 ± 102.8 μmol/L; $P = 0.072$) (Table 4). We divided patients with different clinical types of MS. RR-MS patients did not have significantly different serum UA levels ($n = 78$, 239.5 ± 106.9 μmol/L) compared with PP-MS or primary progressive–remitting

Table 4 Serum UA levels of women of ages from 18 to 50

Patients	Number of patients	Mean of ages	UA (μmol/L)	*P**
MS	58	32.8	216.1 ± 80.1	
Healthy control group	42	36.8	253.7 ± 52.4	0.027
Cerebral infarction	22	43.0	245.5 ± 96.7	0.197
Pulmonary tuberculosis	15	31.3	225.4 ± 86.8	0.684
Viral meningitis or encephalitis	19	27.8	213.1 ± 116.0	0.923
Subarachnoid hemorrhage	6	39.3	200.4 ± 62.5	0.628
Myasthenia gravis	16	34.6	196.2 ± 68.2	0.376
Guillain–Barre syndrome	9	29.2	192.8 ± 75.3	0.439
Polymyositis or dermatomyositis	16	35.9	192.5 ± 73.8	0.292
Facial neuritis	9	30.1	191.2 ± 47.5	0.402
Migraine	16	39.9	184.5 ± 69.6	0.158
Myelitis	15	35.7	180.8 ± 84.3	0.128
Tuberculous meningitis or meningoencephalitis	17	33.2	171.3 ± 86.5	0.045
Cryptococcus meningitis or meningoencephalitis	12	33.8	122.6 ± 112.2	0.000

*MS versus OND in each group.

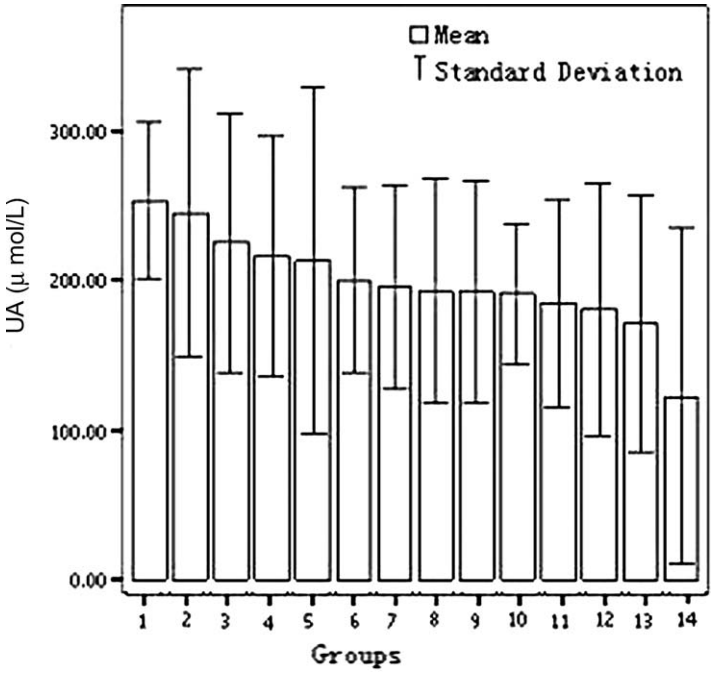

Figure 3 Serum UA levels of women of ages from 18 to 50. 1: healthy control group, 2: cerebral infarction, 3: pulmonary tuberculosis, 4: MS, 5: viral meningitis or encephalitis, 6: subarachnoid hemorrhage, 7: myasthenia gravis, 8: Guillain–Barre syndrome, 9: polymyositis or dermatomyositis, 10: facial neuritis, 11: migraine, 12: myelitis, 13: tuberculous meningitis or meningoencephalitis, 14: cryptococcus meningitis or meningoencephalitis.

(PR)-MS patients (n = 24, 240.1 ± 106.6 μmol/L; P = 0.982).

Discussion

In this study, serum UA levels of patients with MS were significantly lower when compared with patients with TIA, cerebral hemorrhage, cerebral infarction and the healthy control group. However, serum UA levels of patients with MS were significantly higher when compared with the patients with cryptococcus meningitis or meningoencephalitis and tuberculous meningitis or meningoencephalitis patients. In addition, there was no significant difference of serum UA levels between

Table 5 Serum UA levels in patients with MS

Variables	Mean ± SD (μmol/L)	Range (μmol/L)	P^a
Disease duration			
≤ 5 years, *n* = 64	258.1 ± 116.7	41.2–623.4	
> 5 years, *n* = 48	214.4 ± 88.7	63.5–519.1	0.033
MRI activity			
Active, *n* = 31	251.1 ± 107.7	41.2–623.4	
Inactive, *n* = 53	240.1 ± 117.9	67.3–564.0	0.673
EDSS			
< 3.5, *n* = 65	254.9 ± 102.8	41.2–564.0	
≥ 3.5, *n* = 47	217.9 ± 111.0	63.5–623.4	0.072
Sex			
Female, *n* = 70	214.0 ± 79.9	41.2–444.2	
Male, *n* = 42	281.6 ± 132.5	63.5–623.4	0.001
Type			
RR-MS, *n* = 78	239.5 ± 106.9	41.2–623.4	
PP-MS or PR-MS, *n* = 24	240.1 ± 106.6	107.3–618.8	0.982

[a]Compared UA levels between two subgroup of MS.
Correlation of serum UA levels with disease duration, MRI activity, disability, sex and type of MS. Values represent the mean ± S.D.

patients with MS and those with facial neuritis, viral meningitis or encephalitis, polymyositis or dermatomyositis patients, myasthenia gravis, subarachnoid hemorrhage, migraine, Guillain–Barre syndrome and myelitis patients. Our data do not support previous studies [3–5] that patients with MS have lower serum UA levels than all OND. UA levels are likely to differ depending on the disease process.

We have demonstrated for the first time that serum UA levels in patients with cryptococcus meningitis or meningoencephalitis and tuberculous meningitis or meningoencephalitis patients were significantly lower compared with those of MS patients. In addition, we found that patients with tuberculous meningitis or meningoencephalitis had significantly lower serum UA levels compared with patients having MS and pulmonary tuberculosis but, in contrast, the serum UA levels of patients with pulmonary tuberculosis were not different from those of patients with MS. These results suggest that tuberculosis may not have a significant effect on UA serum levels. The lower serum UA levels may be due to the consumption of UA by radical interaction and damaged tissue [13]. In addition, UA may deposit in sites of inflammation, and Langemann *et al.* [14] found that UA was increased in plaques of white matter from patients with MS. In this case, patients with low serum UA levels may predispose toward the development of MS and other CNS inflammatory diseases [13]. Taken together, our data may favor the view that the patients with cryptococcus meningitis or meningoencephalitis and tuberculous meningitis or meningoencephalitis have more severe inflammation or damage of blood–brain barrier than patients with MS.

In this regard, the lack of any significant difference of serum UA levels between patients with MS and those with facial neuritis, viral meningitis or encephalitis, pulmonary tuberculosis, polymyositis or dermatomyositis, myasthenia gravis, subarachnoid hemorrhage, migraine, Guillain–Barre syndrome and myelitis patients suggest that these diseases may have levels of inflammation or blood–brain barrier injury levels similar to MS. As a strong scavenger of PN, UA treatment has been found to suppress the MS inflammatory cascade, blood–brain barrier permeability changes, and CNS tissue damage and death in animal models of MS [15]. Similarly, increasing the concentration of UA might also help to prevent the secondary cellular damage of spinal-cord injury and promote the recovery of motor function, according to a new study by Scott *et al.* [16]. The protective effect of UA has already been documented in a rat model of pneumococcal meningitis [17]. Our data indicate that elevated UA serum levels may be associated with reduced CNS inflammation and tissue damage. The results, therefore, are the first to suggest that patients with some OND and lower levels of serum UA are predisposed to tissue injury than those with MS and that elevated UA maybe beneficial to these neurological diseases. Nevertheless, further studies are necessary to clarify the underlying mechanisms.

We also found that TIA, cerebral hemorrhage, cerebral infarction and the healthy control group have significantly higher serum UA levels than MS patients. Several large studies have identified an elevated serum UA concentration as a predictor of cardiovascular events such as myocardial infarction [18–20]. Independent from other prognostic

factors, higher serum UA levels predicted poor outcome and higher vascular event rates [21]. However, the role of UA in cardiovascular diseases still remains controversial while the administration of UA has been shown to be beneficial to acute ischemic stroke in rats [22]. Most of studies suggest a harmful role of elevated UA levels in cardiovascular diseases. To illustrate this point, UA has been found to stimulate the synthesis of monocyte chemoattractant protein-1 by rat vascular smooth muscle cells, which is known to have a key role in stimulating macrophage infiltration in atherosclerotic vessels [23]. Furthermore, there is strong evidence that an elevated UA is injurious rather than protective in subjects with acute stroke [21,24].

Serum UA levels have been shown to be dependent on both age and gender. In this study, we further divided each group into two subgroups of female and male based on gender. We found that women had lower mean serum UA values when compared with men, which is consistent with previous studies [8,9,20]. We also found that UA levels in female MS patients were significantly lower than the female patients with TIA, cerebral infarction and the healthy control group and were significantly higher than the female patients with cryptococcus meningitis or meningoencephalitis and tuberculous meningitis or meningoencephalitis. We also found that serum UA levels of male patients with MS were significantly different from those of male patients with cryptococcus meningitis or meningoencephalitis, tuberculous meningitis or meningoencephalitis, TIA and the healthy control group. The serum UA levels of pre-pubescent and post-menopausal women are more or less equivalent to those of similarly aged males, while during the childbearing years, the serum UA levels of women are significantly reduced. In this study, the serum UA levels of 18 to 50 aged female patients with MS were significantly lower than those of healthy women, but significantly higher than female patients with tuberculous meningitis or meningoencephalitis and cryptococcus meningitis or meningoencephalitis. Although several studies have attempted to explore why female MS patients tend to have lower UA level [8,9,20], there is not any satisfactory explanation. Nevertheless, in relation to serum UA levels, which are highly sex- and age-dependent, Maxwell and Bruinsma proposed [25] that a rise in such levels was associated with cardiovascular disease as a consequence of an impairment of vascular NO release. They pointed out that this might be due to the ability of NO to modulate UA production via xanthine metabolism. Peinado [26] considered that decrease of nitroso compound (NOx) in men of the 51–65 age group could explain this finding. However, this is not the case in women, in which NOx levels did not change or even increased in the over 50-year-old group.

Previous studies reported that UA levels, although decreased in MS patients, were not correlated with disease activity, duration, disability or course [5,6]. In the present study, UA level were also not correlated with MRI activity, disability or subtypes of MS, which is partially consistent with these reports. The absence of any relationship between UA levels and MRI activity in this study suggests that the role of UA in disease activity is far from clear. Although Hooper *et al.* [15] have shown in a mouse model of MS that a reduced antioxidant reserve may coexist with the increased consumption of UA as a scavenger [15], it remains uncertain whether the low values of UA of patients with MS are a cause or a consequence of the disease activity [6]. However, we found that UA levels were independently correlated with duration and gender of MS. Due to the correlation between UA levels and duration of MS, future therapies targeted at increasing UA levels in MS should start early in the course of the disease in order to promote neuroprotection and to prevent axonal injury and irreversible tissue damage [6].

To our knowledge, this is the first study showing that different types of OND have significantly different levels of serum UA. Previous studies have found that patients with MS have lower serum UA levels than the healthy control subjects or patients with OND [3,4,5]. Interestingly, in this study, the UA levels in patients with MS could be either lower or higher than those in patients with OND, depending on different types of OND. Administration of UA or its precursor inosine to patients with reduced UA serum levels should be considered as a replacement therapy to replenish antioxidant capacity in a UA-deficient state. Although elevated UA serum levels were associated with reduced CNS inflammation and tissue damage [4,15–17], elevated UA levels were injurious rather than protective in subjects with acute stroke [21,24]. Therefore, UA possesses biphasic effects: neuroprotective and injurious. Our studies may help physicians to deal with conditions with abnormal UA levels.

References

1. **Bagasra O, Michaels FH, Zheng YM, Bobroski LE, Spitsin SV, Fu ZF** *et al.* Activation of the inducible form of nitric oxide synthase in the brain of the patients with multiple sclerosis. *Proc Natl Acad Sci USA* 1995; **92**: 12041–45.
2. **Van der Veen RC, Hinton DR, Incardonna F, Hofman FM.** Extensive peroxynitrite activity during progressive stages of central nervous system inflammation. *J Neuroimmunol* 1997; **77**: 1–7.

3. **Hooper DC, Bagasra O, Marini JC, Zborek A, Ohnishi ST, Kean R** *et al.* Prevention of experimental allergic encephalo-myelitis by targeting nitric oxide and peroxynitrite: implications for the treatment of multiple sclerosis. *Proc Natl Acad Sci USA* 1997; **94**: 2528–33.
4. **Hooper DC, Spitsin S, Kean RB, Champion JM, Dickson GM, Chaudhry I** *et al.* Uric acid, a natural scavenger of peroxynitrite, in experimental allergic encephalo-myelitis and multiple sclerosis. *Proc Natl Acad Sci USA* 1998; **95**: 675–80.
5. **Sotgiu S, Pugliatti M, Sanna A, Sotgiu A, Fois ML, Arru G** *et al.* Serum uric acid and multiple sclerosis. *Neurol Sci* 2002; **23**: 183–8.
6. **Rentzos M, Nikolaou C, Anagnostouli M, Rombosa A, Tsakanikas K, Economoub M** *et al.* Serum uric acid and multiple sclerosis. *Clin Neurol Neurosurg* 2006; **108**: 527–31.
7. **Miller A, Glass-Marmor L, Abraham M, Grossman I, Shapiro S, Galboiz Y.** Bio-markers of disease activity and response to therapy in multiple sclerosis. *Clin Neurol Neurosurg* 2004; **106**: 249–54.
8. **Drulovic J, Dujmovic I, Stojsavljevic N, Mesaros A, Andjelkovic S, Miljkovic D** *et al.* Uric acid levels in sera from patients with multiple sclerosis. *J Neurol* 2001; **248**: 121–6.
9. **Toncev G, Milicic B, Toncev S, Samardzic G.** Serum uric acid levels in multiple sclerosis patients correlate with activity of disease and bloodbrain barrier dysfunction. *Ear J Neurol* 2002; **9**: 221–6.
10. **Poser CM, Paty DW, Scheinberg L, McDonald WI, Davis FA, Ebers GC** *et al.* New diagnostic criteria for multiple sclerosis: guidelines for research protocols. *Ann Neurol* 1983; **13**: 227–31.
11. **McDonald WI, Compston A, Edan G, Goodkin D, Hartung HP, Lublin FD** *et al.* Recommended diagnostic criteria for multiple sclerosis: guidelines from the international panel on the diagnosis of multiple sclerosis. *Ann Neurol* 2001; **50**: 121–7.
12. **Kurtzke JF.** Rating neurologic impairment in multiple sclerosis: an expanded disability status scale (EDSS). *Neurology* 1983; **33**: 1444–52.
13. **Kean RB, Spitsin SV, Mikheeva T, Scott GS, Hooper DC.** The peroxynitrite scavenger uric acid prevents inflammatory cell invasion into the central nervous system in experimental allergic encephalomyelitis through maintenance of blood-central nervous system barrier integrity. *J Immunol* 2000; **165**: 6511–8.
14. **Langemann H, Kabiersch A, Newcombe J.** Measurement of low-molecular-weight antioxidants, uric acid, tyrosine and tryptophan in plaques and white matter from patients with multiple sclerosis. *Eur Neurol* 1992; **32**(5): 248–52.
15. **Hooper D, Scott G, Zborek A, Mikheeva T, Kean R, Koprowski H** *et al.* Uric acid, a peroxynitrite scavenger, inhibits CNS inflammation, blood-CNS barrier permeability changes, and tissue damage in a mouse model of multiple sclerosis. *FASEB J* 2000; **14**: 691–8.
16. **Scott GS, Cuzzocrea S, Genovese T, Koprowski H, Hooper DC.** Uric acid protects against secondary damage after spinal cord injury. *PNAS* 2005; **102**: 3483–8.
17. **Kastenbauer S, Koedel U, Pfister HW.** Role of peroxynitrite as a mediator of pathophysiological alterations in experimental pneumococcal meningitis. *J Infect Dis* 1999; **180**: 1164–70.
18. **Abbott RD, Brand FN, Kannel WB, Castelli WP.** Gout and coronary heart disease: the Framingham study. *J Clin Epidemiol* 1988; **41**: 237–42.
19. **Culleton BF, Larson MG, Kannel WB, Levy D.** Serum uric acid and risk of cardiovascular disease and mortality: the Framingham Heart Study. *Ann Intern Med* 1999; **131**: 7–13.
20. **Fang J, Alderman MH.** Serum uric acid and cardiovascular mortality: the NHANES I epidemiologic follow-up study, 1971–1992: National Health and Nutrition Examination Survey. *JAMA* 2000; **283**: 2404–10.
21. **Weir CJ, Muir SW, Walters MR, Lees KR.** Serum urate as an independent predictor of poor outcome and future vascular events after acute stroke. *Stroke* 2003; **34**: 1951–6.
22. **Yu ZF, Bruce-Keller AJ, Goodman Y, Mattson MP.** Uric acid protects neurons against excitotoxic and metabolic insults in cell culture, and against focal ischemic brain injury in vivo. *J Neurosci Res* 1998; **53**: 613–25.
23. **Kanellis J, Watanabe S, Li JH, Kang DH, Li P, Nakagawa T** *et al.* Uric acid stimulates monocyte chemoattractant protein-1 production in vascular smooth muscle cells via mitogen-activated protein kinase and cyclooxygenase-2. *Hypertension* 2003; **41**: 1287–93.
24. **Kanellis J, Johnson RJ.** Elevated uric acid and ischemic stroke: Accumulating evidence that it is injurious and not neuroprotective. *Stroke* 2003; **34**: 1956–7.
25. **Maxwell AJ, Bruinsma KA.** Uric acid is closely linked to vascular nitric oxide activity. Evidence for mechanism of association with cardiovascular disease. *J Am Coll Cardiol* 2001; **38**: 1850–8.
26. **Peinado MA, Lopez-Ramos JC, Camacho MV, Molina FJ, Martínez-Romero R, Hernández R** *et al.* Age and sex related serum changes in nitric oxide: Correlations with serological markers. *Int J Cardiol* 2007, DOI: 10.1016/j.ijcard.2006.08.032

Original Paper

NeuroImmunoModulation

Neuroimmunomodulation 2012;19:43–49
DOI: 10.1159/000327727

Received: October 27, 2010
Accepted after revision: March 16, 2011
Published online: November 7, 2011

Antioxidant Status of Bilirubin and Uric Acid in Patients with Myasthenia Gravis

Peng Fuhua[a] Deng Xuhui[a] Zhou Zhiyang[c] Jiang Ying[a] Yang Yu[a] Tan Feng[d]
Liu Jia[a] Gu Lijia[b] Hu Xueqiang[a]

Departments of [a]Neurology and [b]Cardio-Thoracic Surgery, Third Affiliated Hospital of Sun Yat-sen University and [c]Department of Radiology, Sixth Affiliated Hospital of Sun Yat-sen University, Guangzhou, and [d]Department of Neurology, Foshan Chinese Medicine Hospital, Foshan, China

Key Words
Myasthenia gravis · Bilirubin · Uric acid · Antioxidant

Abstract
Oxidative stress and changes in antioxidant status have been implicated in the pathogenesis of inflammatory and autoimmune diseases, and free radicals can cause considerable damage to the acetylcholine receptors. 388 individuals, including 97 patients with myasthenia gravis (MG), 135 patients with multiple sclerosis (MS) and 156 healthy controls, were assessed for serum levels of bilirubin and uric acid (UA), in order to determine the levels of these natural antioxidants in the serum. We found that serum UA levels in patients with MG were significantly lower (266.03 ± 93.09 μmol/l) compared with those of the healthy control group (338.87 ± 107.10 μmol/l, p = 0.001). However, there was no significant difference of serum UA levels between patients with MG and those with MS (p = 0.071). We also found that serum levels of total, direct and indirect bilirubin in patients with MG were significantly lower, compared with those in the healthy control group, whether male or female. From this study, we conclude that serum levels of bilirubin and UA are lower in MG patients.

Copyright © 2011 S. Karger AG, Basel

Introduction

Reactive oxygen species (ROS) are oxygen-based molecules with high chemical reactivity. ROS include free radicals (superoxide and hydroxyl radicals) and nonradical species (hydrogen peroxide), which can be produced even at resting conditions in a number of ways. Despite their different structures, free radicals share similar mechanisms of jeopardizing the body's cells and tissues through protein, DNA and lipid damage [1]. Enzymatic and nonenzymatic antioxidant defense systems scavenge or decrease ROS levels, thereby maintaining an appropriate cellular redox balance. Alterations of this normal balance result from elevated ROS production and/or decreased antioxidant levels leading to a state of oxidative stress and enhanced susceptibility of membranes and biological molecules to react with free radicals [2]. Uric acid (UA), a scavenger of peroxynitrite [3] and a natural antioxidant, accounts for up to 60% of the free radical scavenging activity in human blood [4]. Bilirubin, an endproduct of heme metabolism converted from biliver-

The first three authors contributed equally to this paper.

KARGER

Fax +41 61 306 12 34
E-Mail karger@karger.ch
www.karger.com

© 2011 S. Karger AG, Basel
1021–7401/12/0191–0043$38.00/0

Accessible online at:
www.karger.com/nim

Fuhua Peng
Department of Neurology, Third Affiliated Hospital of Sun Yat-sen University
600 Tianhe Road
Guangzhou, Guangdong Province 510630 (China)
Tel. +86 20 8525 3295, E-Mail pfh93@21cn.com

Downloaded by:
University of Pittsburgh
198.143.32.65 - 8/15/2015 5:42:01 AM

din by biliverdin reductase (BVR), was long thought to be merely a cytotoxic waste product. However, most current studies on the physiological functions of bilirubin focus on its antioxidant effects. Bilirubin suppresses oxidation more strongly than many other antioxidants, including α-tocopherol (vitamin E), superoxide dismutase and catalase, especially under pathological conditions [5, 6]. In summary, UA and bilirubin in the serum can both have antioxidant effects and reduce global oxidative stress.

In a range of neuromuscular diseases, oxidative free radicals are suspected in damage caused to neuromuscular structures, occurring when these free radicals are present at elevated concentrations or when endogenous antioxidative protection systems are impaired [7]. However, these postulated mechanisms have not been understood, and little data are available on human muscle diseases [8], such as myasthenia gravis (MG), an autoimmune neuromuscular disease leading to fluctuating muscle weakness and fatigability. The weakness in MG is caused by circulating antibodies that block acetylcholine receptors (AChR) at the postsynaptic neuromuscular junction [9], thereby inhibiting the stimulating effect of the neurotransmitter acetylcholine.

Oxidative damage is known to be involved in inflammatory and autoimmune-mediated tissue destruction [10]. It is well documented that free radicals can continuously cause considerable damage to the AChR [11] and that antioxidants play a major role in protecting free radical-mediated receptor damage [11]. However, the relationship of MG and antioxidant status has not fully been elucidated. Here we conducted a hospital-based study which aimed to determine possible differences in antioxidant status by evaluating serum UA and bilirubin levels of patients with MG.

Patients and Methods

Patients

Serum samples were collected from 388 individuals comprising 97 patients with MG, 135 patients with multiple sclerosis (MS) and 156 healthy controls. Demographic and clinical characteristics of patients and healthy controls are presented in table 1.

All patients had definitive diagnoses according to disease-specific diagnostic criteria. The diagnosis of MG was based on the association of typical history and signs together with positive response to neostigmine intravenously and abnormal decrement (>11%) of the third to fifth compound muscle action potential on low-rate repetitive nerve stimulation. The myasthenic condition was graded according to the modified Osserman classification as follows: I = ocular signs and symptoms, IIa = mild generalized weakness, IIb = moderate generalized weakness, III = severe generalized weakness, respiratory dysfunction, or both. All MS patients had definitive diagnoses according to the criteria of Poser et al. [12] or McDonald et al. [13]. Exclusion criteria were treatment with steroids before UA and bilirubin measurements, liver disease, abnormal ranges of glutamate-pyruvate transaminase (GPT) and glutamic-oxaloacetic transaminase (GOT) concentrations, as well as subjects with diabetes or renal failure.

Table 1. Demographic characteristics of healthy controls and patients with MS and MG

Subjects	All patients	Male	Female	Age, years
UA groups				
Patients with MG	77	40	37	32.91 ± 20.20
Patients with MS	127	48	79	34.41 ± 14.19
Healthy controls	133	73	60	46.99 ± 16.47
Bilirubin groups				
Patients with MG	92	41	51	33.30 ± 19.19
Patients with MS	133	46	87	35.91 ± 15.21
Healthy controls	154	87	67	48.82 ± 17.47

UA and Bilirubin Measurements

Venous blood was collected for the measurement of total serum UA and bilirubin concentrations in the morning after an overnight fast. Serum UA concentrations, total bilirubin (Tbil) concentrations, direct bilirubin (Dbil) concentrations and indirect bilirubin (Ibil) concentrations were measured by an enzymatic method with bilirubin oxidase on a Clinical Analyzer 7180-ISE (Hitachi High-Technologies, Tokyo, Japan). Simultaneously, fasting blood glucose, blood urea nitrogen, creatinine, GPT concentrations (normal range: 3–35 U/l) and GOT concentrations (normal range: 14.5–40 U/l) were measured by an enzymatic method on the same analyzer. Individuals with abnormal serum GPT and GOT levels were excluded.

Statistical Analysis

All statistical analyses were performed using the Statistical Program for Social Sciences statistical software (version 17.0; SPSS Inc., Chicago, Ill., USA). All data in this study are presented as means ± SD. Statistical significance was set at $p < 0.05$. The effect on serum UA and bilirubin levels of different groups was analyzed by covariance analysis. The comparisons between serum UA and bilirubin levels of the patients with MG, MS and the control subjects were performed using covariance analysis with age as covariant. Since serum bilirubin levels have been shown to be dependent on gender, in order to eliminate the effect of gender, patients in each group were divided into two male and female subgroups based on their gender. Covariance analysis was also used to compare serum bilirubin levels of males and females with MG, MS and the control subjects with age as a covariant. Similarly, serum UA and bilirubin levels in different groups were compared with age as a covariant.

Downloaded by:
University of Pittsburgh
198.143.32.65 - 8/15/2015 5:42:01 AM

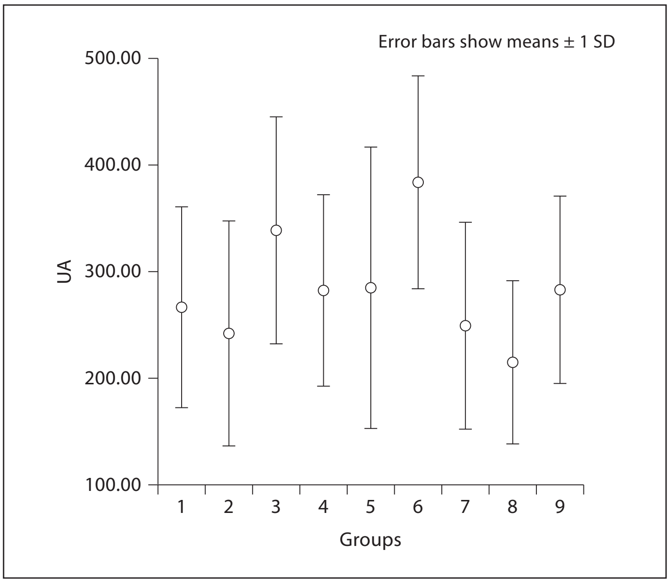

Fig. 1. Serum UA levels in patients with MG and MS and healthy control group. 1 = MG; 2 = MS; 3 = healthy control group; 4 = MG, males; 5 = MS, males; 6 = healthy control group, males; 7 = MG, females; 8 = MS, females; 9 = healthy control group, females.

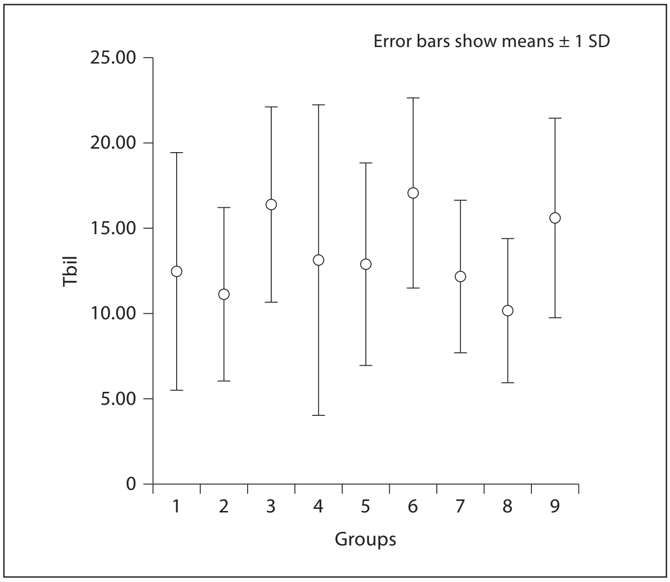

Fig. 2. Serum Tbil levels in patients with MG and MS and healthy control group. 1 = MG; 2 = MS; 3 = healthy control group; 4 = MG, males; 5 = MS, males; 6 = healthy control group, males; 7 = MG, females; 8 = MS, females; 9 = healthy control group, females.

Table 2. Serum UA levels in healthy controls and patients with MG and MS

Patients	Total	Male	Female	p^1	p^2	p^3	p^4
MG	266.03 ± 93.09	282.23 ± 89.54	248.52 ± 94.88				0.010
MS	240.79 ± 106.16	285.60 ± 130.97	213.56 ± 76.71	0.071	0.981	0.089	0.001
HC	338.87 ± 107.10	384.76 ± 99.66	283.05 ± 88.13	0.001	0.001	0.045	0.001

Data are presented as means ± SD in μmol/l; HC = healthy controls.
p^1 = MG versus either MS or HC; p^2 = male patients with MG versus male HC and patients with MS; p^3 = female patients with MG versus female HC and patients with MS; p^4 = male versus female in each group.

Results

The average serum UA level of all participants was 285.27 ± 112.44 μmol/l. Serum UA level of patients with MG was significantly lower (266.03 ± 93.09 μmol/l) compared with the healthy control group (338.87 ± 107.10 μmol/l, p = 0.001). However, there was no significant difference of serum UA levels between patients with MG and those with MS (240.79 ± 106.16 μmol/l, p = 0.071) (table 2; fig. 1). Serum UA levels have been shown to be dependent on gender. In this study, we further divided each group into female and male subgroups. In male subgroups, serum UA levels of patients with MG were significantly lower (282.23 ± 89.54 μmol/l) when compared with the male healthy control subgroup (384.76 ± 99.66 μmol/l, p = 0.001). There was no significant difference of serum UA levels between male patients with MG and those with MS (285.60 ± 130.97 μmol/l, p = 0.981) (table 2; fig. 1). In female subgroups, serum UA levels of patients with MG were mildly lower (248.52 ± 94.88 μmol/l) when compared with the female healthy control group (283.05 ± 88.13 μmol/l, p = 0.045). In addition, there was no significant difference of serum UA levels between female patients with MG and those with MS (213.56 ± 76.71, p = 0.089) (table 2; fig. 1).

Downloaded by:
University of Pittsburgh
198.143.32.65 - 8/15/2015 5:42:01 AM

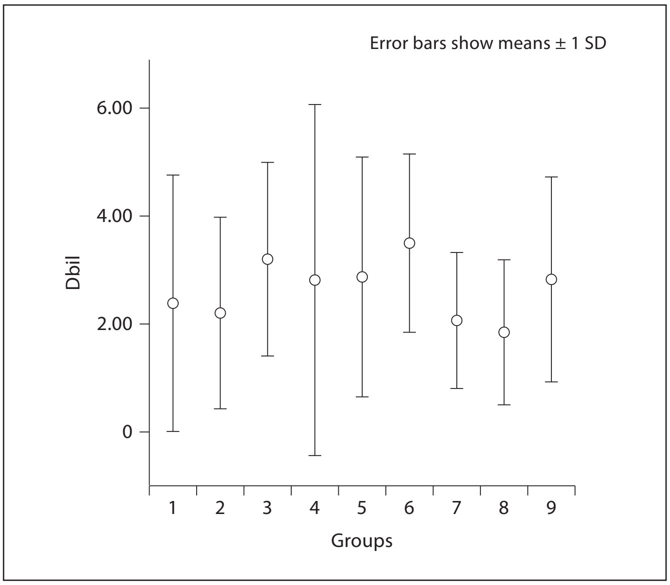

Fig. 3. Serum Dbil levels in patients with MG and MS and healthy control group. 1 = MG; 2 = MS; 3 = healthy control group; 4 = MG, males; 5 = MS, males; 6 = healthy control group, males; 7 = MG, females; 8 = MS, females; 9 = healthy control group, females.

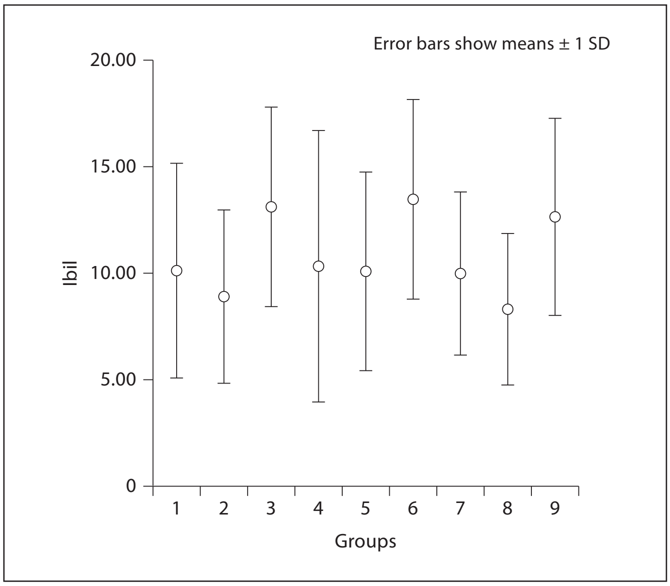

Fig. 4. Serum Ibil levels in patients with MG and MS and healthy control group. 1 = MG; 2 = MS; 3 = healthy control group; 4 = MG, males; 5 = MS, males; 6 = healthy control group, males; 7 = MG, females; 8 = MS, females; 9 = healthy control group, females.

Table 3. Serum bilirubin levels in healthy controls and patients with MG and MS

Patients	Total	Male	Female	p^1	p^2	p^3	p^4
MG							
Tbil	12.54 ± 6.93	13.16 ± 9.16	12.07 ± 4.45				0.285
Dbil	2.39 ± 2.37	2.81 ± 3.23	2.06 ± 1.25				0.040
Ibil	10.15 ± 5.03	10.32 ± 6.29	10.01 ± 3.79				0.608
MS							
Tbil	11.08 ± 5.06	12.93 ± 5.99	10.10 ± 4.20	0.049	0.695	0.061	0.010
Dbil	2.20 ± 1.75	2.87 ± 2.20	1.85 ± 1.34	0.401	0.952	0.564	0.004
Ibil	8.88 ± 4.02	10.05 ± 4.63	8.27 ± 3.54	0.030	0.622	0.033	0.038
HC							
Tbil	16.36 ± 5.76	17.01 ± 5.62	15.51 ± 5.86	0.001	0.003	0.008	0.077
Dbil	3.21 ± 1.79	3.50 ± 1.65	2.83 ± 1.90	0.012	0.164	0.090	0.020
Ibil	13.15 ± 4.64	13.51 ± 4.67	12.69 ± 4.60	0.001	0.002	0.007	0.198

Data are presented as means ± SD in μmol/l; HC = healthy controls.
p^1 = MG versus either MS or HC; p^2 = male patients with MG versus male HC and patients with MS; p^3 = female patients with MG versus female HC and patients with MS; p^4 = male versus female in each group.

The average serum levels of Tbil, Dbil and Ibil in all participants were 13.58 ± 6.28, 2.66 ± 1.98 and 10.92 ± 4.90 μmol/l, respectively. We found that serum levels of bilirubin fractions in patients with MG were significantly lower when compared with those of the healthy control group (p = 0.001, p = 0.012 and p = 0.001, respectively) (table 3; fig. 2–4). Serum levels of Tbil and Ibil in patients with MG were mildly higher when compared with the patients with MS (p = 0.049 and p = 0.030, respectively). However, serum levels of Dbil in patients with MG were

Downloaded by:
University of Pittsburgh
198.143.32.65 - 8/15/2015 5:42:01 AM

Table 4. Serum UA and bilirubin levels in patients with MG according to the modified Osserman classification

Subjects	Osserman I	Osserman IIa	Osserman IIb and III	p^1	p^2	p^3
UA groups						
Patients	35	24	18			
Uric acid	282.47 ± 104.66	258.41 ± 77.57	233.83 ± 101.16	0.275	0.064	0.393
Bilirubin groups						
Patients	46	26	20			
Tbil	11.39 ± 7.79	11.83 ± 5.59	12.93 ± 3.88	0.741	0.971	0.754
Dbil	2.11 ± 2.38	1.94 ± 1.24	2.49 ± 1.79	0.536	0.818	0.462
Ibil	9.28 ± 5.76	9.89 ± 4.73	10.42 ± 2.90	0.855	0.953	0.921

p^1 = Osserman I versus Osserman IIa; p^2 = Osserman I versus Osserman IIb and III; p^3 = Osserman IIa versus Osserman IIb and III.

not significantly higher when compared with the patients with MS (p = 0.401). Since serum bilirubin levels have been shown to be dependent on gender, each group was further divided into two subgroups according to gender in order to evaluate this effect (table 3; fig. 2–4). We found that serum levels of Tbil and Ibil in male patients with MG were significantly lower than those in the male healthy control group, a similar phenomenon found in female patients with MG and the female healthy control group (table 3; fig. 2–4). However, no difference was found when comparing serum levels of Dbil in male patients with MG with those in the male healthy control group, or when comparing Dbil in female patients with MG with the female healthy control group (table 3; fig. 2–4). In addition, no difference existed in the mean serum levels of Tbil, Dbil and Ibil in male and female patients with MG compared with those in male and female patients with MS.

In general, women had lower serum UA, Tbil, Dbil and Ibil levels than men. Women with MS had significantly lower serum Tbil, Dbil and Ibil levels than men with MS. Serum Dbil levels of female patients with MG and healthy controls were significantly lower than those of male patients with MG and healthy controls (table 3).

In order to examine the effect of MG grades on the serum levels of bilirubin and UA, patients in each group were divided into three subgroups according to the modified Osserman classification as follows: A subgroup = Osserman I, B subgroup = Osserman IIa, C subgroup = Osserman IIb and III (table 4). There were no significant differences of serum UA levels or bilirubin levels, whether total or fractionated, between groups of disease severity.

Discussion

We found that the serum level of UA in patients with MG was significantly lower than in the healthy control group. However, there was no significant difference between serum UA levels of patients with MS, another autoimmune-mediated neuropathic condition. Similar results were observed when the male and female cohorts were investigated separately. Low UA levels have previously been reported in MS [8–10], and our results suggest this is also the case in patients with MG. We also found that serum levels of Tbil, Dbil and Ibil in patients with MG were lower when compared with those with the healthy control groups, even when evaluating male and female groups separately. From this study, we conclude that serum levels of bilirubins and UA in MG patients are reduced. To our knowledge, this is the first report of an association of MG with antioxidant status of bilirubins and UA.

Acquired MG is a B cell-mediated autoimmune disorder, with a majority of patients having antibodies against AChR in the serum. AChR antibodies cause a loss of receptors, complement-mediated focal lysis of the post-synaptic membrane, and partial or complete inhibition of receptor function. A reduction in the number of AChR results in a characteristic pattern of progressively reduced muscle strength with repeated use of the muscle and recovery of muscle strength following a period of rest.

Free radicals also cause considerable damage to the AChR [11]. Furthermore, free radical-mediated receptor damage has been shown to be dose and time dependent [11]. In turn, ROS injure the body's cells and tissues

Downloaded by:
University of Pittsburgh
198.143.32.65 - 8/15/2015 5:42:01 AM

through a variety of mechanisms, including directly via damage to cellular lipids, proteins and DNA, and indirectly through stimulation of gene expression associated with apoptosis [1]. ROS also play a role in the underlying mechanisms of pyridostigmine-induced neurotoxicity by which pyridostigmine can induce apoptotic cell death in the rat brain and in cultured cortical neurons [14, 15]. Oxidative damage is already known to be involved in inflammatory and autoimmune-mediated tissue destruction. Imbalance between the production of antioxidant defense mechanisms and ROS can result in oxidative stress leading to metabolic impairment and cell death. Oxidative stress may be due to deficiency of antioxidants (such as glutathione, ascorbate or α-tocopherol) or overabundance of oxidant enzymes (superoxide dismutase, catalase or glutathione peroxidase) [16].

Skeletal muscle is strongly dependent upon oxidative metabolism, and corresponding antioxidant protection mechanisms are present. The antioxidant protection system of a muscle is in a dynamic equilibrium: a lack of one factor to a certain extent can be compensated by an increase in other factors [20]. Although muscle and nerve are reasonably well protected against active oxygen and related free radicals, environmental or inherited malfunctions can overpower their defenses. ROS have been shown to be involved in many neuropathies and myopathies [24].

We hypothesize that the decrease in UA and bilirubin levels of patients in this study may correlate with a relative inability of these patients to protect themselves against ROS. UA treatment has been shown to suppress the inflammatory cascade, and bilirubin also can suppress oxidation and has cytoprotectant function [5, 6, 21, 22]. Increasing evidence suggests that bilirubin possesses multiple biological activities, including potential immunomodulatory properties [23]. The potential therapeutic applications of antioxidants in free radical-related diseases led to the hypothesis of their use to slow down or reverse, for example, symptoms associated with neurodegenerative disorders, such as MS, Alzheimer's disease, Parkinson's disease or spongiform encephalopathies. Such effects may occur through blockade of pro-inflammatory cytokines, diminishing the resulting oxidative damage [25–27]. Therefore, future therapies targeted at increasing antioxidant levels should start early in the course of the disease in order to protect the AChR and prevent the progression of MG.

Previous studies in MS have reported a moderately low UA in remission stage and much lower UA in the relapsing stage [17, 18]. UA has been considered as a surrogate marker of MS activity. We speculate that the lower serum bilirubin and UA levels also present in MG may be due to the consumption of UA and bilirubin by radical interaction and damaged tissue in this disease, as in MS. Therefore, low serum levels of bilirubin and UA in MG patients may be associated with the pathophysiology of the disease. However, in the present study, there was no correlation between serum UA or bilirubin and disease grade according to the modified Osserman classification.

In the present study, the serum levels of Dbil in MG patients were significantly lower in women than in men. We also found that the serum levels of Tbil and Ibil in male patients with MG were significantly lower than those in male patients with MS and the healthy control group. Our findings are consistent with previous observations [19]. All women also had significantly lower serum UA levels than men. This lower serum antioxidant level in females may be one reason why the incidence of MG is higher in women than in men. Nevertheless, further studies would be necessary to confirm this observation.

This is the first study showing lower serum UA and bilirubin levels in patients with MG. Although it is unclear whether low serum UA level might be a cause or a consequence of the disease, it is possible that MG patients with low serum UA and bilirubin levels are unable to prevent against free radical toxicity which leads to the development of inflammation and destruction of the AChR. It is also possible that the inflammation occurring in MG leads to consumption of UA and bilirubin by scavenging excess free radicals, lowering UA and bilirubin levels as a consequence. As a replacement therapy to patients with reduced UA and bilirubin serum levels, administration of these substances or their precursors may be beneficial to patients with MG and should be studied further in randomized clinical trials.

Acknowledgements

The study was supported by the Technology Project of Guangdong Province (No. 2006B36004003), the Natural Science Foundation of Guangdong Province (No. 8151008901000104) and the Technology Project of Guangzhou City (No. 2060402).

Downloaded by:
University of Pittsburgh
198.143.32.65 - 8/15/2015 5:42:01 AM

References

1 Pauwels EK, Erba PA, Kostkiewicz M: Antioxidants: a tale of two stories. Drug News Perspect 2007;20:579–585.
2 Kurien BT, Scofield RH: Autoimmunity and oxidatively modified. Autoimmun Rev 2008; 7:567–573.
3 Hooper DC, Bagasra O, Marini JC, Zborek A, Ohnishi ST, Kean R, Champion JM, Sarker AB, Bobroski L, Farber JL, Akaike T, Maeda H, Koprowski H: Prevention of experimental allergic encephalo-myelitis by targeting nitric oxide and peroxynitrite: implications for the treatment of multiple sclerosis. Proc Natl Acad Sci USA 1997;94:2528–2533.
4 Ames BN, Cathcart R, Schwiers E, Hochstein P: Uric acid provides an antioxidant defense in humans against oxidant- and radical-caused aging and cancer: a hypothesis. Proc Natl Acad Sci USA 1981;78:6858–6862.
5 Stocker R, Yamamoto Y, McDonagh AF, Glazer AN, Ames BN: Bilirubin is an antioxidant of possible physiological importance. Science 1987;235:1043–1046.
6 Wu TW, Carey D, Wu J, Sugiyama H: The cytoprotective effects of bilirubin and biliverdin on rat hepatocytes and human erythrocytes and the impact of albumin. Biochem Cell Biol 1991;69:828–834.
7 Stuerenburg HJ: The roles of carnosine in aging of skeletal muscle and in neuromuscular diseases. Biochemistry (Mosc) 2000;65:862–865.
8 Mecocci P, Fanó G, Fulle S, MacGarvey U, Shinobu L, Polidori MC, Cherubini A, Vecchiet J, Senin U, Beal MF: Age-dependent increases in oxidative damage to DNA, lipids, and proteins in human skeletal muscle. Free Radic Biol Med 1999;26:303–308.
9 Conti-Fine BM, Milani M, Kaminski HJ: Myasthenia gravis: past, present, and future. J Clin Invest 2006;116:2843–2854.
10 Brambilla D, Mancuso C, Scuderi MR, Bosco P, Cantarella G, Lempereur L, Di Benedetto G, Pezzino S, Bernardini R: The role of antioxidant supplement in immune system, neoplastic, and neurodegenerative disorders: a point of view for an assessment of the risk/benefit profile. Nutr J 2008;7:29.
11 Venkatesham A, Sharath Babu P, Vidya Sagar J, Krishna DR: Effect of reactive oxygen species on cholinergic receptor function. J India Pharmacol 2005;6:366–370.
12 Poser CM, Paty DW, Scheinberg L, McDonald WI, Davis FA, Ebers GC, Johnson KP, Sibley WA, Silberberg DH, Tourtellotte WW: New diagnostic criteria for multiple sclerosis: guidelines for research protocols. Ann Neurol 1983;13:227–231.
13 McDonald WI, Compston A, Edan G, Goodkin D, Hartung HP, Lublin FD, McFarland HF, Paty DW, Polman CH, Reingold SC, Sandberg-Wollheim M, Sibley W, Thompson A, van den Noort S, Weinshenker BY, Wolinsky JS: Recommended diagnostic criteria for multiple sclerosis: guidelines from the international panel on the diagnosis of multiple sclerosis. Ann Neurol 2001;50:121–127.
14 Li L, Shou Y, Borowitz JL, Isom GE: Reactive oxygen species mediate pyridostigmine-Induced neuronal apoptosis: involvement of muscarinic and NMDA receptors. Toxicol Appl Pharmacol 2001;177:17–25.
15 Li L, Gunasekar PG, Borowitz JL, Isom GE: Muscarinic receptor-mediated pyridostigmine-induced neuronal apoptosis. Neurotoxicology 2000;21:541–552.
16 Halliwell B, Gutteridge JM, Cross CE: Free radicals, antioxidants and human disease: where are we now? J Lab Clin Med 1992;119: 598–620.
17 Drulović J, Dujmović I, Stojsavljević N, Mesaros S, Andjelković S, Miljković D, Perić V, Dragutinović G, Marinković J, Lević Z, Mostarica Stojković M: Uric acid levels in sera from patients with multiple sclerosis. J Neurol 2001;248:121–126.
18 Koprowski H, Spitsin SV, Hooper DC: Prospects for the treatment of multiple sclerosis by raising serum levels of uric acid, a scavenger of peroxynitrite. Ann Neurol 2001;49: 139.
19 Madhavan M, Wattigney WA, Srinivasan SR, Berenson GS: Serum bilirubin distribution and its relation to cardiovascular risk in children and young adults. Atherosclerosis 1997;131:107–113.
20 Ortenblad N, Madsen K, Djurhuus MS: Antioxidant status and lipid peroxidation after short-term maximal exercise in trained and untrained humans. Am J Physiol 1997; 272:R1258–R1263.
21 Chen J, Tu Y, Connolly EC, Ronnett GV: Heme oxygenase-2 protects against glutathione depletion-induced neuronal apoptosis mediated by bilirubin and cyclic GMP. Curr Neurovasc Res 2005;2:121–131.
22 Sedlak TW, Saleh M, Higginson DS, Paul BD, Juluri KR, Snyder SH: Bilirubin and glutathione have complementary antioxidant and cytoprotective roles. Proc Natl Acad Sci USA 2009;106:5171–5176.
23 Kirkby KA, Adin CA: Products of heme oxygenase and their potential therapeutic applications. Am J Physiol Renal Physiol 2006; 290:F563–F571.
24 Davison A, Tibbits G, Shi ZG, Moon J: Active oxygen in neuromuscular disorders. Mol Cell Biochem 1988;84:199–216.
25 Mancuso C, Bates TE, Butterfield DA, Calafato S, Cornelius C, De Lorenzo A, Dinkova Kostova AT, Calabrese V: Natural antioxidants in Alzheimer's disease. Expert Opin Investig Drugs 2007;16:1921–1931.
26 Whitton PS: Inflammation as a causative factor in the aetiology of Parkinson's disease. Br J Pharmacol 2007;150:963–976.
27 Drisko JA: The use of antioxidants in transmissible spongiform encephalopaties: a case report. J Am Coll Nutr 2002;21:22–25.

Downloaded by:
University of Pittsburgh
198.143.32.65 - 8/15/2015 5:42:01 AM

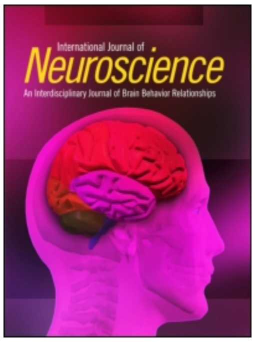

Taylor & Francis
Taylor & Francis Group

International Journal of Neuroscience

ISSN: 0020-7454 (Print) 1543-5245 (Online) Journal homepage: http://www.tandfonline.com/loi/ines20

Low antioxidant status of serum bilirubin, uric acid, albumin and creatinine in patients with myasthenia gravis

Dehao Yang, Zhongqian Su, Shengjie Wu, Yong Bi, Xiang Li, Jia Li, Kangliang Lou, Hongyu Zhang & Xu Zhang

To cite this article: Dehao Yang, Zhongqian Su, Shengjie Wu, Yong Bi, Xiang Li, Jia Li, Kangliang Lou, Hongyu Zhang & Xu Zhang (2015): Low antioxidant status of serum bilirubin, uric acid, albumin and creatinine in patients with myasthenia gravis, International Journal of Neuroscience, DOI: 10.3109/00207454.2015.1134526

To link to this article: http://dx.doi.org/10.3109/00207454.2015.1134526

Accepted author version posted online: 27 Dec 2015.

Submit your article to this journal

View related articles

View Crossmark data

Full Terms & Conditions of access and use can be found at
http://www.tandfonline.com/action/journalInformation?journalCode=ines20

Download by: [Simon Fraser University] **Date:** 29 December 2015, At: 18:56

Publisher: Taylor & Francis
Journal: *International Journal of Neuroscience*
DOI: http://dx.doi.org/10.3109/00207454.2015.1134526

Low antioxidant status of serum bilirubin, uric acid, albumin and creatinine in patients with myasthenia gravis

Dehao Yang[1] , Zhongqian Su[1] , Shengjie Wu[2] , Yong Bi[3] , Xiang Li[1] , Jia Li[1] , Kangliang Lou[4] , Hongyu Zhang[5 *] , Xu Zhang[1 *]

[1]Department of Neurology, the First Affiliated Hospital of Wenzhou Medical University, Wenzhou 325000, China

[2]Department of Cardiology, the Heart Center, the First Affiliated Hospital of Wenzhou Medical University, Wenzhou 325000, China.

[3]Department of Neurology, Zhejiang Provincial People's Hospital, Hangzhou 310014, China.

[4]School of the First Clinical Medical Sciences, Wenzhou Medical University, Wenzhou 325000, China

[5]School of Pharmacy, Key Laboratory of Biotechnology and Pharmaceutical Engineering, Wenzhou Medical University, Wenzhou 325000, China

[*]**Co-corresponding author:** Xu Zhang and Hongyu Zhang

Department of Neurology, the First Affiliated Hospital of Wenzhou Medical University，Wenzhou 325000, China; School of Pharmacy, Key Laboratory of Biotechnology and Pharmaceutical Engineering, Wenzhou Medical University,

ACCEPTED MANUSCRIPT

International Journal of Neuroscience

Wenzhou 325000, China

E-mail: drzhangxu@126.com; **Tel:** (86) 577-55579372 ; **Fax :** (86) 577-55579318.

Abstract

Objective: Oxidative stress and low antioxidant status play a major role in the pathogenesis of inflammatory and autoimmune diseases. Myasthenia gravis (MG) is an autoimmune condition targeting the neuromuscular junction, and its antioxidant status is still controversial. Our study aimed to investigate the correlation between the clinical characteristics of MG and the serum antioxidant status of bilirubin (Tbil, Dbil and Ibil), uric acid (UA), albumin, and creatinine.

Materials and Methods: We measured serum antioxidant molecule levels of bilirubin (Tbil, Dbil and Ibil), uric acid, albumin and creatinine in 380 individuals, including 166 MG and 214 healthy controls.

Results: We found that MG patients had significantly lower serum levels of bilirubin (Tbil, Dbil and Ibil), uric acid, albumin and creatinine than healthy controls, whether male or female. Moreover, it was also shown in our study that uric acid, albumin and creatinine levels in patients with MG were correlated with disease activity and classifications performed by the Myasthenia Gravis Foundation of America (MGFA).

Conclusion: Our findings demonstrated that serum levels of bilirubin (Tbil, Dbil and Ibil), uric acid, albumin and creatinine were reduced in patients with MG. This suggested an active oxidative process in MG patients who had low antioxidant status.

Keywords: Myasthenia gravis; Antioxidant ; Bilirubin ; Uric acid ; Albumin ; Creatinine

International Journal of Neuroscience

ACCEPTED MANUSCRIPT

Introduction

As chemically reactive molecules, reactive oxygen species (ROS) produced by enzymes and mitochondrial electron transport has performed important regulatory functions in cell signaling and homeostasis in normal physiological states [1]. However, excessive ROS levels, under a pathologic state of oxidative stress, irreversibly jeopardize cellular metabolism and cell structures, including lipids, carbohydrates, protein and DNAs [2]. To maintain the appropriate cellular redox balance, antioxidant defense systems should control excess ROS production by scavenging or decreasing ROS levels. Considered as a cytotoxic waste product and the end product of heme metabolism, bilirubin is an important potent antioxidant, which has anti-inflammatory and immunosuppressive properties [3]. Furthermore, bilirubin has much stronger antioxidant properties compared to a-tocopherol (vitamin E), catalase, and superoxide dismutase [4]. As the final product of the common pathway of purine metabolism, uric acid (UA) is a naturally occurring antioxidant, with metal-chelating properties [5]. Previous studies have showed that UA has the ability to scavenge nitrogen radicals and superoxide, thus helping to block the generation of the strong oxidant peroxynitrite [6]. Moreover, several studies confirmed that serum albumin has antioxidant properties and is a major known antioxidant role in extracellular fluids [7]. Creatinine, a metabolite of creatine phosphate and an energy store found in skeletal muscle [8], is one of components contributing to the total antioxidant assays [9]. Overall, bilirubin, uric acid, albumin and creatinine in the serum can have antioxidant effects and reduce global oxidative

International Journal of Neuroscience
ACCEPTED MANUSCRIPT

stress.

There is overwhelming evidence that oxidative stress is crucially involved in the development and progression of autoimmune disease [10], including multiple sclerosis [11], rheumatoid arthritis [12], systemic lupus erythematosus [13]. Myasthenia gravis (MG) is a neuromuscular autoimmune disease characterized by weakness and fatigability of skeletal muscles [14]. However, the relationship of antioxidant status and MG has not fully been elucidated. Therefore, the aim of our study was to investigate the correlation between MG and serum antioxidant levels of bilirubin, uric acid, albumin and creatinine performing a hospitalized-based study.

Patients and methods

Patients

The study population consisted of 380 individuals comprising 166 MG patients and 214 healthy controls (HC). The basic demographic and clinical characteristics of MG patients and healthy controls were summarized in table 1.

The diagnosis of MG was based on standard clinical criteria of characteristic weakness, fatigue, electrophysiology, neostigmine test and/or presence of autoantibody against skeletal muscle acetylcholine receptors[14] by a neurologist specializing in myasthenia gravis. The myasthenic condition was graded according to the Myasthenia Gravis Foundation of America(MGFA) Clinical Classification as follows: I = Any ocular muscle weakness; IIa = Predominantly affecting limb, axial muscles, or both; IIb = Predominantly affecting oropharyngeal, respiratory muscles,

International Journal of Neuroscience
ACCEPTED MANUSCRIPT

or both; IIIa = Predominantly affecting limb, axial muscles, or both; IIIb = Predominantly affecting oropharyngeal, respiratory muscles, or both; IVa = Predominantly affecting limb and/or axial muscles; IVb = Predominantly affecting oropharyngeal, respiratory muscles, or both; V = Defined by intubation, with or without mechanical ventilation, except when employed during routine postoperative management.

The exclusion criteria were as follows: liver disease or abnormal ranges of glutamate-pyruvate transaminase (GPT) and glutamic-oxaloacetic transaminase (GOT) concentrations; and subjects with history of diabetes, gout and renal failure.

Bilirubin, UA, Albumin and Creatinine Measurement

Venous blood was drawn by venipuncture in the morning after an overnight fasting to measure the concentration of serum bilirubin, UA, albumin and creatinine using a Clinical Analyzer Beckman Coulter AU5831 (Beckman Coulter, California, American). At the same time, concentrations of glutamate-pyruvate transaminase (GPT) (normal range: 9–50 μmol/L for male, 7–40 μmol/L for female), glutamic-oxaloacetic transaminase (GOT) (normal range: 15–40 μmol/L, 13–35 μmol/L for female), blood fasting sugar, blood urea nitrogen were also determined by an enzymatic method on the same analyzer.

Statistical Analysis

The statistical software Statistical Program for Social Sciences (version 20.0, SPSS Inc, Chicago, IL, USA) was used for all analyses. Results were expressed as the mean ± standard deviation. All statistical tests were two sided and p-values less than

International Journal of Neuroscience

ACCEPTED MANUSCRIPT

0.05 were considered statistically significant. The comparisons of bilirubin, uric acid, albumin and creatinine concentrations between patients with MG and HC subjects were performed using covariance analysis with age as covariant. The effect of age on serum bilirubin, uric acid, albumin and creatinine levels of different subgroups of patients with MG graded according to the MGFA Clinical Classification were analyzed by covariance analysis.

Results

In this study, we found serum levels of Tbil, Dbil and Ibil in patients with MG were significantly lower when compared with those of the HC group ($P < 0.001$, $P = 0.003$, $P < 0.001$ respectively) (Table 2, Figs 1A, 1B and 1C). Because previous evidence has shown the influence of the serum bilirubin levels by gender, our study further divided each group into two subgroups according to gender to eliminate the effect of gender (Table 2, Figs 1A, 1B and 1C). When compared with HC subjects, the MG patients had lower Tbil and Ibil, whether male or female. However, the serum Ibil levels of male patients with MG were not significantly lower compared to those in male HC group.

The mean (SD) serum uric acid in the MG group was 283.10 (± 90.35) μmol/L, whilst in the control group it was 333.83 (± 87.68) μmol/L. The difference was statistically significant ($P < 0.001$) (Table 2 and Fig. 1d). Serum UA level in male patients with MG was significantly lower than in male HC subgroup (328.39 ± 96.52 μmol/L vs. 391.61 ± 76.21 μmol/L, $P < 0.044$) (Table 2 and Fig. 1D). Similarly, serum

International Journal of Neuroscience

ACCEPTED MANUSCRIPT

UA level in MG was also significantly lower compared to that in female HC (248.40 ± 67.58 μmol/L VS. 284.10 ± 63.05 μmol/L, P <0.001) (Table 2 and Fig. 1D).

Serum levels of albumin were lower in MG than in HC group (P < 0.001) in this study (Table 2 and Fig. 1E). Furthermore, in male groups, we found that albumin values in patients with MG were also significantly lower than those in HC subgroup, consistent with the results when the female subgroup was investigated separately (Table 2 and Fig. 1E).

Patients with MG displayed a decrease in the serum creatinine levels compared to the HC group (P < 0.001, Table 2, Fig. 1F). There were significantly lower mean serum levels of creatinine in male patients with MG than those in the male HC group, while there were no statistic difference in this one between those with the female MG patients and the female HC group (Table 2, Fig. 1F).

Generally, we compared the serum bilirubin, uric acid, albumin and creatinine concentration in women vs. men in all groups. The mean serum levels of Tbil, Dbil, UA, albumin and creatinine were significantly lower in female group to male group, while there were no significant difference in serum Ibil values between those groups (8.99 ± 2.68 μmol/L vs. 9.87 ± 4.20 μmol/L, P = 0.064) (Table 2).

Moreover, to better elucidate the relation between serum bilirubin, uric acid, albumin and creatinine concentration and MG grades, patients with MG were divided into four subgroups according to the MGFA Clinical Classification: A subgroup = MGFA I; B subgroup = MGFA IIa and IIb; C subgroup = MGFA IIIa, IIIb and IVa; D subgroup = MGFA IVb and V (table 3). There were no significant differences of

International Journal of Neuroscience

ACCEPTED MANUSCRIPT

serum bilirubin levels, whether total or fractionated, between groups of disease severity. However, we found that the relative decrease in the mean serum UA, albumin and creatinine level of the patients with MG correlated with the degree of disease progression, as expressed by MGFA clinical classification (Table 3, Figs 2, 3 and 4). In addition, patients with MG were further subdivided into two groups: group 1 of 63 patients with thymoma presented on MRI or CT and group 2 of 103 patients without thymoma. Nevertheless, there was no statistical difference in serum Tbil, Dbil, Ibil, UA, albumin and creatinine level between group 1 and f group 2 ($p = 0.568$, $p = 0.904$, $p = 0.444$, $p = 0.297$, $p = 0.547$, $p = 0.789$ respectively) (table 4).

Discussion

The present study demonstrated that serum Tbil, Dbil, Ibil, UA, and albumin levels are decreased in MG patients. Furthermore, the same results were also obtained when the male and female groups were investigated separately. To the best of our knowledge, this is the first clinical study to systematically and comprehensively evaluate the association between MG and low antioxidant status of bilirubin, UA, albumin, and creatinine.

Autoimmune myasthenia gravis (MG), caused by an autoimmune attack against components of the neuromuscular junction (NMJ) on the postsynaptic membrane of the striated skeletal muscles, is a neuromuscular disorder characterized by a defective transmission of nerve impulses to muscles, which is mostly mediated by antibodies against the acetylcholine receptor (AChR) [15]. Because of the evidence implicating

International Journal of Neuroscience

ACCEPTED MANUSCRIPT

oxidative stress in inflammatory and autoimmune-mediated tissue destruction [10], including multiple sclerosis [11], rheumatoid arthritis [12], systemic lupus erythematosus [13], it has been postulated that reduced antioxidant reserve is possibly an early pathogenic mechanism in MG, as MG is one of autoimmune diseases.

ROS plays a role in injuring the body's cells and tissues through multiple pathways, including direct damage to the biological structures, such as cell membrane, genetic material, and enzymes, and indirectly stimulating the expression of gene associated with apoptosis. Recently, Venkatesham A and colleagues [16] found confirmatory evidence that reactive oxygen species may contribute to damage to the AChR. Furthermore, Arjun Krishnaswamy concluded that the effects of ROS on nAChR function are due to the highly conserved Cys residues in the receptors [17]. Indeed, antioxidant status of bilirubin, UA, albumin and creatinine in MG patients was decreased in this study.

We reasoned that the result may be explained by the antioxidant defense systems of these patients scavenging excessive ROS with antioxidant status of bilirubin, UA, albumin, and creatinine decreasing. Bilirubin is relevant to the prevention of oxidant-mediated cell death [18] and is a potential free-radical scavenger, possessing physiologic properties akin to the anti-oxidant effects of Vitamin C and Vitamin E[4]. Some studies have demonstrated bilirubin to protect against oxidative stress in many diseases, including diabetic nephropathy, cancer and diabetic nephropathy[19, 20]. Since bilirubin is an endogenous anti-oxidant against ROS, the lower bilirubin concentrations in MG patients might result from an overconsumption of bilirubin by

International Journal of Neuroscience
ACCEPTED MANUSCRIPT

ROS in patients with MG patients. Some studies have indicated that UA has been successfully used to prevent and delay the development and progression of symptoms in the animal model of MS [21] and as the precursor, inosine, may also reduce MS relapse rates [22]. Creatinine, a metabolite of creatine phosphate, primarily depended on skeletal muscle mass in blood, has been demonstrated to be an efficient scavenger of free radicals[9]. In our study patients with MG have lower creatinine. We found that creatinine levels were significantly lower in MG patients than healthy controls and were correlated with disease activity and classifications performed by the Myasthenia Gravis Foundation of America (MGFA). We believed that the lower serum creatinine levels might be due to the consumption by radical interaction or due to lower metabolism, because patients with MG have loss of muscle due to inactivity that occurs with the disease progression. However, further study needs to investigate the mechanism .

In the present study, the serum levels of bilirubin, uric acid, albumin and creatinine in patients with MG were significantly lower in women than in men. However, the results suggest, as shown in MS [23], that the female gender contributes significantly to the difference between MG patients and control subjects, which may be related since the incidence of MG is higher in women than in men.

To better clarify the underlying mechanisms of antioxidant status in MG, patients were divided into four subgroups according to the MGFA Clinical Classification. We found that the relative decrease in the mean serum UA, albumin and creatinine level of the patients with MG correlated with the degree of disease progression, as

International Journal of Neuroscience

ACCEPTED MANUSCRIPT

expressed by MGFA Clinical Classification. Although Peng et al. [24] described that there was no correlation between serum UA or bilirubin and disease grade according to the modified Osserman classification, MGFA typing, a new clinical classification and scoring system put forward by Myasthenia Gravis Foundation of America, was more accurate and objective than Osserman typing [25, 26]. Our data suggested that low antioxidant status in MG patients correlated with disability as assessed by MGFA Clinical Classification. Nevertheless, further study of more evidences is necessary to confirm our findings. However, in the present study, there was no correlation between serum bilirubin, UA, albumin or creatinine, and thymoma presented on MRI or CT.

In summary, the present study showed that patients with MG had low serum bilirubin, uric acid, albumin, and creatinine concentrations. Nevertheless, it was also uncertain whether low antioxidant status of serum bilirubin, uric acid, albumin and creatinine levels was a cause or a consequence of MG activity. Indeed, MG patients were unable to protect neuromuscular junction against direct oxidative damage and inflammatory with low antioxidant status.

Conclusion

Our data supported the hypothesis that oxidative stress was implicated in the pathogenic mechanism of MG. As a replacement therapy to patients with low antioxidant status, administration of bilirubin, uric acid, albumin and creatinine or theirs precursors might be beneficial to the patients with MG.

International Journal of Neuroscience

ACCEPTED MANUSCRIPT

International Journal of Neuroscience

Acknowledgement

The present study was supported by the Natural Science Foundation of Zhejiang Province (No. LY13H090010).

Disclosure of conflict of interest

The authors declare no financial or other conflict of interests.

ACCEPTED MANUSCRIPT

References

[1] Lassegue B, San Martin A, Griendling KK. Biochemistry, physiology, and pathophysiology of NADPH oxidases in the cardiovascular system. Circ Res 2012;110:1364-90.

[2] Pauwels EK, Erba P, Kostkiewicz M. Antioxidants: a tale of two stories. Drug News Perspect 2007;20:579-85.

[3] Jangi S, Otterbein L, Robson S. The molecular basis for the immunomodulatory activities of unconjugated bilirubin. Int J Biochem Cell Biol 2013;45:2843-51.

[4] Stocker R, Yamamoto Y, McDonagh AF, Glazer AN, Ames BN. Bilirubin is an antioxidant of possible physiological importance. Science 1987;235:1043-6.

[5] Davies KJ, Sevanian A, Muakkassah-Kelly SF, Hochstein P. Uric acid-iron ion complexes. A new aspect of the antioxidant functions of uric acid. Biochem J 1986;235:747-54.

[6] Whiteman M, Ketsawatsakul U, Halliwell B. A reassessment of the peroxynitrite scavenging activity of uric acid. Ann N Y Acad Sci 2002;962:242-59.

[7] Roche M, Rondeau P, Singh NR, Tarnus E, Bourdon E. The antioxidant properties of serum albumin. FEBS Lett 2008;582:1783-7.

[8] Nyasavajjala SM, Phillips BE, Lund JN, Williams JP. Creatinine and myoglobin are poor predictors of anaerobic threshold in colorectal cancer and health. J Cachexia Sarcopenia Muscle 2015;6:125-31.

[9] Jansen EH, Beekhof PK, Cremers JW, Viezeliene D, Muzakova V, Skalicky J. Long-term stability of parameters of antioxidant status in human serum. Free Radic Res 2013;47:535-40.

[10] Brambilla D, Mancuso C, Scuderi MR, et al. The role of antioxidant supplement in immune system, neoplastic, and neurodegenerative disorders: a point of view for an assessment of the risk/benefit profile. Nutr J 2008;7:29.

[11] Wang P, Xie K, Wang C, Bi J. Oxidative stress induced by lipid peroxidation is related with inflammation of demyelination and neurodegeneration in multiple sclerosis. Eur Neurol 2014;72:249-54.

[12] Ishibashi T. Molecular hydrogen: new antioxidant and anti-inflammatory therapy for rheumatoid arthritis and related diseases. Curr Pharm Des 2013;19:6375-81.

[13] Perl A. Oxidative stress in the pathology and treatment of systemic lupus erythematosus. Nat Rev Rheumatol 2013;9:674-86.

[14] Drachman DB. Myasthenia gravis. N Engl J Med 1994;330:1797-810.

[15] Berrih-Aknin S, Frenkian-Cuvelier M, Eymard B. Diagnostic and clinical classification of autoimmune myasthenia gravis. J Autoimmun 2014;48-49:143-8.

[16] Venkatesham A, Sharath Babu P, Vidya Sagar J, Krishna D. Effect of reactive oxygen species on cholinergic receptor function. J India Pharmacol 2005;6:366–370.

ACCEPTED MANUSCRIPT

[17] Krishnaswamy A, Cooper E. Reactive oxygen species inactivate neuronal nicotinic acetylcholine receptors through a highly conserved cysteine near the intracellular mouth of the channel: implications for diseases that involve oxidative stress. J Physiol 2012;590:39-47.

[18] Kushida T, LiVolti G, Goodman AI, Abraham NG. TNF-alpha-mediated cell death is attenuated by retrovirus delivery of human heme oxygenase-1 gene into human microvessel endothelial cells. Transplant Proc 2002;34:2973-8.

[19] Riphagen IJ, Deetman PE, Bakker SJ, et al. Bilirubin and progression of nephropathy in type 2 diabetes: a post hoc analysis of RENAAL with independent replication in IDNT. Diabetes 2014;63:2845-53.

[20] Peng F, Deng X, Yu Y, et al. Serum bilirubin concentrations and multiple sclerosis. J Clin Neurosci 2011;18:1355-9.

[21] Hooper DC, Scott GS, Zborek A, et al. Uric acid, a peroxynitrite scavenger, inhibits CNS inflammation, blood-CNS barrier permeability changes, and tissue damage in a mouse model of multiple sclerosis. FASEB J 2000;14:691-8.

[22] Markowitz CE, Spitsin S, Zimmerman V, et al. The treatment of multiple sclerosis with inosine. J Altern Complement Med 2009;15:619-25.

[23] Peng F, Zhang B, Zhong X, et al. Serum uric acid levels of patients with multiple sclerosis and other neurological diseases. Mult Scler 2008;14:188-96.

[24] Peng F, Deng X, Zhou Z, et al. Antioxidant status of bilirubin and uric acid in patients with myasthenia gravis. Neuroimmunomodulation 2012;19:43-9.

[25] Jaretzki A, 3rd, Barohn RJ, Ernstoff RM, et al. Myasthenia gravis: recommendations for clinical research standards. Task Force of the Medical Scientific Advisory Board of the Myasthenia Gravis Foundation of America. Ann Thorac Surg 2000;70:327-34.

[26] Chen Z, Luo H, Peng Y, et al. Comparative clinical features and immune responses after extended thymectomy for myasthenia gravis in patients with atrophic versus hyperplastic thymus. Ann Thorac Surg 2011;91:212-8.

International Journal of Neuroscience

ACCEPTED MANUSCRIPT

FIGURE LEGENDS

Fig. 1 Serum bilirubin, UA, albumin and creatinine levels in MG patients and healthy control group: (A) total bilirubin; (B) direct bilirubin; (C) indirect bilirubin; (D) UA; (E) albumin; (F) creatinine.

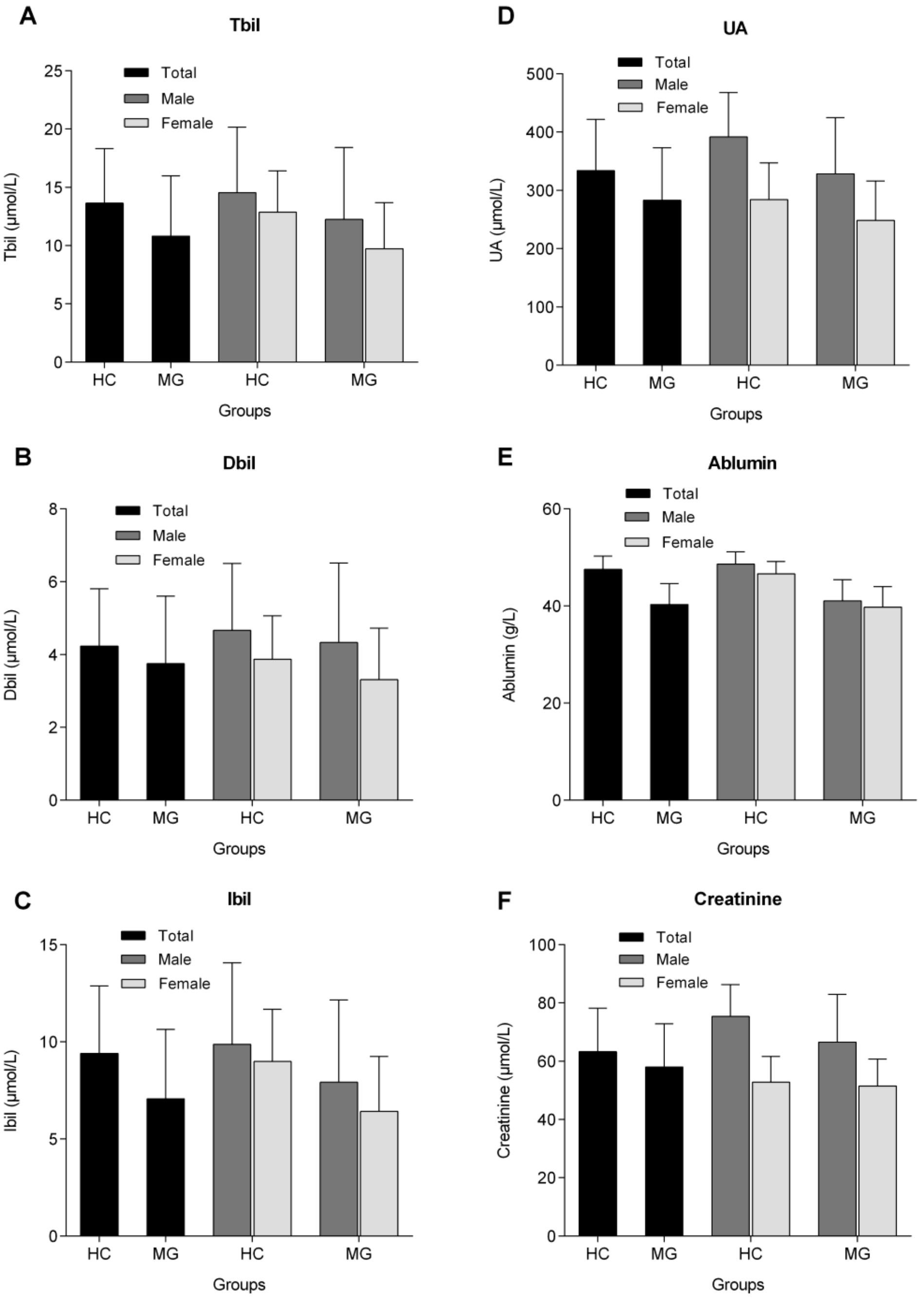

Fig. 2 Serum uric acid levels in four subgroups according to the MGFA Clinical Classification (*P<0.05, **P<0.01, ***P<0.001).

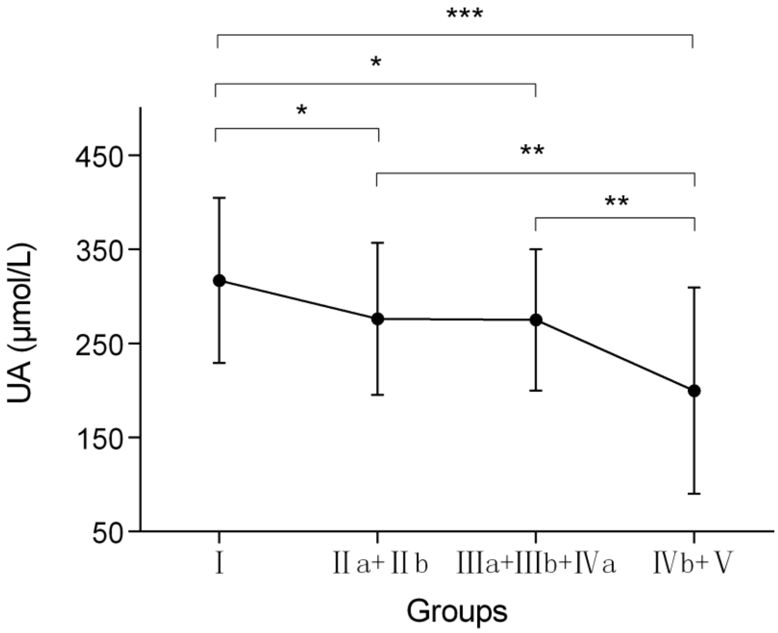

ACCEPTED MANUSCRIPT

Fig. 3 Serum albumin levels in four subgroups according to the MGFA Clinical Classification (*P<0.05, **P<0.01, ***P<0.001).

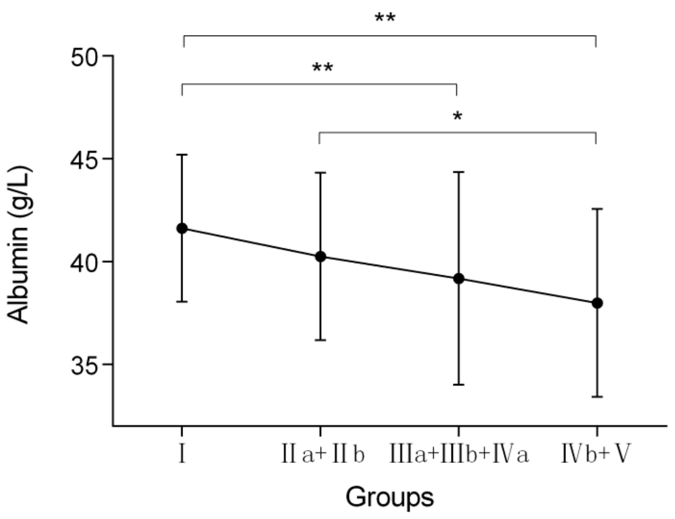

Fig. 4 Serum creatinine levels in four subgroups according to the MGFA Clinical Classification (*P<0.05, **P<0.01, ***P<0.001).

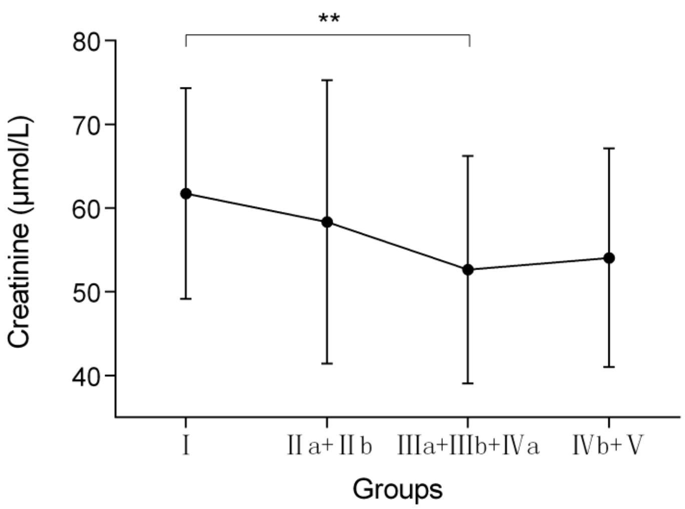

International Journal of Neuroscience

ACCEPTED MANUSCRIPT

TABLES

Subjects	All patients	Male	Female	Duration of disease (months)[a]	Ages (years)[b]
MG	166	72	94	6.0 (2.0 – 36.0)	42.59 ± 15.66
HC	214	99	115	-	43.68 ± 14.15

a: Median and 25th–75th when duration of disease;
b: Mean ± SD when ages

Table 1 Demographic and clinical characteristics of MG patients and healthy control group

Table 2 Serum bilirubin, UA, albumin and creatinine levels in MG patients and healthy control group (Mean ± SD)

Patients	Total	Male	Female	P^1	P^2	P^3	P^4
Tbil (μmol/L)							
MG	10.82±5.17	12.25±6.17	9.72±3.96				0.002
HC	13.66±4.69	14.53±5.62	12.86±3.55	<0.001	0.013	<0.001	0.012
Dbil (μmol/L)							
MG	3.75±1.85	4.33±2.18	3.31±1.41				<0.001
HC	4.23±1.57	4.66±1.84	3.87±1.19	0.003	0.895	<0.001	0.001
Ibil (μmol/L)							
MG	7.06±3.58	7.92±4.24	6.41±2.83				0.010
HC	9.40±3.48	9.87±4.20	8.99±2.68	<0.001	0.001	<0.001	0.064
UA (μmol/L)							
MG	283.10±90.35	328.39±96.52	248.40±67.58				<0.001
HC	333.83±87.68	391.61±76.21	284.10±63.05	<0.001	0.044	<0.001	<0.001
Albumin (g/L)							
MG	40.31±4.31	41.07±4.33	39.73±4.22				0.002
HC	47.54±2.73	48.60±2.57	46.63±2.53	<0.001	<0.001	<0.001	<0.001
Creatinine (μmol/L)							
MG	57.99±14.84	66.50±16.46	51.48±9.22				<0.001
HC	63.23±14.95	75.34±10.91	52.80±8.84	0.001	<0.001	0.559	<0.001

P^1 = MG versus HC; P^2 = male patients with MG versus male HC; P^3 = female patients with MG versus female HC; P^4 = male versus female in each group

International Journal of Neuroscience

ACCEPTED MANUSCRIPT

Table 3 Serum bilirubin, UA, albumin and creatinine levels in patients with MG according to the MGFA Clinical Classification

Subjects	Ⅰ	Ⅱa+Ⅱb	Ⅲa+Ⅲb+Ⅳa	Ⅳb+Ⅴ	P
Patients	57	61	33	15	
Tbil (μmol/L)	11.16±6.24	10.49±3.68	11.39±6.27	9.60±2.87	0.628
Dbil (μmol/L)	3.84±2.10	3.69±1.41	3.88±2.36	3.40±1.06	0.833
Ibil (μmol/L)	7.32±4.37	6.80±2.60	7.52±4.14	6.20±2.11	0.550
UA (μmol/L)	317.04±87.74	276.21±80.78	275.06±75.23	199.80±109.70	<0.001
Albumin (g/L)	41.63±3.57	40.26±4.07	39.19±5.17	37.99±4.57	0.005
Creatinine (μmol/L)	61.75±12.59	58.34±16.92	52.64±13.60	54.07±13.05	0.029

Table 4 Serum bilirubin, UA, albumin and creatinine levels in MG patients with thymoma

Subjects	Non-thymoma	Thymoma	P
Patients	103	63	
Tbil (μmol/L)	10.64±4.83	11.11±5.72	0.568
Dbil (μmol/L)	3.74±1.75	3.78±2.00	0.904
Ibil (μmol/L)	6.90±3.33	7.33±3.96	0.444
UA (μmol/L)	277.31±87.59	292.56±94.65	0.297
Albumin (g/L)	40.46±4.18	40.07±4.53	0.547
Creatinine (μmol/L)	57.80±15.29	58.32±14.19	0.789

International Journal of Neuroscience

ACCEPTED MANUSCRIPT

Serum uric acid levels in patients with myasthenia gravis are inversely correlated with disability

Dehao Yang[a,*], Yiyun Weng[a,*], Haihua Lin[b], Feiyan Xie[c], Fang Yin[e], Kangliang Lou[e], Xuan Zhou[e], Yixiang Han[d], Xiang Li[a] and Xu Zhang[a]

Uric acid (UA), the final product of purine metabolism, has been reported to be reduced in patients with various neurological disorders and is considered to be a possible indicator for monitoring the disability and progression of multiple sclerosis. However, it remains unclear whether there is a close relationship between UA and myasthenia gravis (MG), or whether UA is primarily deficient or secondarily reduced because of its peroxynitrite scavenging activity. We investigated the correlation between serum UA levels and the clinical characteristics of MG. We assessed 338 serum UA levels obtained in 135 patients with MG, 47 patients with multiple sclerosis, and 156 healthy controls. In addition, we compared serum UA levels when MG patients were stratified according to disease activity and classifications performed by the Myasthenia Gravis Foundation of America, age of onset, duration, and thymus histology (by means of MRI or computed tomography). MG patients had significantly lower serum UA levels than the controls ($P < 0.001$). Moreover, UA levels in patients with MG were inversely correlated with disease activity and disease progression ($P = 0.013$). However, UA levels did not correlate significantly with disease duration, age of onset, and thymus histology. Our findings suggest that serum level of UA was reduced in patients with MG and serum UA might be considered a surrogate biomarker of MG disability and progression. ***NeuroReport*** **27:301–305 Copyright © 2016 Wolters Kluwer Health, Inc. All rights reserved.**

NeuroReport 2016, **27**:301–305

Keywords: disability, myasthenia gravis, uric acid

Departments of [a]Neurology, [b]Gastroenterology, [c]Surgical Oncology, [d]Laboratory of Internal Medicine, the First Affiliated Hospital of Wenzhou Medical University and [e]School of the First Clinical Medical Sciences, Wenzhou Medical University, Wenzhou, China

Correspondence to Xu Zhang, MD, Department of Neurology, the First Affiliated Hospital of Wenzhou Medical University, Wenzhou 325000, China
Tel: + 86 577 555 79372; fax: + 86 577 555 79318; e-mail: drzhangxu@126.com

*Dehao Yang and Yiyun Weng are co-first authors.

Received 22 December 2015 **accepted** 11 January 2016

Introduction

Myasthenia gravis (MG), caused by autoantibodies against the acetylcholine receptor (AChR) on the post-synaptic membrane at the neuromuscular junction, is an acquired autoimmune disease characterized by a defective transmission of nerve impulses to muscles [1]. Accumulating data have implicated oxidative stress in the immunopathogenesis of neuromuscular diseases [2,3].

As the final product of the common pathway of purine metabolism, uric acid (UA) is a naturally occurring antioxidant, with metal-chelating properties [4]. Previous studies have reported that UA can scavenge nitrogen radicals and superoxide, thus helping to block the generation of the strong oxidant peroxynitrite [5]. Peroxynitrite exerts toxic effects and irreversibly jeopardizes cellular metabolism and cell structures, including lipids, carbohydrates, protein, and DNAs [6]. Several studies have identified a therapeutic role of UA in experimental allergic encephalomyelitis and a beneficial function for increasing serum UA levels in multiple sclerosis (MS) patients [7,8]. Furthermore, UA might be a surrogate marker for monitoring MS activity [9].

Therefore, the aim of this study was to investigate whether the serum UA levels were decreased in MG patients and whether the decrease was associated with disease disability and progression.

Patients and methods

Serum samples were collected from 338 individuals: 135 patients with MG, 47 patients with MS, and 156 healthy controls (CTL). Venous blood was drawn from an antecubital vein in the morning after an overnight fast to measure the concentration of serum UA using a Clinical Analyzer Beckman Coulter AU5831 (Beckman Coulter, Brea, California, USA). In our hospital, the normal range of serum UA values is 208–428 μM for men and 155–357 μM for women. Simultaneously, concentrations of glutamate–pyruvate transaminase (normal range: 9–50 μM for men, 7–40 μM for women), glutamic–oxaloacetic transaminase (normal range: 15–40 μM, 13–35 μM for women), blood fasting sugar, and blood urea nitrogen were also measured using an enzymatic method on the same analyzer.

This is an open-access article distributed under the terms of the Creative Commons Attribution-Non Commercial-No Derivatives License 4.0 (CCBY-NC-ND), where it is permissible to download and share the work provided it is properly cited. The work cannot be changed in any way or used commercially.

0959-4965 Copyright © 2016 Wolters Kluwer Health, Inc. All rights reserved.

DOI: 10.1097/WNR.0000000000000535

Table 1 MGFA clinical classification

Class I	Any ocular muscle weakness<br>May have weakness of eye closure<br>All other muscle strength is normal
Class II	Mild weakness affecting other than ocular muscles<br>May also have ocular muscle weakness of any severity
IIa	Predominantly affecting limb, axial muscles, or both<br>May also have lesser involvement of oropharyngeal muscles
IIb	Predominantly affecting oropharyngeal, respiratory muscles, or both<br>May also have lesser or equal involvement of limb, axial muscles, or both
Class III	Moderate weakness affecting other than ocular muscles<br>May also have ocular muscle weakness of any severity
IIIa	Predominantly affecting limb, axial muscles, or both<br>May also have lesser involvement of oropharyngeal muscles
IIIb	Predominantly affecting oropharyngeal, respiratory muscles, or both<br>May also have lesser or equal involvement of limb, axial muscles, or both
Class IV	Severe weakness affecting other than ocular muscles<br>May also have ocular muscle weakness of any severity
IVa	Predominantly affecting limb and/or axial muscles<br>May also have lesser involvement of oropharyngeal muscles
IVb	Predominantly affecting oropharyngeal, respiratory muscles, or both<br>May also have lesser or equal involvement of limb, axial muscles, or both
Class V	Defined by intubation, with or without mechanical ventilation, except when used during routine postoperative management. The use of a feeding tube without intubation places the patient in class IVb

MG, myasthenia gravis; MGFA, Myasthenia Gravis Foundation of America.

All patients in hospital care had definite MG according to the standard clinical criteria of Drachman [10]. The severity of the disease (Table 1) was estimated according to the Myasthenia Gravis Foundation of America (MGFA) clinical classification at the time of blood sampling [11]. The exclusion criteria were liver disease or abnormal ranges of glutamate–pyruvate transaminase and glutamic–oxaloacetic transaminase concentrations, as well as patients with a history of gout, diabetes, and renal failure.

The statistical software statistical program for social sciences (version 20.0; SPSS Inc., Chicago, Illinois, USA) was used for all analyses. Data in this study were expressed as the mean ± SD values. All statistical tests were two sided and *P*-values less than 0.05 were considered statistically significant. The comparisons of UA concentrations among patients with MG, patients with MS, and control participants were performed using covariance analysis with age as a covariant. Because previous evidence has shown the effect on the serum UA concentrations by sex, patients in each group were divided into two subgroups on the basis of their sex and analyzed with age as a covariant. The effect of age on serum UA levels of different subgroups of patients with MG graded according to the MGFA Clinical Classification was analyzed by covariance analysis. The levels of UA were compared when patients were stratified according to age of onset, disease duration, MGFA clinical classification, and thymus histology. Significance was calculated using covariance analysis with age as the covariant.

Results

The basic demographic and clinical characteristics of patients with MG, patients with MS, and healthy controls are shown in Table 1. In total, 338 individuals provided samples. These included 135 MG patients (57 men, 78 women), 47 MS patients (13 men, 34 women), and 156 controls (CTL, 69 men, 87 women), who did not differ significantly in age (MG 41.6 ± 15.8, MS 41.5 ± 10.5, CTL 42.9 ± 14.3; $P = 0.684$).

In the present study, the mean serum UA level in all participants was 304 ± 92 μM. The serum UA level was significantly lower in the MG patients than in the healthy controls (283 ± 90 vs. 335 ± 84 μM; $P < 0.001$). However, no difference was found between MG and MS (283 ± 90 vs. 257 ± 85 μM; $P = 0.072$) (Fig. 1). Previous evidence has shown that serum UA levels are significantly lower in women than in men [12]. Accordingly, we divided each cohort into men and women to eliminate the possibility that the differences that we observed were simply because of the different numbers of men and women in the disease groups. Interestingly, in all groups, the serum UA level in women was significantly lower than that in men ($P < 0.001$) (Table 2 and Fig. 2). Compared with CTL patients, MG patients had lower serum UA level, whether male or female. However, there was no statistical difference in UA levels between patients with MG and MS, whether in male or in female subgroups ($P = 0.641$ and 0.204, respectively) (Table 2 and Fig. 2).

The correlations between UA levels in MG patients, with their disease duration, age of onset, MGFA clinical classification, and thymus histology, are presented in Table 3. In MG, the UA level was not lower in patients with late-onset MG (age at onset ≥ 50 years) than early-

Fig. 1

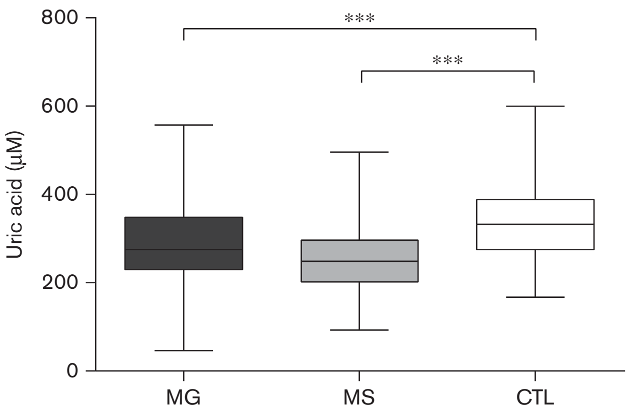

Serum UA levels in patients with MG, MS, and CTL. UA levels measured in MG patients were significantly lower than those in CTL, but were not different from those measured in MS patients. CTL, healthy control group; MG, myasthenia gravis; MS, multiple sclerosis; UA, uric acid. ***$P < 0.001$.

Table 2 Serum UA levels in male and female patients and healthy controls

Patients	Male (μM)	Female (μM)	P_1	P_2	P_3
MG	333 ± 95	247 ± 67	< 0.001		
MS	324 ± 75	231 ± 74	< 0.001	0.641	0.204
CTL	395 ± 74	288 ± 57	< 0.001	< 0.001	< 0.001

CTL, healthy control group; MG, myasthenia gravis; MS, multiple sclerosis; UA, uric acid.
P_1 = male versus female in each group.
P_2 = male patients with multiple sclerosis or the healthy control group versus male patients with myasthenia gravis.
P_3 = female patients with multiple sclerosis or the healthy control group versus female patients with myasthenia gravis.

Fig. 2

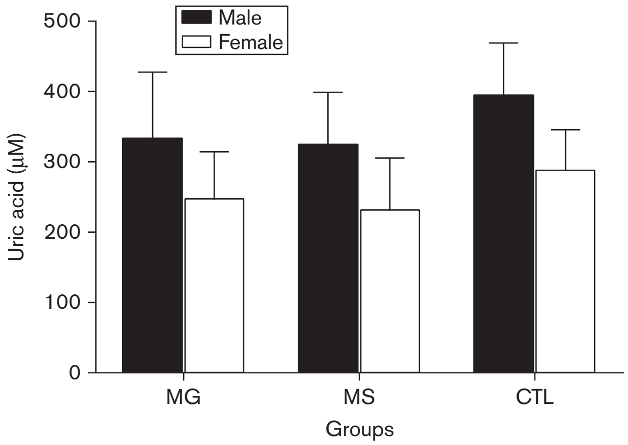

Serum UA levels in male and female patients with MG, MS, and CTL. UA levels measured in male or female MG patients were significantly lower than those in CTL, but were not different from those measured in MS patients. CTL, healthy control group; MG, myasthenia gravis; MS, multiple sclerosis; UA, uric acid.

Table 3 Serum UA levels in patients with MG

Variables	Mean	SD	Range	P
Age of onset (years)				0.664
< 50 (*n* = 92)	280	94	46–500	
≥ 50 (*n* = 43)	291	82	118–558	
Duration of disease (years)				0.261
≤ 1 (*n* = 101)	290	87	46–558	
> 1 (*n* = 34)	264	97	87–469	
MGFA				0.013
I (*n* = 43)	321	88	151–558	
II (*n* = 48)	282	80	118–436	
III (*n* = 25)	275	76	153–500	
IV (*n* = 8)	240	41	193–319	
V (*n* = 11)	197	128	46–469	
MGFA				0.707
IIa, IIIa, IVa (*n* = 34)	285	74	162–500	
IIb, IIIb, IVb (*n* = 47)	269	77	118–426	
Thymus histology				0.867
Nonthymoma (*n* = 83)	284	90	118–558	
Thymoma (*n* = 52)	282	92	46–500	

Correlation of serum UA levels with age of onset, disease duration, MGFA clinical classification, and thymus histology.
MG, myasthenia gravis; MGFA, Myasthenia Gravis Foundation of America; UA, uric acid.

Fig. 3

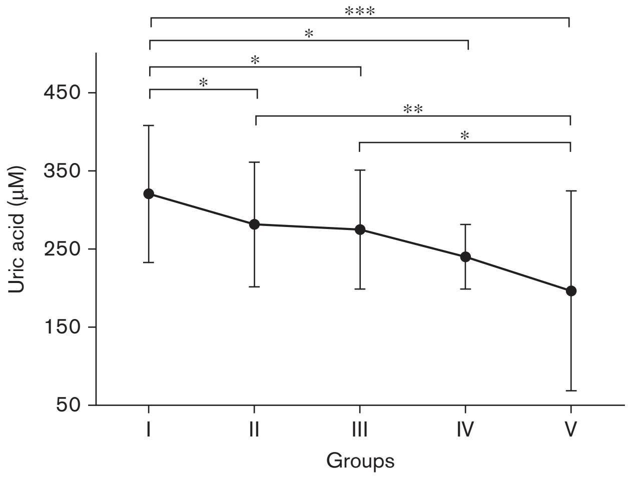

Serum UA levels in five subgroups according to the MGFA clinical classification. MGFA, Myasthenia Gravis Foundation of America; UA, uric acid. *$P < 0.05$, **$P < 0.01$, ***$P < 0.001$.

onset MG (age at onset < 50 years) ($P = 0.664$) (Table 3). There was no significant difference ($P = 0.261$) in UA levels between patients with longer disease duration (>1 year) and those with short disease duration (≤ 1 year), although the former was somewhat lower than the latter (Table 3). The serum UA level was not lower in patients with thymoma shown on MRI or computed tomography than in patients without thymoma ($P = 0.867$). However, we found that the relative decrease in the mean serum UA level of the patients with MG correlated inversely with the degree of disease progression, as expressed by the MGFA clinical classification (Table 3 and Fig. 3). On grouping MGFA IIa, IIIa, and IVa categories (absence of bulbar involvement) and comparing them with IIb, IIIb, and IVb categories (presence of bulbar involvement), no significant difference was observed between bulbar independent and involved bulbar MG patient groups (Table 3).

Discussion

MG is a severe autoimmune disease characterized by its tendency to selectively affect AChR of the postsynaptic membrane, which is associated with B and T cell activation [13]. The role of oxidative stress in MG has not been fully studied. Reactive oxygen species play a role in injuring the body's cells and tissues through multiple pathways, including direct damage to the biological structures, such as cell membrane, genetic material, and enzymes, and indirectly stimulating the expression of genes associated with apoptosis. Furthermore, accumulating evidence has shown that oxidative stress contributes toward the pathogenesis in inflammatory and autoimmune-mediated tissue destruction [14]. Skeletal muscle is strongly dependent on oxidative metabolism,

and corresponding antioxidant protection mechanisms are present. Venkatesham *et al.* [3] have shown that reactive oxygen species might contribute toward damage to the AChR. Moreover, Krishnaswamy and Cooper [15] has suggested that the highly conserved cysteine residues in nicotinic AChR are the major targets of reactive oxygen species producing receptor dysfunction. Highly conserved cysteine residue, a component of several nicotinic AChRs on neurons, locates near the intracellular mouth of the receptor pore and enables ganglionic transmission and sympathetic reflexes to function normally.

UA, a naturally occurring product of purine metabolism, is an important natural antioxidant that could scavenge superoxides and reduce oxidative stress [16]. Numerous studies have shown that there is a reduced serum UA level in inflammatory and autoimmune diseases [17–19]. Previous studies in MS showed that UA may be a surrogate marker of MS activity and it is one of the antioxidants evaluated for their effect on MS disease progression [20]. Moreover, UA treatment has been proven to prevent inflammation and destruction of central nervous tissue and ameliorate established disease in the animal models of MS [21].

In this study, MG patients presented significantly lower serum UA than healthy controls. However, the difference in serum UA between MG patients and MS patients was not significant. Furthermore, similar results were also observed when the female and male cohorts were investigated separately. A relatively high proportion of patients with MG, in our hospital, had serum UA levels below the lower limit of the normal range, which was similar to the previous report [22]. However, the correlation between the serum UA and the clinical characteristics of MG was not investigated in the previous study. To better clarify the underlying mechanisms of antioxidant status in MG, patients were divided into five subgroups according to the MGFA clinical classification. Patients with clinically active disease had significantly lower UA levels than those with clinically inactive disease. However, comparison of UA levels in patients stratified according to MG disease duration, age of onset, and thymus histology showed no significant difference. Nevertheless, it was also uncertain whether a low concentration of UA was a cause or a consequence of MG progression and activity. Further studies are necessary to clarify the role of UA and its underlying mechanisms in patients with MG. Nonetheless, patients with MG were unable to protect neuromuscular junction against oxidative stress with low antioxidant status.

Conclusion

Our findings of a significant inverse correlation between serum UA level and disease activity and disability, as assessed by the MGFA clinical classification, indicate that serum UA might serve as a possible biomarker of disease disability in MG. To our knowledge, the present study is the first description of an inverse correlation of serum UA level with MG disability as assessed by the MGFA clinical classification. Confirmation of these conclusions with a much larger sample will be an important next step.

Acknowledgements

The present study was supported by the Natural Science Foundation of Zhejiang Province (No. LY13H090010) and the Wenzhou Municipal Sci-Tech Bureau Program (No. Y20140278).

Conflicts of interest

There are no conflicts of interest.

References

1 Meriggioli MN, Sanders DB. Autoimmune myasthenia gravis: emerging clinical and biological heterogeneity. *Lancet Neurol* 2009; **8**:475–490.
2 Stuerenburg HJ. The roles of carnosine in aging of skeletal muscle and in neuromuscular diseases. *Biochemistry (Mosc)* 2000; **65**: 862–865.
3 Venkatesham A, Sharath Babu P, Vidya Sagar J, Krishna D. Effect of reactive oxygen species on cholinergic receptor function. *Indian J Pharmacol* 2005; **6**:366–370.
4 Davies KJ, Sevanian A, Muakkassah-Kelly SF, Hochstein P. Uric acid–iron ion complexes. A new aspect of the antioxidant functions of uric acid. *Biochem J* 1986; **235**:747–754.
5 Whiteman M, Ketsawatsakul U, Halliwell B. A reassessment of the peroxynitrite scavenging activity of uric acid. *Ann N Y Acad Sci* 2002; **962**:242–259.
6 Chen W, Feng L, Huang Z, Su H. Hispidin produced from *Phellinus linteus* protects against peroxynitrite-mediated DNA damage and hydroxyl radical generation. *Chem Biol Interact* 2012; **199**:137–142.
7 Hooper DC, Spitsin S, Kean RB, Champion JM, Dickson GM, Chaudhry I, Koprowski H. Uric acid, a natural scavenger of peroxynitrite, in experimental allergic encephalomyelitis and multiple sclerosis. *Proc Natl Acad Sci USA* 1998; **95**:675–680.
8 Koprowski H, Spitsin SV, Hooper DC. Prospects for the treatment of multiple sclerosis by raising serum levels of uric acid, a scavenger of peroxynitrite. *Ann Neurol* 2001; **49**:139.
9 Miller A, Glass-Marmor L, Abraham M, Grossman I, Shapiro S, Galboiz Y. Bio-markers of disease activity and response to therapy in multiple sclerosis. *Clin Neurol Neurosurg* 2004; **106**:249–254.
10 Drachman DB. Myasthenia gravis. *N Engl J Med* 1994; **330**: 1797–1810.
11 Jaretzki A 3rd, Barohn RJ, Ernstoff RM, Kaminski HJ, Keesey JC, Penn AS, *et al.* Myasthenia gravis: recommendations for clinical research standards. Task Force of the Medical Scientific Advisory Board of the Myasthenia Gravis Foundation of America. *Ann Thorac Surg* 2000; **70**:327–334.
12 Zoccolella S, Tortorella C, Iaffaldano P, Direnzo V, D'Onghia M, Luciannatelli E, *et al.* Low serum urate levels are associated to female gender in multiple sclerosis patients. *PLoS One* 2012; **7**:e40608.
13 Luo J, Lindstrom J. AChR-specific immunosuppressive therapy of myasthenia gravis. *Biochem Pharmacol* 2015; **97**:609–619.
14 Park H, Bourla AB, Kastner DL, Colbert RA, Siegel RM. Lighting the fires within: the cell biology of autoinflammatory diseases. *Nat Rev Immunol* 2012; **12**:570–580.
15 Krishnaswamy A, Cooper E. Reactive oxygen species inactivate neuronal nicotinic acetylcholine receptors through a highly conserved cysteine near the intracellular mouth of the channel: implications for diseases that involve oxidative stress. *J Physiol* 2012; **590** (Pt 1):39–47.
16 Glantzounis GK, Tsimoyiannis EC, Kappas AM, Galaris DA. Uric acid and oxidative stress. *Curr Pharm Des* 2005; **11**:4145–4151.
17 Moccia M, Lanzillo R, Costabile T, Russo C, Carotenuto A, Sasso G, *et al.* Uric acid in relapsing-remitting multiple sclerosis: a 2-year longitudinal study. *J Neurol* 2015; **262**:961–967.

18 Oh SI, Baek S, Park JS, Piao L, Oh KW, Kim SH. Prognostic role of serum levels of uric acid in amyotrophic lateral sclerosis. *J Clin Neurol* 2015; **11**:376–382.
19 Peng F, Zhong X, Deng X, Qiu W, Wu A, Long Y, *et al*. Serum uric acid levels and neuromyelitis optica. *J Neurol* 2010; **257**:1021–1026.
20 von Geldern G, Mowry EM. The influence of nutritional factors on the prognosis of multiple sclerosis. *Nat Rev Neurol* 2012; **8**:678–689.
21 Hooper DC, Scott GS, Zborek A, Mikheeva T, Kean RB, Koprowski H, Spitsin SV. Uric acid, a peroxynitrite scavenger, inhibits CNS inflammation, blood–CNS barrier permeability changes, and tissue damage in a mouse model of multiple sclerosis. *FASEB J* 2000; **14**:691–698.
22 Fuhua P, Xuhui D, Zhiyang Z, Ying J, Yu Y, Feng T, *et al*. Antioxidant status of bilirubin and uric acid in patients with myasthenia gravis. *Neuroimmunomodulation* 2012; **19**:43–49.
